# Synthesis and crystal structure of two manganese-based 12-metallacrown-4 complexes: Na_2_(3-chloro­benzoate)_2_[12-MC_Mn(III)N(shi)_-4](DMF)_6_ and MnNa(3-chloro­benzoate)_3_[12-MC_Mn(III)N(shi)_-4](DMF)(H_2_O)_4_·4DMF·0.72H_2_O

**DOI:** 10.1107/S2056989020006362

**Published:** 2020-05-19

**Authors:** Curtis M. Zaleski, Matthias Zeller

**Affiliations:** a Department of Chemistry and Biochemistry, Shippensburg University, Shippensburg, PA 17257, United States; b Department of Chemistry, Purdue University, West Lafayette, Indiana 479070, United States

**Keywords:** metallacrown, manganese complex, salicyl­hydroximate, crystal structure

## Abstract

Similar synthetic schemes yield two different metallacrown (MC) complexes: bis­(μ-3-chloro­benzoato)hexa­kis­(di­methyl­formamide)­tetra­kis­(μ_4_-*N*,2-dioxido­benzene-1-carboximidato)tetra­manganese(III)disodium(I), [Mn_4_Na_2_(C_7_H_4_ClO_2_)_2_(C_7_H_4_NO_3_)_4_(C_3_H_7_NO)_6_] or Na_2_(3-chloro­benzoate)_2_[12-MC_Mn(III)N(shi)_-4](DMF)_6_, **1**, and tetra-μ-aqua-tris­(μ-3-chloro­benzoato)(di­methyl­formamide)­tetra­kis­(μ_4_-*N*,2-dioxido­benzene-1-carboximidato)penta­manganese(III)sodi­um(I) di­methyl­formamide tetra­solvate 0.72-hydrate, [Mn_5_Na(C_7_H_4_ClO_2_)_3_(C_7_H_4_NO_3_)_4_(C_3_H_7_NO)(H_2_O)_4_]·4C_3_H_7_NO·0.718H_2_O or MnNa(3-chloro­benzo­ate)_3_[12-MC_Mn(III)N(shi)_-4](DMF)(H_2_O)_4_·4DMF·0.72H_2_O, **2**, where shi^3−^ is salicyl­hydrox­imate and DMF is *N*,*N*-di­methyl­formamide. Both complexes have the same framework consisting of four Mn^III^ ions in the MC ring and four shi^3−^ ligands, resulting in an overall square-shaped mol­ecule. The Mn^III^ ions are either five- or six-coordinate with elongated bond lengths in the apical or axial direction, respectively. The structure of **1** is nearly planar, and the MC binds two Na^+^ ions on opposite faces of the MC central cavity. The 3-chloro­benzoate anions also bind on opposite faces of the MC and form bridges between the central Na^+^ ions and the ring Mn^III^ ions. For **1** the metallacrown mol­ecule, except for the central Na^+^ ion, exhibits whole mol­ecule disorder over two sets of sites. Both moieties are centrosymmetric and are related to each other by a pseudo-mirror operation with opposite sense of rotation around the Na⋯Na axis. The occupancy ratio of the main disorder of the metallacrown mol­ecules and 3-chloro­benzoate anions refined to 0.9276 (9):0.0724 (9). The structure of **2** is slightly domed, and the MC binds both an Mn^II^ ion and an Na^+^ ion in the MC central cavity. The Mn^II^ ion is located on the convex side of the MC, while the Na^+^ ion binds to the concave side. Complex **2** represents the first instance of a [12-MC_Mn(III)N(shi)_-4] mol­ecule binding both 3*d* transition metal and alkali metal ions in the central cavity. In addition, three 3-chloro­benzoate anions bind on the convex side of the MC and connect the Mn^II^ ion to three of the ring Mn^III^ ions.

## Abstract 2

The metallacrown (MC) complexes Na_2_(3-chloro­benzoate)_2_[12-MC_Mn(III)_ _N(shi)_-4](DMF)_6_, **1**, and MnNa(3-chloro­benzoate)_3_[12-MC_Mn(III)_ _N(shi)_-4](DMF)(H_2_O)_4_·4DMF·0.72H_2_O, **2**, where shi^3−^ is salicyl­hydroximate and DMF is *N*,*N*-di­methyl­formamide, both have an overall square shape due to the presence of four ring Mn^III^ ions and four shi^3−^ ligands. The two MC complexes bind different cations in the central cavity of the mol­ecule: two Na^+^ ions in **1** and one Mn^II^ ion and one Na^+^ ion in **2**.

## Chemical context   

The first 12-metallacrown-4 complex synthesized, Mn(acetate)_2_[12-MC_Mn(III)N(shi)_-4], was based on the ligand salicyl­hydroxamic acid (H_3_shi) and manganese (Lah & Pecoraro, 1989 ▸). In this complex, four Mn^III^ ions are located in the metallacrown (MC) ring and an Mn^II^ ion is trapped in the central MC cavity produced by the four triply deprotonated salicyl­hydroximate (shi^3−^) ligands. The Mn^II^ ion is further bound by two acetate anions that serve to balance the charge of the mol­ecule and to bridge between the ring Mn^III^ ions and the central Mn^II^ ion. Since this initial report in 1989, the [12-MC_Mn(III)N(shi)_-4] framework has been used to encapsulate not only manganese(II) but also alkali, alkaline earth, and lanthanide ions in the MC cavity (Mezei *et al.*, 2007 ▸; Lah & Pecoraro, 1991 ▸; Koumousi *et al.*, 2011 ▸; Azar *et al.*, 2014 ▸). When only Na^+^ or K^+^ ions are incorporated into the [12-MC_Mn(III)N(shi)_-4] framework, the two metal ions and their counter-anions are typically bound on opposite faces of the MC (Gibney *et al.*, 1996 ▸). When lanthanide ions are bound to the MC cavity, four carboxyl­ate anions serve to tether the *Ln* ^III^ ion to the MC and typically an alkali metal ion is bound to the opposite face of the MC for charge balance (Travis *et al.*, 2015 ▸, 2016 ▸). Furthermore, the bridging acetate anion of the original Mn(acetate)_2_[12-MC_Mn(III)N(shi)_-4] mol­ecule can be substituted by other carboxyl­ate anions or even halide and pseudohalide anions (Gibney *et al.*, 1996 ▸; Kessissoglou *et al.*, 2002 ▸; Dendrinou-Samara *et al.*, 2005 ▸; Boron *et al.*, 2016 ▸) . This ability to substitute various components of the MC complex allows the properties of the mol­ecules to be tailored to a particular application. For instance, the single-mol­ecule magnet properties of a series of Dy*MX*_4_[12-MC_Mn(III)N(shi)_-4] complexes, where *M* is Na^+^ or K^+^ and *X* is either acetate, tri­methyl­acetate, benzoate, or salicylate, are dictated by the identity of the carboxyl­ate anion even though the structures of the mol­ecules are strikingly similar (Boron *et al.*, 2016 ▸). Moreover, [12-MC_Mn(III)N(shi)_-4] complexes can be used as building blocks to form larger structures. They can be linked together to form either dimeric and trimeric systems or one-dimensional chains, and some of these larger structures have SMM-like behavior (Mengle *et al.*, 2015 ▸; Zaleski *et al.*, 2015 ▸; Alaimo *et al.*, 2017 ▸; Wang *et al.*, 2019 ▸).

Herein we present the first use of a halogenated benzoate anion to serve as the bridging ligand between the central cavity metal ion and the ring metal ions for a [12-MC_Mn(III)N(shi)_-4] complex. The use of 3-chloro­benzoate leads to two different mol­ecules: Na_2_(3-chloro­benzoate)_2_[12-MC_Mn(III)N(shi)_-4](DMF)_6_, **1**, where DMF is *N,N*-di­methyl­form­­amide, and MnNa(3-chloro­benzoate)_3_[12-MC_Mn(III)N(shi)_-4](DMF)(H_2_O)_4_·4DMF·0.72H_2_O, **2**. Complex **1** is typical of other di-sodium MCs with the Na^+^ ions bonded to opposite faces of the MC. However, complex **2** represents a new structural motif in metallacrown chemistry. In **2** the central Mn^II^ ion is bonded to three carboxyl­ate anions as opposed to the typical number of two anions. This then facilitates the binding of an Na^+^ ion to the opposite face of the MC for charge-balance purposes. This is the first instance of a 3*d* transition metal ion and an alkali metal ion both binding to the central cavity of a 12-MC-4 complex.

## Structural commentary   

Both **1** and **2** are based on the same overall 12-MC-4 framework. Four salicyl­hydroximate ligands and four ring Mn ions combine to generate a Mn–N–O repeat unit that recurs four times in a cyclic fashion. The fused five- and six-membered rings of the shi^3−^ ligands place the metal ions at 90° relative to each other, giving an overall square-shaped mol­ecule. The ring Mn ions are either five- or six-coordinate in the structures, and the ligand atoms in the basal/equatorial planes are the same, consisting of *trans* six- and five-membered chelate rings: each six-membered chelate ring is formed by the phenolate oxygen atom and oxime nitro­gen atom of a shi^3−^ ligand and each five-membered chelate ring is formed by the carbonyl oxygen atom and the oxime oxygen atom of a different shi^3−^ ligand. The four Mn ions of the MC ring are assigned a 3+ oxidation state based on average bond lengths, the presence of elongated axial bond lengths typical of a high-spin *d* ^4^ electron configuration, bond-valence sum (BVS) values (Liu & Thorp, 1993 ▸), and overall charge-balance considerations (Table 1 ▸). The four Mn^III^ ions and four shi^3−^ ligands produce a neutral MC framework. The main differences between **1** and **2** are the metal ions bound to the central cavity and the number of the ancillary ligands that bind to the metal ions of the MC.
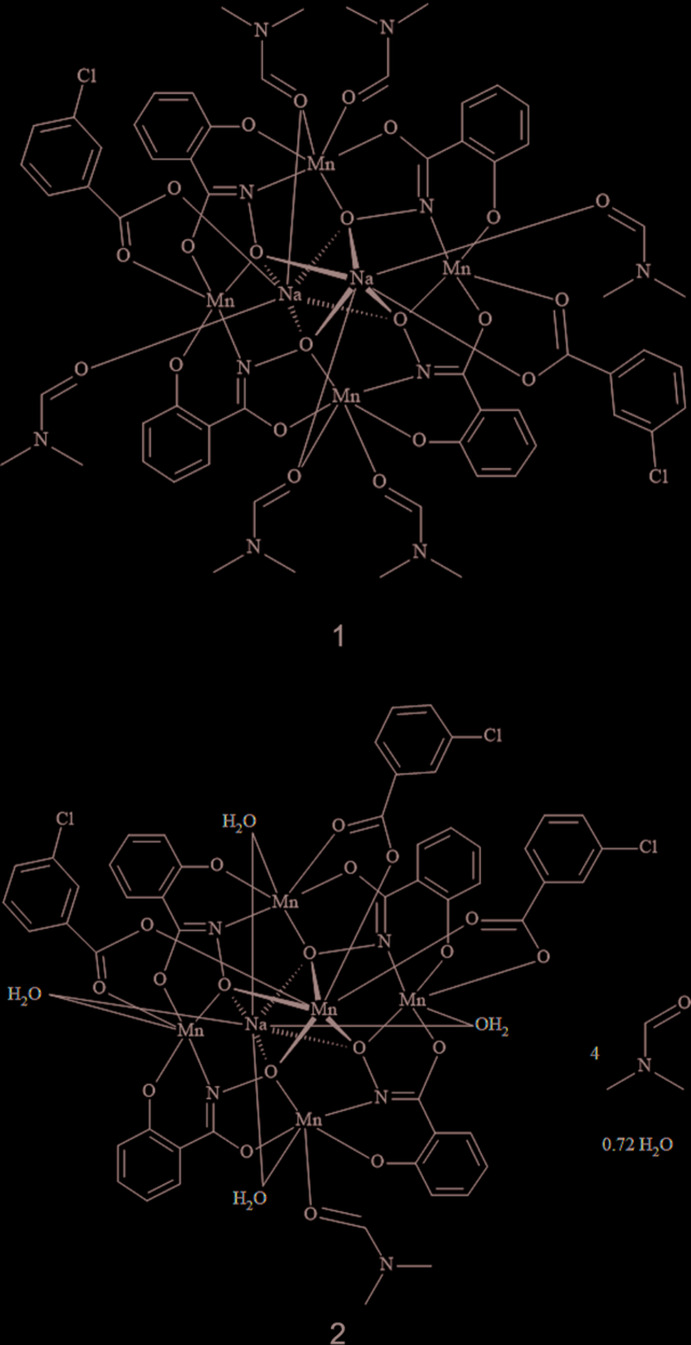


**Figure 1:**
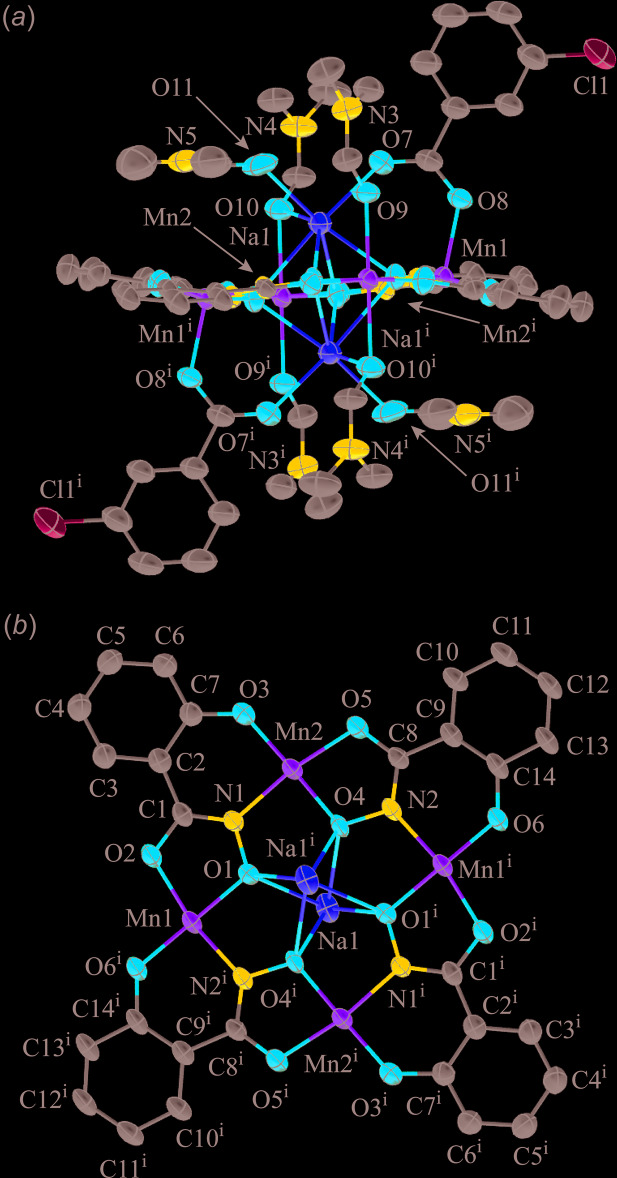
The single-crystal X-ray structure of Na_2_(3-chloro­benzoate)_2_[12-MC_Mn(III)N(shi)_-4](DMF)_6_, **1**, with displacement ellipsoids at the 50% probability level [symmetry code: (i) −*x* + 1, −*y* + 1, −*z* + 1]. (*a*) side view with only the metal atoms and heteroatoms of the axial ligands labelled for clarity and (*b*) top view with the axial ligand atoms omitted for clarity. In addition, hydrogen atoms and disorder have been omitted for clarity. Color scheme: green – Mn, yellow – sodium, red – oxygen, dark blue – nitro­gen, gray – carbon, and light blue – chlorine. All figures were generated with the program *Mercury* (Macrae *et al.*, 2020 ▸).

For **1** the MC framework (ring Mn^III^ ions and shi^3−^ ligands) and the 3-chloro­benozate anions exhibit whole-mol­ecule disorder over two sets of sites. Both moieties are centrosymmetric and are related to each other by a pseudo-mirror operation with an opposite sense of rotation around the Na⋯Na axis. The occupancy ratio of the MC frameworks and 3-chloro­benzoate anions disorder refined to 0.9276 (9):0.0724 (9). In addition, the coordinated DMF mol­ecules show disorder as outlined in the *Refinement* section below. Thus, only the structures of the main moieties will be discussed. The MC framework is nearly planar, and the MC cavity, produced by the four oxime oxygen atoms of the four shi^3−^ ligands, captures two Na^+^ ions on opposite faces of the MC (Fig. 1 ▸). The charge of the Na^+^ ions is balanced by two 3-chloro­benzoate anions that are also located on opposite faces of the MC. Each 3-chloro­benzoate connects one Na^+^ to a ring Mn^III^ ion (Mn1). The Na^+^ ion (Na1) is seven-coordinate, and the coordination environment consists of the four oxime oxygen atoms, a carboxyl­ate oxygen atom from a 3-chloro­benzoate anion, a carbonyl oxygen atom of a terminal DMF mol­ecule, and a μ-carbonyl oxygen atom of a DMF mol­ecule that also bridges to Mn2 of the MC ring. A SHAPE (*SHAPE 2.1*; Llunell *et al.*, 2013 ▸) analysis (Table 2 ▸) of the geometry yields the lowest continuous shape measure (CShM) values for a face-capped octa­hedron and a face-capped trigonal prism, 3.683 and 3.798, respectively (Llunell *et al.*, 2013 ▸; Pinsky & Avnir, 1998 ▸; Casanova *et al.*, 2004 ▸; Cirera *et al.*, 2005 ▸). Although the CShM value is lower for the face-capped octa­hedron, it is difficult to accurately assign the geometry as both CShM values are relatively close. In addition, both CShM values are well over 3.0, which is considered an upper threshold value at which significant distortions occur (Cirera *et al.*, 2005 ▸). The distortions may arise from the bonding nature of the MC framework. The four oxime oxygens of the MC cavity lie nearly in a plane due to the square shape of the mol­ecule imposed by the fused chelate rings of the shi^3−^ ligands. Thus, this portion of the coordination environment is not flexible and likely leads to the distortion. Mn1 of the MC ring is five-coordinate with a basal ligand environment as described above. A carboxyl­ate oxygen atom of a 3-chloro­benzoate anion occupies the apical position. A SHAPE analysis (Table 3 ▸) reveals the geometry can be best described as square-pyramidal and the calculated tau (τ) value of 0.15 supports this assignment, where τ = 0 for an ideal square pyramid and 1.0 for an ideal trigonal prism (Addison *et al.*, 1984 ▸). Mn2 is six-coordinate with an elongated Jahn–Teller axis, and the SHAPE analysis confirms a tetra­gonally distorted octa­hedral geometry (Table 4 ▸). The ligands along the axial axis consist of two carbonyl oxygen atoms of two DMF mol­ecules. The DMF mol­ecule associated with O9 binds in a terminal fashion, while the oxygen atom (O10) of the second DMF mol­ecule forms a one-atom μ-bridge to the central Na^+^ ion.

**Figure 2:**
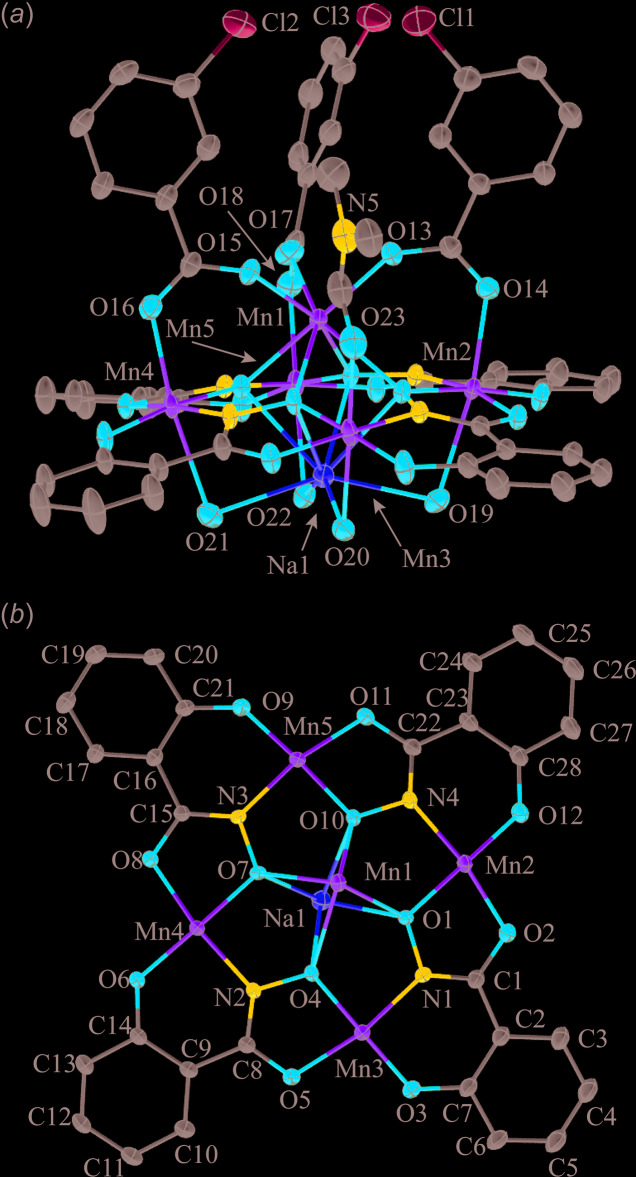
The single-crystal X-ray structure of MnNa(3-chloro­benzoate)_3_[12-MC_Mn(III)N(shi)_-4](DMF)(H_2_O)_4_·4DMF·0.72H_2_O, **2**, with displacement ellipsoids at the 50% probability level. (*a*) side view with only the metal atoms and heteroatoms of the axial ligands labelled for clarity and (*b*) top view with the axial ligand atoms omitted for clarity. In addition, the lattice DMF mol­ecules, partially occupied water mol­ecule, hydrogen atoms, and disorder have been omitted for clarity. See Fig. 1 ▸ for additional display details.

For **2** the MC is slightly domed with an Mn ion and Na ion bonded to opposite sides of the MC cavity. The Mn ion is bound on the convex side of the MC, and the Na ion is bonded to the concave side (Fig. 2 ▸). The Mn1 ion is assigned a 2+ oxidation state based an average bond length of 2.256 Å, a BVS value of 1.97 valence units (v. u.), and overall charge-balance considerations (Table 1 ▸). The total 3+ charge of the Mn^II^ and Na^+^ ions is counterbalanced by the presence of three 3-chloro­benzoate anions. The 3-chloro­benzoate anions bridge between Mn1 and three of the ring Mn^III^ ions (Mn2, Mn4, and Mn5). The Mn^II^ ion is seven-coordinate with a coordination environment consisting of four oxime oxygen atoms from four different shi^3−^ ligands and of three carboxyl­ate oxygen atoms from three different 3-chloro­benzoate anions. A SHAPE analysis of the geometry indicates that an unambiguous assignment is difficult as in the central Na^+^ ions in **1** (Table 5 ▸). The geometry is either face-capped octa­hedral (CShM = 1.589) or face-capped trigonal prismatic (CShM = 1.807). The Na^+^ ion of **2** is eight-coordinate with four oxime oxygen atoms from the shi^3−^ ligands and four water mol­ecules. The SHAPE analysis indicates that the geometry can best be described as a biaugmented trigonal prism, where two of the rectangular faces of a trigonal prism are capped by an atom (Table 6 ▸). All of the ring Mn^III^ ions are six-coordinate with an elongated Jahn–Teller axis. The SHAPE analysis confirms a tetra­gonally distorted octa­hedral geometry for each Mn^III^ ion (Table 7 ▸). The axial ligands of Mn2, Mn4, and Mn5 consist of a carboxyl­ate oxygen atom from a 3-chloro­benzoate anion and an oxygen atom of a water mol­ecule that forms a one-atom μ-bridge to the Na^+^ ion. The axial ligands of Mn3 are a carbonyl oxygen atom of a terminal DMF mol­ecule and also an oxygen atom of a water mol­ecule that forms a one-atom μ-bridge to the Na^+^ ion. Lastly, there are four DMF mol­ecules located in the lattice. One of the DMF mol­ecules (associated with O27) is disordered due to the presence of a partially occupied water mol­ecule [0.718 (6) occupancy]. The occupancy ratio of the disordered DMF mol­ecule refined to 0.718 (6):0.282 (6).

## Supra­molecular features   

**Figure 3:**
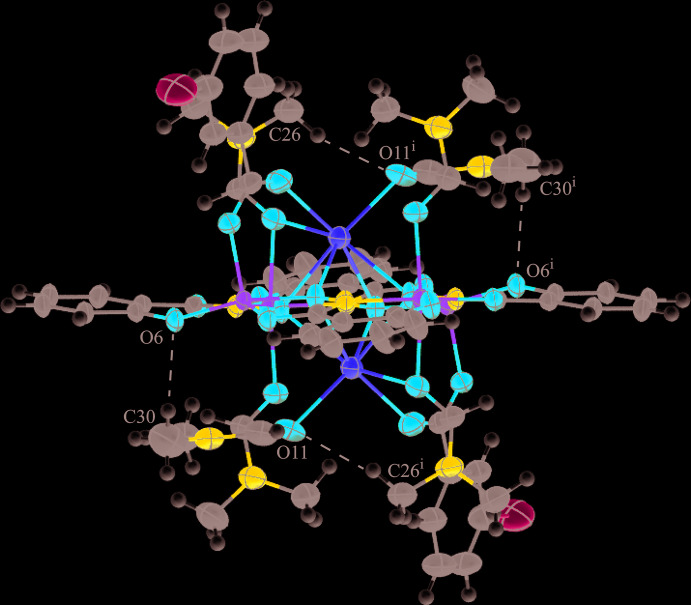
Intra­molecular C—H⋯O inter­actions in **1** between the hydrogen atoms (white) of the methyl groups of the coordinated DMF mol­ecules and the MC [symmetry code: (i) −*x* + 1, −*y* + 1, −*z* + 1]. For clarity the disorder has been omitted and only the atoms involved in the hydrogen bonding have been labeled. See Fig. 1 ▸ for additional display details.

**Figure 4:**
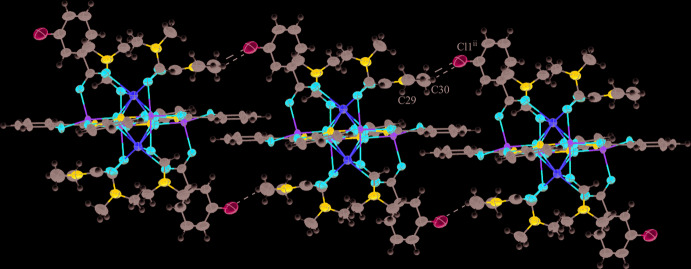
Inter­molecular C—H⋯Cl inter­actions in **1** between the hydrogen atoms (white) of the methyl groups of the DMF associated with O11 and the chlorine atom of the neighboring 3-chloro­benzoate anion [symmetry code: (ii) *x* + 1, *y*, *z* + 1]. The inter­actions result in a one-dimensional network. For clarity the disorder has been omitted and only the atoms involved in the inter­actions have been labeled.

For **1**, there are two intra­molecular C—H⋯O inter­actions and their symmetry equivalents per mol­ecule (Table 8 ▸): one inter­action is between a methyl group of a coordinated DMF mol­ecule to a carbonyl oxygen atom of a second coordinated DMF mol­ecule [C26—H26*A*⋯O11^i^; symmetry code: (i) −*x* + 1, −*y* + 1, −*z* + 1] and the other inter­action is between a methyl group of a coordinated DMF mol­ecule and a phenolate oxygen atom of a shi^3−^ ligand (C30—H30*C*⋯O6) (Fig. 3 ▸). No strong directional inter­molecular forces are observed between the mol­ecules of **1**; however, there are a few weak inter­molecular C—H⋯Cl inter­actions between the methyl groups of a coordinated DMF mol­ecule (associated with O11) and the chlorine atoms of 3-chloro­benzoate anions of neighboring MCs (Table 8 ▸; Fig. 4 ▸). These inter­actions generate a one-dimensional network, and these inter­actions, in addition to pure van der Waals forces, contribute to the overall packing of the mol­ecules.

**Figure 5:**
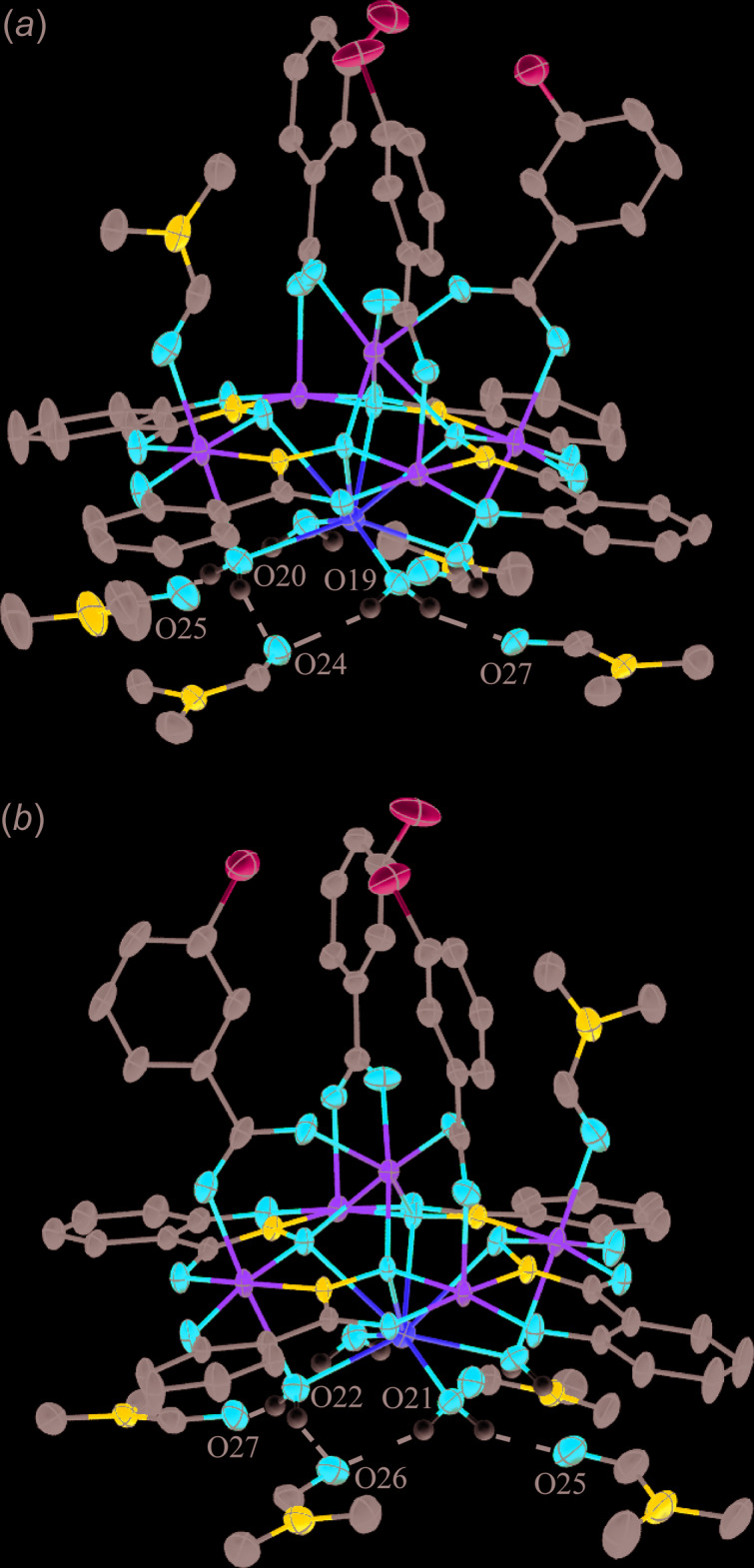
Inter­molecular hydrogen bonding in **2** between the water mol­ecules coordinated to the Na^+^ ion and the carbonyl oxygen atoms of the lattice DMF mol­ecules. For clarity the inter­actions have been divided into two sections (*a*) and (*b*), only the hydrogen atoms (white) of the water mol­ecules have been included, the disorder and the partially occupied water mol­ecule have been omitted, and only the atoms involved in the inter­actions have been labeled. See Fig. 1 ▸ for additional display details.

**Figure 6:**
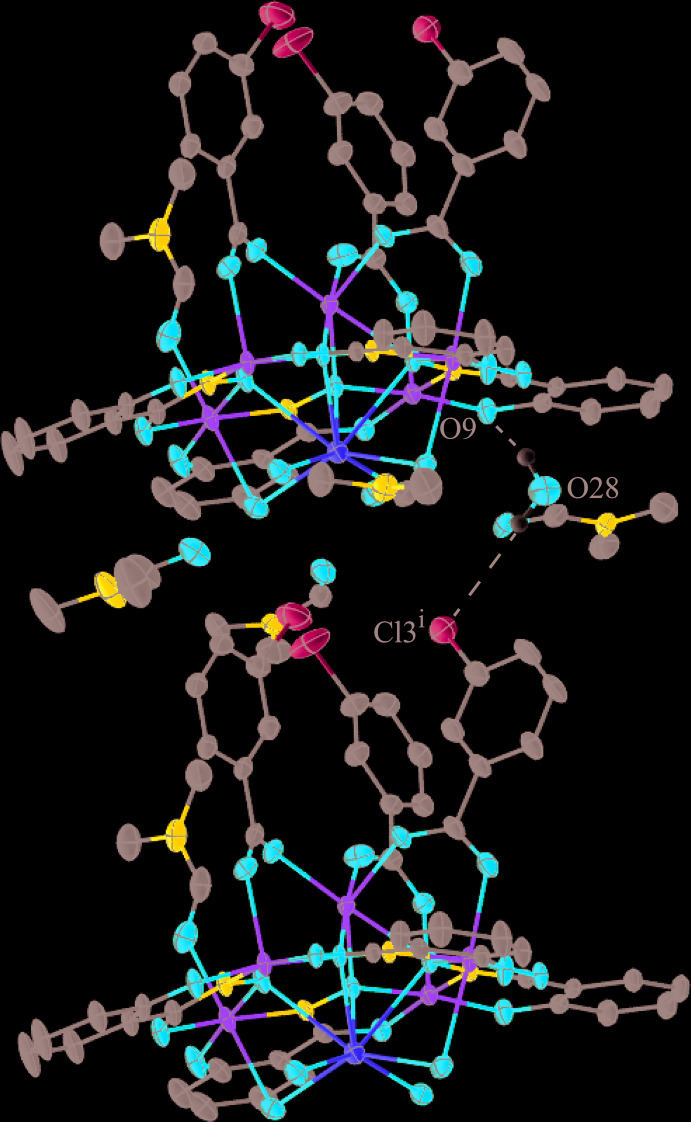
Inter­molecular hydrogen bonding in **2** between the partially occupied water mol­ecule and two neighboring MCs [symmetry code: (i) *x* − 1, *y*, *z*]. For clarity only the hydrogen atoms (white) of the water mol­ecule associated with O28 have been included and only the atoms involved in the inter­action have been labeled. See Fig. 1 ▸ for additional display details.

For **2** no strong directional inter­molecular inter­actions are observed between the mol­ecules, but several hydrogen bonds exist between the water mol­ecules coordinated to the Na^+^ ion and the carbonyl oxygen atoms of the DMF mol­ecules located in the lattice (Table 9 ▸; Fig. 5 ▸). In addition, the partially occupied water mol­ecule associated with O28 is hydrogen bonded to the phenolate oxygen atom of a shi^3−^ ligand of one MC and to the chlorine atom of a 3-chloro­benzoate ligand of a neighboring MC (Fig. 6 ▸). These hydrogen-bonding inter­actions, in addition to pure van der Waals forces, contribute to the overall packing of the mol­ecules.

## Database survey   

A survey of the Cambridge Structural Database (CSD version 5.41, update March 2020, Groom *et al.*, 2016 ▸) reveals that there are 61 different structures with the [12-MC_Mn(III)N(shi)_-4] framework as either a discrete mol­ecule or as a building block for a larger structure. Of those compounds, there are eight di-sodium MCs as in **1** and only five MCs with a central man­ganese ion as in **2**. For the eight Na_2_ *X*_2_[12-MC_Mn(III)N(shi)_-4] structures, six MCs exist as individual mol­ecules with the counter-anions (*X*) of chloride (JILLOF; Lah & Pecoraro, 1991 ▸), bromide (TOXNID; Gibney *et al.*, 1996 ▸), thio­cyanate (UFIXOW; Kessissoglou *et al.*, 2002 ▸), acetate (TIWWON; Azar *et al.*, 2014 ▸), chloro­acetate (ZOQTUW; Daly *et al.*, 2014 ▸), and butyrate (DUCWAB; Mengle *et al.*, 2015 ▸). The remaining two MCs are one-dimensional chains of Na_2_ *X*_2_[12-MC_Mn(III)N(shi)_-4] complexes with either propionate (DUCWIJ) or butyrate (DUCWEF) serving as linkers between the di-sodium MCs (Mengle *et al.*, 2015 ▸).

For the five Mn*X*_2_[12-MC_Mn(III)N(shi)_-4] structures, the bridg­ing ligands between the central cavity Mn^II^ ions and the ring Mn^III^ ions include two acetate-based MCs (SEDBOS; Lah & Pecoraro, 1989 ▸; TODGAX; Marzaroli *et al.*, 2019 ▸), a benzoate-based MC (FILGAJ, Dendrinou-Samara *et al.*, 2005 ▸), a MC with formate ions that bind to the central cavity Mn^II^ ion and two 2-(2,4-di­chloro­phen­oxy)propionate ions that bind to another Mn^II^ ion located above the central cavity Mn^II^ ion (IDUYUB; Dendrinou-Samara *et al.*, 2001 ▸), and one MC dimer with both acetate and 1,2,4-triazolate anions (ZUCYAZ; Zaleski *et al.*, 2015 ▸). None of these structures contains an Na^+^ ion opposite the Mn^II^ ion; thus, complex **2** is the first example of a [12-MC_Mn(III)N(shi)_-4] that binds both a 3*d* transition metal ion and an Na^+^ ion in the central cavity along with three bridging carboxyl­ate-based ligands. Lastly, there are nineteen [12-MC_Mn(III)N(shi)_-4] structures (CSD version 5.41, update March 2020, Groom *et al.*, 2016 ▸) with both *Ln* ^III^ ions and Na^+^ ions bound in the central cavity (Azar *et al.*, 2014 ▸; Boron *et al.*, 2016 ▸;, Travis *et al.*, 2016 ▸; Wang *et al.*, 2019 ▸) and two examples of a [12-MC_Mn(III)N(shi)_-4] complex binding both the 4*d* transition metal ion Y^III^ and a Na^+^ ion (TIWWIH; Azar *et al.*, 2014 ▸; WUQNUT; Travis *et al.*, 2015 ▸).

## Synthesis and crystallization   

**Materials**

Manganese(II) acetate tetra­hydrate (99+%) and 3-chloro­benzoic acid (99+%) were purchased from Acros Organics. Salicyl­hydroxamic acid (99%) was purchased from Alfa Aesar. Sodium hydroxide (Certified ACS grade) was purchased from Fisher Scientific. *N*,*N*-Di­methyl­formamide (DMF, Certified ACS grade) was purchased from BDH Chemicals. All reagents were used as received without further purification.

**Synthesis of Na_2_(3-chloro­benzoate)_2_[12-MC_Mn(III)N(shi)_-4](DMF)_6_, 1.**

Sodium hydroxide (0.1710 g, 4 mmol) and 3-chloro­benzoic acid (0.6271 g, 4 mmol) were mixed in 8 mL of DMF resulting in a clear and colorless solution. The NaOH did not com­pletely dissolve. In a separate vessel, salicyl­hydroxamic acid (H_3_shi; 0.3063 g, 2 mmol) was dissolved in 8 mL of DMF resulting in a clear and slightly yellow solution. In a third vessel, manganese(II) acetate tetra­hydrate (0.4907 g, 2 mmol) was dissolved in 8 mL of DMF resulting in a light-orange solution. The manganese(II) acetate solution was added to the H_3_shi solution resulting in a dark-brown color. The sodium hydroxide/3-chloro­benzoic acid mixture was then immediately added and no color change was observed. The solution was stirred overnight and then gravity filtered the next day. A dark-brown precipitate was recovered and discarded. Also, it was observed that not all of the NaOH had dissolved after stirring overnight. The filtrate was a dark-brown solution that was left for slow evaporation at room temperature. After seven days, dark-brown/black plate-shaped crystals were collected for X-ray analysis. The remaining crystals were collected, washed with cold DMF, and dried. The percentage yield of the reaction was 1% (0.0080 g, 0.0050 mmol) based on mangan­ese(II) acetate tetra­hydrate.

**Synthesis of MnNa(3-chloro­benzoate)_3_[12-MC_Mn(III)N(shi)_-4](DMF)(H_2_O)_4_·4DMF·0.72H_2_O, 2.**

The stoichiometric ratios between the reactants and the volume of DMF were the same as for **1** with slightly different masses of the reactants: sodium hydroxide (0.1627 g, 4 mmol), 3-chloro­benzoic acid (0.6267 g, 4 mmol), H_3_shi (0.3072 g, 2 mmol), and manganese(II) acetate tetra­hydrate (0.4914 g, 2 mmol). In addition, the mixing order was altered: the sodium hydroxide/3-chloro­benzoic acid mixture was first added to the H_3_shi solution, followed by the addition of the manganese(II) acetate solution. Furthermore, when the solution was filtered after stirring overnight, no precipitate was recovered. It was also observed that not all of the NaOH had dissolved. The filtrate was a dark-brown solution that was left for slow evaporation at room temperature. After three days, dark-brown/black plate crystals were collected for X-ray analysis. The remaining crystals were collected, washed with cold DMF, and dried. The percentage yield of the reaction was 35% (0.2543 g, 0.1401 mmol) based on manganese(II) acetate tetra­hydrate.

## Refinement   

For **1**, the metallacrown mol­ecule, except the central Na, exhibits whole mol­ecule disorder over two sets of sites. Both moieties are centrosymmetric and are related to each other by a pseudo-mirror operation with opposite sense of rotation around the Na⋯Na axis. The DMF mol­ecules of O9 and O10 of the major moiety are additionally disordered. The DMF mol­ecule associated with O11 was found to be disordered independently from the main disorder.

To assist in the refinement of the disorder, the geometries of the two metallacrowns (Mn and salicyl­hydroximate ligands), of the 3-chloro­benzoate anions, and of each DMF mol­ecule were restrained to be similar to their disordered partner(s) (esd = 0.02 Å, SAME commands in *SHELXL*). The distances between Mn2 and O9 and Mn2*B* and O9*B* were restrained to be similar (esd = 0.02 Å; SADI restraint in *SHELXL*). All atoms of the minor moiety of the 3-chloro­benzoate (C15*B*–C21*B*, Cl1*B*) as well as of the minor disordered DMF mol­ecules of O10 (associated with O10*B* and O10*C*) were restrained to lie in the same plane (esd = 0.01 Å; FLAT restraint in *SHELXL*). All disordered atoms were restrained to have similar *U^ij^* components of their ADPs (esd = 0.01 Å^2^; SIMU restraint in *SHELXL*). The ADPs of C11 and C11*B* of a salicyl­hydroximate were constrained to be identical. Lastly, occupancies were constrained to sum up to unity for all sites using SUMP commands. Subject to the above conditions, the occupancy ratio of the main disorder of the metallacrown mol­ecules and 3-chloro­benzoate anions refined to 0.9276 (9):0.0724 (9). The occupancy rates for the additionally split DMF of O9 refined to 0.799 (3) (O9) and 0.129 (3) (O9*C*), and those of the additionally split DMF mol­ecule of O10 refined to 0.498 (3) (O10) and 0.430 (3) (O10*C*). The occupancy ratio of the DMF mol­ecules associated with O11 refined to 0.516 (5):0.484 (5).

For **2**, a partially occupied water mol­ecule (O28) induces disorder for a neighboring DMF mol­ecule (of O27). The two disordered moieties were restrained to have similar geometries, and the carbon, oxygen, and nitro­gen atoms of the DMF mol­ecule restrained to have similar *U^ij^* components of the ADPs (esd = 0.01 Å^2^; SIMU restraint in *SHELXL*). Subject to these conditions the occupancy ratio refined to 0.718 (6):0.282 (6). Water hydrogen-atom positions were refined and O—H and H⋯H distances were restrained to 0.84 (2) and 1.36 (2) Å, respectively. The water hydrogen-atom positions of partially occupied O28 were further restrained based on hydrogen-bonding considerations.

For **1** and **2**, all other hydrogen atoms were placed in calculated positions and refined as riding on their carrier atoms with C—H distances of 0.95 Å for *sp* ^2^ carbon atoms and 0.98 Å for methyl carbon atoms. The *U*_iso_ values for hydrogen atoms were set to a multiple of the value of the carrying carbon atom (1.2 times for *sp* ^2^-hybridized carbon atoms or 1.5 times for methyl carbon atoms). Additional crystallographic data and experimental parameters are provided in Table 10 ▸ and in the CIF.

## Supplementary Material

Crystal structure: contains datablock(s) 1, 2. DOI: 10.1107/S2056989020006362/mw2160sup1.cif

Structure factors: contains datablock(s) 1. DOI: 10.1107/S2056989020006362/mw21601sup2.hkl

Structure factors: contains datablock(s) 2. DOI: 10.1107/S2056989020006362/mw21602sup3.hkl

CCDC references: 2003408, 2003407

Additional supporting information:  crystallographic information; 3D view; checkCIF report

## Figures and Tables

**Table 1:** Average bond-length (Å) and bond-valence-sum (BVS) values (v. u.) used to support assigned oxidation states of the manganese ions of **1** and **2**

	Avg. bond length	BVS value	Assigned oxidation state
**1**			
Mn1	1.944	3.05	3+
Mn2	2.027	3.12	3+
			
**2**			
Mn1	2.256	1.97	2+
Mn2	2.054	3.07	3+
Mn3	2.044	3.10	3+
Mn4	2.053	3.07	3+
Mn5	2.044	3.07	3+

**Table 2:** Continuous shape measurement (CShM) values (*SHAPE 2.1*) for the seven-coordinate sodium ion of **1**

Heptagon	34.157
Hexagonal pyramid	19.758
Penta­gonal bipyramid	8.496
Capped octa­hedron	3.683
Capped trigonal prism	3.798
Johnson penta­gonal bipyramid	12.331
Johnson elongated triangular pyramid	21.938

**Table 3:** Continuous shape measurement (CShM) values (*SHAPE 2.1*) for the five-coordinate manganese ion of **1**

Penta­gon	28.077
Vacant octa­hedron	1.468
Trigonal bipyramid	4.930
Square pyramid	0.712
Johnson trigonal bipyramid	8.311

**Table 4:** Continuous shape measurement (CShM) values (*SHAPE 2.1*) for the six-coordinate manganese ion of **1**

Hexagon	31.700
Penta­gonal pyramid	27.764
Octa­hedron	0.872
Trigonal prism	16.422
Johnson penta­gonal pyramid	30.863

**Table 5:** Continuous shape measurement (CShM) values (*SHAPE 2.1*) for the seven-coordinate manganese ion of **2**

Heptagon	32.707
Hexagonal pyramid	20.417
Penta­gonal bipyramid	5.626
Capped octa­hedron	1.589
Capped trigonal prism	1.807
Johnson penta­gonal bipyramid	9.086
Johnson elongated triangular pyramid	20.152

**Table 6:** Continuous shape measurement (CShM) values (*SHAPE 2.1*) for the eight-coordinate sodium ion of **2**

Octa­gon	30.163
Heptagonal pyramid	25.281
Hexagonal bipyramid	13.805
Cube	6.579
Square anti­prism	3.022
Triangular dodeca­hedron	3.398
Johnson gyrobifastigium	16.071
Johnson elongated triangular bipyramid	28.948
Johnson biaugmented trigonal prism	4.411
Biaugmented trigonal prism	2.764
Snub diphenoid	6.604
Triakis tetra­hedron	7.183
Elongated trigonal bipyramid	24.722

**Table 7:** Continuous shape measurement (CShM) values (*SHAPE 2.1*) for the six-coordinate manganese ions of **2**

	Mn2	Mn3	Mn4	Mn5
Hexagon	30.762	30.538	30.590	30.154
Penta­gonal pyramid	27.834	27.546	27.453	27.158
Octa­hedron	1.320	1.219	1.257	1.127
Trigonal prism	15.522	15.883	16.455	16.107
Johnson penta­gonal pyramid	30.664	29.844	30.430	30.100

**Table 8:** Hydrogen-bond geometry (Å, °) for **1** [Chem scheme1]

*D*—H⋯*A*	*D*—H	H⋯*A*	*D*⋯*A*	*D*—H⋯*A*
C26—H26*A*⋯O11^i^	0.98	2.65	3.56 (2)	155
C29—H29*A*⋯Cl1^ii^	0.98	2.78	3.702 (10)	156
C30—H30*A*⋯Cl1^ii^	0.98	2.79	3.699 (14)	154
C30—H30*C*⋯O6	0.98	2.54	3.125 (16)	119

Symmetry codes: (i) 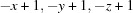; (ii) 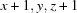.

**Table 9:** Hydrogen-bond geometry (Å, °) for **2** [Chem scheme1]

*D*—H⋯*A*	*D*—H	H⋯*A*	*D*⋯*A*	*D*—H⋯*A*
O19—H19*A*⋯O24	0.80 (2)	2.00 (3)	2.749 (3)	155 (5)
O19—H19*B*⋯O27	0.83 (2)	2.03 (3)	2.826 (7)	161 (5)
O20—H20*A*⋯O24	0.83 (2)	2.00 (3)	2.788 (3)	160 (5)
O20—H20*B*⋯O25	0.86 (2)	1.96 (3)	2.751 (4)	153 (4)
O21—H21*A*⋯O26	0.83 (2)	1.96 (3)	2.737 (3)	155 (5)
O21—H21*B*⋯O25	0.86 (2)	2.10 (4)	2.787 (3)	136 (4)
O22—H22*A*⋯O26	0.84 (2)	1.93 (3)	2.729 (3)	158 (5)
O22—H22*B*⋯O27	0.84 (2)	1.90 (3)	2.705 (6)	160 (5)
O28—H28*A*⋯Cl3^i^	0.92 (2)	2.96 (2)	3.876 (5)	171 (6)
O28—H28*B*⋯O9	0.90 (2)	2.14 (2)	3.019 (5)	165 (8)

Symmetry code: (i) 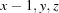.

**Table 10:** Experimental details

	**1**	**2**
Crystal data		
Chemical formula	[Mn_4_Na_2_(C_7_H_4_ClO_2_)_2_(C_7_H_4_NO_3_)_4_(C_3_H_7_NO)_6_]	[Mn_5_Na(C_7_H_4_ClO_2_)_3_(C_7_H_4_NO_3_)_4_(C_3_H_7_NO)(H_2_O)_4_]·4C_3_H_7_NO·0.718H_2_O
*M*_r_	1615.99	1815.30
Crystal system, space group	Triclinic, *P* 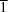	Monoclinic, *P* *n*
Temperature (K)	150	150
*a*, *b*, *c* (Å)	12.0423 (8), 12.3722 (8), 12.6875 (9)	14.1955 (9), 16.3349 (11), 16.6144 (10)
α, β, γ (°)	102.839 (3), 111.628 (3), 90.722 (4)	90, 94.235 (2), 90
*V* (Å^3^)	1704.0 (2)	3842.1 (4)
*Z*	1	2
Radiation type	Cu *K*α	Mo *K*α
μ (mm^−1^)	7.45	1.00
Crystal size (mm)	0.18 × 0.08 × 0.04	0.45 × 0.23 × 0.09

Data collection		
Diffractometer	Bruker AXS D8 Quest CMOS diffractometer with PhotonII charge-integrating pixel array detector (CPAD)	Bruker AXS D8 Quest CMOS diffractometer
Absorption correction	Multi-scan (*SADABS*; Krause *et al.*, 2015 ▸)	Multi-scan (*SADABS*; Krause *et al.*, 2015 ▸)
*T*_min_, *T*_max_	0.528, 0.754	0.636, 0.747
No. of measured, independent and observed [*I* > 2σ(*I*)] reflections	22753, 6871, 5590	118654, 27291, 24179
*R*_int_	0.048	0.038
(sin θ/λ)_max_ (Å^−1^)	0.640	0.771

Refinement		
*R*[*F* ^2^ > 2σ(*F* ^2^)], *wR*(*F* ^2^), *S*	0.045, 0.128, 1.12	0.034, 0.091, 1.03
No. of reflections	6871	27291
No. of parameters	999	1079
No. of restraints	2038	143
H-atom treatment	H-atom parameters constrained	H atoms treated by a mixture of independent and constrained refinement
Δρ_max_, Δρ_min_ (e Å^−3^)	0.49, −0.57	0.82, −0.69
Absolute structure	–	Flack *x* determined using 10010 quotients [(*I* ^+^)−(*I* ^−^)]/[(*I* ^+^)+(*I* ^−^)] (Parsons *et al.*, 2013 ▸)
Absolute structure parameter	–	0.000 (2)

Computer programs: *APEX3* and *SAINT* (Bruker, 2018 ▸), *SHELXS97* (Sheldrick, 2008 ▸), *SHELXL2018/3* (Sheldrick, 2015 ▸), *shelXle* (Hübschle *et al.*, 2011 ▸), *Mercury* (Macrae *et al.*, 2020 ▸) and *publCIF* (Westrip, 2010 ▸).

## supplementary crystallographic information

### Bis(µ-3-chlorobenzoato)hexakis(dimethylformamide)tetrakis(µ4-N,2-dioxidobenzene-1-carboximidato)tetramanganese(III)disodium(I) (1) Crystal data

**Table:**

[Mn_4_Na_2_(C_7_H_4_ClO_2_)_2_(C_7_H_4_NO_3_)_4_(C_3_H_7_NO)_6_]	*Z* = 1
*M**_r_* = 1615.99	*F*(000) = 828
Triclinic, *P*1	*D*_x_ = 1.575 Mg m^−^^3^
*a* = 12.0423 (8) Å	Cu *K*α radiation, λ = 1.54178 Å
*b* = 12.3722 (8) Å	Cell parameters from 9922 reflections
*c* = 12.6875 (9) Å	θ = 5.7–80.7°
α = 102.839 (3)°	µ = 7.45 mm^−^^1^
β = 111.628 (3)°	*T* = 150 K
γ = 90.722 (4)°	Plate, brown
*V* = 1704.0 (2) Å^3^	0.18 × 0.08 × 0.04 mm

### Bis(µ-3-chlorobenzoato)hexakis(dimethylformamide)tetrakis(µ4-N,2-dioxidobenzene-1-carboximidato)tetramanganese(III)disodium(I) (1) Data collection

**Table:**

Bruker AXS D8 Quest CMOS diffractometer with PhotonII charge-integrating pixel array detector (CPAD)	6871 independent reflections
Radiation source: I-mu-S microsource X-ray tube	5590 reflections with *I* > 2σ(*I*)
Laterally graded multilayer (Goebel) mirror monochromator	*R*_int_ = 0.048
Detector resolution: 7.4074 pixels mm^-1^	θ_max_ = 80.8°, θ_min_ = 5.1°
ω and phi scans	*h* = −15→13
Absorption correction: multi-scan (SADABS; Krause *et al.*, 2015)	*k* = −15→15
*T*_min_ = 0.528, *T*_max_ = 0.754	*l* = −16→16
22753 measured reflections	

### Bis(µ-3-chlorobenzoato)hexakis(dimethylformamide)tetrakis(µ4-N,2-dioxidobenzene-1-carboximidato)tetramanganese(III)disodium(I) (1) Refinement

**Table:**

Refinement on *F*^2^	Primary atom site location: structure-invariant direct methods
Least-squares matrix: full	Secondary atom site location: difference Fourier map
*R*[*F*^2^ > 2σ(*F*^2^)] = 0.045	Hydrogen site location: inferred from neighbouring sites
*wR*(*F*^2^) = 0.128	H-atom parameters constrained
*S* = 1.12	*w* = 1/[σ^2^(*F*_o_^2^) + (0.0687*P*)^2^ + 0.554*P*] where *P* = (*F*_o_^2^ + 2*F*_c_^2^)/3
6871 reflections	(Δ/σ)_max_ = 0.001
999 parameters	Δρ_max_ = 0.49 e Å^−^^3^
2038 restraints	Δρ_min_ = −0.57 e Å^−^^3^

### Bis(µ-3-chlorobenzoato)hexakis(dimethylformamide)tetrakis(µ4-N,2-dioxidobenzene-1-carboximidato)tetramanganese(III)disodium(I) (1) Special details

**Table:**

Geometry. All esds (except the esd in the dihedral angle between two l.s. planes) are estimated using the full covariance matrix. The cell esds are taken into account individually in the estimation of esds in distances, angles and torsion angles; correlations between esds in cell parameters are only used when they are defined by crystal symmetry. An approximate (isotropic) treatment of cell esds is used for estimating esds involving l.s. planes.
Refinement. The metallacrown molecule, except the central Na, exhibits whole molecule disorder over two sites. Both moieties are centrosymmetric and are related to each other by a pseudo-mirror operation with opposite sense of rotation around the Na···Na axis. The DMF molecules of O9 and O10 of the major moiety are additionally disordered. The DMF molecule associated with O11 was found to be disordered independently from the main disorder.To assist in the refinement of the disorder, the geometries of the two metallacrowns (Mn and salicylhydroximate ligands), of the 3-chlorobenzoates, and of each DMF molecule were restrained to be similar to their disordered partner(s) (esd = 0.02 Angstrom, SAME commands in Shelxl). The distances between Mn2 and O9 and Mn2B and O9B were restrained to be similar (esd = 0.02 Angstrom; SADI restraint in Shexl). All atoms of the minor moiety of the 3-chlorobenzoate (C15B-C21B, Cl1B) as well as of the minor disordered DMF molecules of O10 (associated with O10B and O10C) were restrained to lie in the same plane (esd = 0.01 Angstrom; FLAT restraint in Shexl). All disordered atoms were restrained to have similar Uij components of their ADPs (esd = 0.01 Angstrom squared; SIMU restraint in Shexl). The ADPs of C11 and C11B of a salicylhydroximate were constrained to be identical. Occupancies were constrained to sum up to unity for all sites using SUMP commands.Subject to the above conditions, the occupancy ratio of the main disorder of the metallacrown molecules and 3-chlorobenzoates refined to 0.9276 (9) to 0.0724 (9). The occupancy rates for the additionally split DMF of O9 refined to 0.799 (3) (O9) and 0.047 (3) (O9C), and those of the additionally split DMF molecule of O10 refined to 0.498 (3) (O10) and 0.430 (3) (O10C). The occupancy ratio of the DMF molecules associated with O11 refined to 0.516 (5) to 0.484 (5).All hydrogen atoms were placed in calculated positions and refined as riding on their carrier atoms with C-H distances of 0.95 Angstrom for sp2 carbon atoms and 0.98 Angstrom for methyl carbon atoms. The Uiso values for hydrogen atoms were set to a multiple of the value of the carrying carbon atom (1.2 times for sp2 hybridized carbon atoms or 1.5 times for methyl carbon atoms).

### Bis(µ-3-chlorobenzoato)hexakis(dimethylformamide)tetrakis(µ4-N,2-dioxidobenzene-1-carboximidato)tetramanganese(III)disodium(I) (1) Fractional atomic coordinates and isotropic or equivalent isotropic displacement parameters (Å^2^)

**Table:**

	*x*	*y*	*z*	*U*_iso_*/*U*_eq_	Occ. (<1)
Na1	0.56999 (10)	0.57176 (10)	0.43966 (10)	0.0414 (3)	
Mn1	0.24955 (4)	0.51893 (4)	0.28749 (3)	0.02874 (13)	0.9276 (9)
O1	0.36731 (18)	0.57424 (17)	0.43743 (16)	0.0344 (4)	0.9276 (9)
N1	0.3344 (2)	0.6609 (2)	0.5069 (2)	0.0305 (6)	0.9276 (9)
O2	0.15555 (19)	0.61826 (18)	0.35629 (17)	0.0362 (5)	0.9276 (9)
C1	0.2226 (3)	0.6782 (2)	0.4602 (2)	0.0314 (6)	0.9276 (9)
C2	0.1718 (3)	0.7641 (3)	0.5235 (3)	0.0335 (6)	0.9276 (9)
C3	0.0508 (3)	0.7792 (3)	0.4687 (3)	0.0402 (7)	0.9276 (9)
H3	0.005415	0.733099	0.393366	0.048*	0.9276 (9)
C4	−0.0042 (3)	0.8584 (3)	0.5202 (4)	0.0502 (9)	0.9276 (9)
H4	−0.086603	0.866715	0.481564	0.060*	0.9276 (9)
C5	0.0625 (4)	0.9261 (3)	0.6294 (4)	0.0550 (10)	0.9276 (9)
H5	0.025976	0.982396	0.665490	0.066*	0.9276 (9)
C6	0.1820 (3)	0.9124 (3)	0.6865 (3)	0.0479 (8)	0.9276 (9)
H6	0.225812	0.958890	0.762110	0.057*	0.9276 (9)
C7	0.2394 (3)	0.8323 (3)	0.6359 (3)	0.0348 (6)	0.9276 (9)
O3	0.3544 (2)	0.82499 (18)	0.69636 (19)	0.0408 (5)	0.9276 (9)
Mn2	0.45555 (4)	0.72084 (3)	0.66609 (4)	0.02969 (13)	0.9276 (9)
O4	0.55221 (18)	0.61023 (17)	0.63426 (16)	0.0354 (5)	0.9276 (9)
N2	0.6542 (3)	0.6066 (3)	0.7316 (2)	0.0311 (5)	0.9276 (9)
O5	0.58685 (18)	0.75588 (16)	0.81973 (17)	0.0341 (4)	0.9276 (9)
C8	0.6678 (3)	0.6852 (2)	0.8243 (2)	0.0282 (5)	0.9276 (9)
C9	0.7740 (3)	0.6954 (2)	0.9320 (2)	0.0311 (6)	0.9276 (9)
C10	0.7776 (3)	0.7720 (3)	1.0336 (3)	0.0376 (7)	0.9276 (9)
H10	0.710856	0.813058	1.030807	0.045*	0.9276 (9)
C11	0.8760 (4)	0.7888 (3)	1.1372 (3)	0.0433 (8)	0.9276 (9)
H11	0.877246	0.840970	1.205149	0.052*	0.9276 (9)
C12	0.9734 (3)	0.7283 (3)	1.1409 (3)	0.0411 (8)	0.9276 (9)
H12	1.041641	0.739355	1.211940	0.049*	0.9276 (9)
C13	0.9720 (3)	0.6520 (3)	1.0422 (3)	0.0371 (7)	0.9276 (9)
H13	1.039585	0.611952	1.045876	0.045*	0.9276 (9)
C14	0.8715 (3)	0.6334 (2)	0.9365 (2)	0.0327 (6)	0.9276 (9)
O6	0.87716 (18)	0.55919 (17)	0.84463 (17)	0.0351 (4)	0.9276 (9)
O7	0.4914 (2)	0.6448 (2)	0.2744 (2)	0.0493 (6)	0.9276 (9)
O8	0.2903 (2)	0.62890 (18)	0.20310 (19)	0.0416 (5)	0.9276 (9)
C15	0.3932 (3)	0.6717 (3)	0.2175 (3)	0.0425 (8)	0.9276 (9)
C16	0.3907 (4)	0.7686 (3)	0.1615 (3)	0.0490 (8)	0.9276 (9)
C17	0.4971 (4)	0.8206 (4)	0.1678 (4)	0.0609 (10)	0.9276 (9)
H17	0.571452	0.792859	0.203596	0.073*	0.9276 (9)
C18	0.4945 (5)	0.9129 (4)	0.1219 (4)	0.0722 (11)	0.9276 (9)
H18	0.567092	0.947097	0.124945	0.087*	0.9276 (9)
C19	0.3878 (4)	0.9554 (4)	0.0721 (4)	0.0695 (11)	0.9276 (9)
H19	0.386444	1.019893	0.042921	0.083*	0.9276 (9)
C20	0.2836 (4)	0.9034 (3)	0.0653 (4)	0.0623 (10)	0.9276 (9)
Cl1	0.14996 (15)	0.96107 (12)	0.00820 (17)	0.0925 (5)	0.9276 (9)
C21	0.2836 (4)	0.8100 (3)	0.1093 (3)	0.0511 (9)	0.9276 (9)
H21	0.210193	0.774962	0.103365	0.061*	0.9276 (9)
Mn1B	0.7452 (6)	0.3952 (5)	0.6294 (5)	0.0326 (14)	0.0724 (9)
O1B	0.6406 (18)	0.5042 (16)	0.6240 (17)	0.037 (3)	0.0724 (9)
N1B	0.672 (4)	0.586 (4)	0.727 (3)	0.033 (3)	0.0724 (9)
O2B	0.8412 (18)	0.4978 (17)	0.7831 (16)	0.036 (4)	0.0724 (9)
C1B	0.776 (2)	0.577 (2)	0.8025 (19)	0.034 (3)	0.0724 (9)
C2B	0.824 (3)	0.655 (3)	0.919 (2)	0.032 (3)	0.0724 (9)
C3B	0.935 (3)	0.635 (3)	0.995 (3)	0.034 (3)	0.0724 (9)
H3B	0.976449	0.576909	0.969409	0.041*	0.0724 (9)
C4B	0.984 (3)	0.699 (4)	1.106 (3)	0.036 (4)	0.0724 (9)
H4B	1.063759	0.691764	1.153958	0.043*	0.0724 (9)
C5B	0.918 (4)	0.773 (4)	1.149 (3)	0.040 (4)	0.0724 (9)
H5B	0.947815	0.807198	1.230734	0.048*	0.0724 (9)
C6B	0.810 (3)	0.797 (3)	1.076 (3)	0.036 (3)	0.0724 (9)
H6B	0.771559	0.857868	1.102185	0.044*	0.0724 (9)
C7B	0.757 (2)	0.732 (3)	0.962 (2)	0.032 (3)	0.0724 (9)
O3B	0.6462 (18)	0.7482 (19)	0.8949 (14)	0.038 (3)	0.0724 (9)
Mn2B	0.5604 (5)	0.7006 (5)	0.7338 (5)	0.0350 (13)	0.0724 (9)
O4B	0.4731 (17)	0.6419 (17)	0.5750 (13)	0.033 (3)	0.0724 (9)
N2B	0.362 (2)	0.683 (3)	0.538 (2)	0.030 (3)	0.0724 (9)
O5B	0.4275 (17)	0.7945 (18)	0.7235 (16)	0.035 (3)	0.0724 (9)
C8B	0.3431 (19)	0.754 (2)	0.6197 (19)	0.032 (3)	0.0724 (9)
C9B	0.225 (2)	0.797 (3)	0.588 (3)	0.035 (3)	0.0724 (9)
C10B	0.218 (3)	0.887 (4)	0.674 (3)	0.043 (3)	0.0724 (9)
H10B	0.288680	0.919778	0.740071	0.051*	0.0724 (9)
C11B	0.111 (4)	0.926 (4)	0.664 (4)	0.049 (4)	0.0724 (9)
H11B	0.101534	0.974617	0.728600	0.059*	0.0724 (9)
C12B	0.016 (4)	0.895 (4)	0.556 (4)	0.047 (4)	0.0724 (9)
H12B	−0.053806	0.934502	0.543132	0.056*	0.0724 (9)
C13B	0.020 (3)	0.808 (4)	0.469 (4)	0.040 (4)	0.0724 (9)
H13B	−0.048873	0.782822	0.398704	0.048*	0.0724 (9)
C14B	0.127 (3)	0.758 (3)	0.484 (2)	0.035 (3)	0.0724 (9)
O6B	0.129 (2)	0.677 (2)	0.3929 (19)	0.037 (3)	0.0724 (9)
O7B	0.490 (3)	0.696 (3)	0.318 (3)	0.051 (4)	0.0724 (9)
O8B	0.300 (2)	0.709 (2)	0.276 (2)	0.047 (4)	0.0724 (9)
C15B	0.390 (3)	0.696 (3)	0.241 (3)	0.047 (3)	0.0724 (9)
C16B	0.382 (3)	0.7623 (18)	0.1506 (19)	0.051 (3)	0.0724 (9)
C17B	0.470 (3)	0.780 (2)	0.108 (2)	0.054 (3)	0.0724 (9)
H17B	0.543708	0.748879	0.135972	0.065*	0.0724 (9)
C18B	0.452 (3)	0.842 (3)	0.025 (3)	0.060 (3)	0.0724 (9)
H18B	0.513237	0.853317	−0.003499	0.071*	0.0724 (9)
C19B	0.345 (3)	0.887 (2)	−0.016 (3)	0.065 (4)	0.0724 (9)
H19B	0.331301	0.929325	−0.072229	0.078*	0.0724 (9)
C20B	0.257 (2)	0.8705 (18)	0.025 (2)	0.070 (3)	0.0724 (9)
Cl1B	0.115 (2)	0.9218 (18)	−0.018 (3)	0.0925 (5)	0.0724 (9)
C21B	0.275 (3)	0.8083 (19)	0.108 (2)	0.056 (4)	0.0724 (9)
H21B	0.213524	0.797456	0.135697	0.067*	0.0724 (9)
O9	0.5419 (5)	0.8294 (3)	0.5924 (3)	0.0481 (10)	0.799 (3)
C22	0.6216 (4)	0.9054 (4)	0.6379 (4)	0.0489 (10)	0.799 (3)
H22	0.653529	0.928859	0.720848	0.059*	0.799 (3)
N3	0.6678 (6)	0.9585 (6)	0.5808 (6)	0.0517 (10)	0.799 (3)
C23	0.6266 (5)	0.9227 (5)	0.4543 (5)	0.0600 (12)	0.799 (3)
H23A	0.561726	0.861942	0.424876	0.090*	0.799 (3)
H23B	0.693378	0.896688	0.432353	0.090*	0.799 (3)
H23C	0.596737	0.985511	0.420197	0.090*	0.799 (3)
C24	0.7588 (6)	1.0515 (5)	0.6389 (6)	0.0757 (15)	0.799 (3)
H24A	0.821658	1.042275	0.606856	0.113*	0.799 (3)
H24B	0.793968	1.055163	0.722846	0.113*	0.799 (3)
H24C	0.722857	1.120618	0.626793	0.113*	0.799 (3)
O9B	0.660 (3)	0.834 (2)	0.700 (2)	0.049 (4)	0.0724 (9)
C22B	0.632 (5)	0.861 (4)	0.609 (3)	0.052 (3)	0.0724 (9)
H22B	0.571330	0.812729	0.542712	0.062*	0.0724 (9)
N3B	0.677 (6)	0.951 (7)	0.590 (5)	0.058 (3)	0.0724 (9)
C23B	0.796 (4)	1.006 (5)	0.676 (4)	0.064 (4)	0.0724 (9)
H23D	0.818178	0.975947	0.745556	0.097*	0.0724 (9)
H23E	0.791073	1.086409	0.698493	0.097*	0.0724 (9)
H23F	0.856079	0.992314	0.641292	0.097*	0.0724 (9)
C24B	0.642 (6)	0.973 (5)	0.477 (4)	0.060 (3)	0.0724 (9)
H24D	0.588667	0.910268	0.418078	0.089*	0.0724 (9)
H24E	0.713852	0.984754	0.459956	0.089*	0.0724 (9)
H24F	0.600370	1.040785	0.476374	0.089*	0.0724 (9)
O9C	0.548 (4)	0.856 (3)	0.622 (2)	0.047 (3)	0.129 (3)
C22C	0.565 (2)	0.8569 (19)	0.534 (2)	0.052 (3)	0.129 (3)
H22C	0.528704	0.794608	0.469842	0.063*	0.129 (3)
N3C	0.629 (3)	0.9370 (19)	0.518 (3)	0.055 (3)	0.129 (3)
C23C	0.635 (3)	0.924 (3)	0.401 (2)	0.061 (4)	0.129 (3)
H23G	0.631105	0.844936	0.364179	0.091*	0.129 (3)
H23H	0.710969	0.962629	0.409399	0.091*	0.129 (3)
H23I	0.567681	0.956855	0.351625	0.091*	0.129 (3)
C24C	0.682 (3)	1.042 (2)	0.598 (3)	0.063 (3)	0.129 (3)
H24G	0.769182	1.046028	0.621857	0.094*	0.129 (3)
H24H	0.660782	1.049299	0.666440	0.094*	0.129 (3)
H24I	0.651117	1.101618	0.559181	0.094*	0.129 (3)
O10	0.3646 (15)	0.5947 (13)	0.7207 (11)	0.050 (2)	0.498 (3)
C25	0.3844 (9)	0.5822 (9)	0.8165 (9)	0.0508 (17)	0.498 (3)
H25	0.444775	0.633788	0.878917	0.061*	0.498 (3)
N4	0.3337 (8)	0.5069 (8)	0.8461 (7)	0.0573 (16)	0.498 (3)
C26	0.2464 (14)	0.4197 (11)	0.7651 (10)	0.061 (2)	0.498 (3)
H26A	0.239360	0.419362	0.685592	0.074*	0.498 (3)
H26B	0.168689	0.431241	0.771828	0.074*	0.498 (3)
H26C	0.270784	0.348077	0.781915	0.074*	0.498 (3)
C27	0.3757 (10)	0.4926 (8)	0.9663 (7)	0.078 (2)	0.498 (3)
H27A	0.307397	0.490705	0.990550	0.118*	0.498 (3)
H27B	0.435119	0.555054	1.019088	0.118*	0.498 (3)
H27C	0.412480	0.422581	0.969352	0.118*	0.498 (3)
O10B	0.430 (3)	0.565 (3)	0.746 (3)	0.052 (3)	0.0724 (9)
C25B	0.403 (3)	0.574 (4)	0.829 (3)	0.054 (3)	0.0724 (9)
H25B	0.425482	0.645023	0.882924	0.064*	0.0724 (9)
N4B	0.346 (3)	0.500 (4)	0.855 (3)	0.061 (3)	0.0724 (9)
C26B	0.304 (6)	0.389 (4)	0.786 (4)	0.060 (4)	0.0724 (9)
H26D	0.268902	0.389297	0.702961	0.072*	0.0724 (9)
H26E	0.243759	0.357363	0.808227	0.072*	0.0724 (9)
H26F	0.371986	0.343190	0.798921	0.072*	0.0724 (9)
C27B	0.320 (6)	0.525 (5)	0.961 (4)	0.064 (4)	0.0724 (9)
H27D	0.376487	0.586970	1.018656	0.096*	0.0724 (9)
H27E	0.327125	0.459207	0.992693	0.096*	0.0724 (9)
H27F	0.237628	0.546113	0.941661	0.096*	0.0724 (9)
O10C	0.3646 (16)	0.5782 (15)	0.7088 (13)	0.044 (2)	0.430 (3)
C25C	0.3444 (9)	0.5894 (10)	0.7974 (11)	0.055 (2)	0.430 (3)
H25C	0.372376	0.658121	0.854651	0.066*	0.430 (3)
N4C	0.2856 (7)	0.5110 (8)	0.8186 (8)	0.0576 (18)	0.430 (3)
C26C	0.2425 (17)	0.4073 (12)	0.7344 (10)	0.057 (2)	0.430 (3)
H26G	0.211898	0.420372	0.655676	0.068*	0.430 (3)
H26H	0.177617	0.370439	0.747059	0.068*	0.430 (3)
H26I	0.308084	0.359783	0.742299	0.068*	0.430 (3)
C27C	0.2624 (11)	0.5265 (10)	0.9256 (8)	0.078 (2)	0.430 (3)
H27G	0.176493	0.531502	0.907383	0.117*	0.430 (3)
H27H	0.308219	0.595349	0.981014	0.117*	0.430 (3)
H27I	0.287025	0.463077	0.960265	0.117*	0.430 (3)
O11	0.7624 (12)	0.6718 (11)	0.5247 (13)	0.070 (2)	0.516 (5)
C28	0.8618 (9)	0.6462 (9)	0.5798 (8)	0.081 (2)	0.516 (5)
H28	0.888305	0.581081	0.544658	0.098*	0.516 (5)
N5	0.9360 (7)	0.7053 (7)	0.6879 (6)	0.0660 (17)	0.516 (5)
C29	0.9012 (9)	0.7930 (8)	0.7480 (8)	0.085 (2)	0.516 (5)
H29A	0.962890	0.818732	0.826930	0.102*	0.516 (5)
H29B	0.825322	0.770472	0.753081	0.102*	0.516 (5)
H29C	0.889915	0.853440	0.707767	0.102*	0.516 (5)
C30	1.0409 (11)	0.7016 (12)	0.7730 (12)	0.111 (3)	0.516 (5)
H30A	1.049979	0.759235	0.843300	0.133*	0.516 (5)
H30B	1.106599	0.714629	0.747737	0.133*	0.516 (5)
H30C	1.043252	0.628128	0.790383	0.133*	0.516 (5)
O11B	0.7653 (12)	0.6424 (12)	0.5394 (12)	0.071 (3)	0.484 (5)
C28B	0.8447 (8)	0.6931 (9)	0.6316 (8)	0.0738 (19)	0.484 (5)
H28B	0.833496	0.757837	0.681652	0.089*	0.484 (5)
N5B	0.9554 (8)	0.6444 (9)	0.6556 (9)	0.085 (2)	0.484 (5)
C29B	0.9812 (11)	0.5603 (10)	0.5813 (10)	0.101 (3)	0.484 (5)
H29D	0.998150	0.495775	0.615537	0.121*	0.484 (5)
H29E	1.051586	0.585401	0.568425	0.121*	0.484 (5)
H29F	0.912325	0.539235	0.506523	0.121*	0.484 (5)
C30B	1.0546 (10)	0.6572 (10)	0.7364 (10)	0.083 (2)	0.484 (5)
H30D	1.109659	0.611626	0.709900	0.100*	0.484 (5)
H30E	1.046275	0.634324	0.802484	0.100*	0.484 (5)
H30F	1.086556	0.735893	0.760885	0.100*	0.484 (5)

### Bis(µ-3-chlorobenzoato)hexakis(dimethylformamide)tetrakis(µ4-N,2-dioxidobenzene-1-carboximidato)tetramanganese(III)disodium(I) (1) Atomic displacement parameters (Å^2^)

**Table:**

	*U*^11^	*U*^22^	*U*^33^	*U*^12^	*U*^13^	*U*^23^
Na1	0.0352 (6)	0.0476 (6)	0.0379 (6)	−0.0037 (5)	0.0062 (5)	0.0175 (5)
Mn1	0.0276 (2)	0.0299 (2)	0.0214 (2)	−0.00225 (18)	0.00067 (18)	0.00714 (17)
O1	0.0329 (11)	0.0366 (10)	0.0249 (9)	0.0039 (8)	0.0045 (8)	0.0014 (8)
N1	0.0294 (13)	0.0317 (14)	0.0247 (13)	0.0010 (11)	0.0054 (10)	0.0048 (10)
O2	0.0320 (12)	0.0387 (11)	0.0277 (10)	0.0003 (9)	0.0011 (9)	0.0058 (8)
C1	0.0313 (14)	0.0330 (13)	0.0267 (12)	−0.0014 (11)	0.0049 (11)	0.0118 (10)
C2	0.0333 (16)	0.0316 (13)	0.0340 (15)	−0.0014 (12)	0.0096 (12)	0.0109 (12)
C3	0.0362 (18)	0.0388 (17)	0.0414 (15)	0.0029 (13)	0.0102 (14)	0.0093 (13)
C4	0.0359 (18)	0.046 (2)	0.057 (2)	0.0055 (15)	0.0095 (16)	0.0043 (16)
C5	0.040 (2)	0.0463 (18)	0.066 (2)	0.0065 (17)	0.0161 (18)	−0.0051 (18)
C6	0.0374 (19)	0.0424 (18)	0.0506 (18)	0.0011 (15)	0.0107 (16)	−0.0041 (15)
C7	0.0334 (15)	0.0308 (14)	0.0375 (15)	0.0004 (12)	0.0106 (13)	0.0081 (12)
O3	0.0346 (11)	0.0403 (11)	0.0347 (11)	0.0007 (9)	0.0042 (9)	−0.0004 (9)
Mn2	0.0302 (3)	0.0294 (2)	0.0228 (2)	−0.00059 (18)	0.00305 (18)	0.00582 (17)
O4	0.0301 (10)	0.0379 (10)	0.0232 (9)	0.0036 (8)	−0.0044 (8)	0.0035 (8)
N2	0.0299 (14)	0.0313 (16)	0.0221 (11)	−0.0011 (10)	−0.0012 (10)	0.0066 (9)
O5	0.0354 (11)	0.0318 (10)	0.0268 (10)	−0.0016 (8)	0.0036 (9)	0.0052 (8)
C8	0.0324 (14)	0.0261 (12)	0.0225 (11)	−0.0014 (10)	0.0050 (10)	0.0086 (9)
C9	0.0330 (15)	0.0289 (14)	0.0252 (13)	−0.0055 (11)	0.0026 (12)	0.0095 (11)
C10	0.0452 (18)	0.0338 (15)	0.0247 (15)	−0.0036 (13)	0.0028 (13)	0.0078 (12)
C11	0.054 (2)	0.0376 (17)	0.0245 (15)	−0.0037 (15)	0.0004 (15)	0.0060 (12)
C12	0.0431 (18)	0.0389 (19)	0.0249 (15)	−0.0089 (15)	−0.0050 (14)	0.0074 (12)
C13	0.0368 (17)	0.0365 (15)	0.0255 (15)	−0.0095 (13)	−0.0036 (12)	0.0108 (13)
C14	0.0335 (16)	0.0305 (13)	0.0234 (13)	−0.0082 (11)	−0.0028 (12)	0.0100 (11)
O6	0.0314 (11)	0.0370 (10)	0.0260 (10)	−0.0012 (8)	−0.0001 (8)	0.0060 (8)
O7	0.0480 (14)	0.0542 (15)	0.0490 (14)	0.0048 (12)	0.0149 (12)	0.0253 (12)
O8	0.0453 (13)	0.0394 (11)	0.0360 (11)	−0.0052 (10)	0.0067 (10)	0.0172 (9)
C15	0.0537 (19)	0.0391 (16)	0.0358 (16)	0.0008 (15)	0.0160 (14)	0.0133 (13)
C16	0.056 (2)	0.0463 (17)	0.0471 (17)	−0.0025 (15)	0.0187 (15)	0.0193 (14)
C17	0.064 (2)	0.062 (2)	0.062 (2)	−0.0036 (18)	0.0241 (19)	0.0258 (18)
C18	0.080 (3)	0.069 (2)	0.076 (3)	−0.012 (2)	0.032 (2)	0.031 (2)
C19	0.090 (3)	0.055 (2)	0.076 (2)	−0.002 (2)	0.036 (2)	0.0341 (19)
C20	0.075 (2)	0.0500 (19)	0.067 (2)	0.0059 (18)	0.0235 (19)	0.0303 (17)
Cl1	0.0912 (11)	0.0753 (9)	0.1192 (12)	0.0196 (7)	0.0257 (9)	0.0640 (10)
C21	0.058 (2)	0.0462 (17)	0.0495 (18)	−0.0017 (16)	0.0162 (16)	0.0212 (15)
Mn1B	0.035 (3)	0.030 (3)	0.026 (3)	0.005 (2)	0.004 (2)	0.008 (2)
O1B	0.032 (5)	0.035 (5)	0.028 (5)	−0.003 (5)	−0.004 (5)	0.005 (5)
N1B	0.031 (5)	0.032 (5)	0.024 (4)	0.000 (4)	−0.002 (4)	0.007 (4)
O2B	0.030 (6)	0.038 (6)	0.027 (6)	0.003 (5)	−0.004 (5)	0.006 (5)
C1B	0.034 (4)	0.032 (4)	0.024 (4)	−0.002 (4)	−0.001 (4)	0.006 (4)
C2B	0.032 (4)	0.032 (4)	0.022 (4)	−0.004 (4)	−0.001 (4)	0.008 (4)
C3B	0.036 (5)	0.034 (4)	0.023 (5)	−0.005 (4)	−0.002 (4)	0.008 (4)
C4B	0.041 (5)	0.034 (5)	0.023 (5)	−0.002 (5)	−0.001 (5)	0.009 (5)
C5B	0.045 (5)	0.037 (5)	0.025 (5)	−0.004 (5)	−0.002 (5)	0.007 (5)
C6B	0.041 (5)	0.035 (5)	0.024 (5)	−0.004 (5)	0.002 (5)	0.007 (5)
C7B	0.034 (4)	0.029 (4)	0.024 (4)	−0.002 (4)	0.002 (4)	0.008 (4)
O3B	0.038 (4)	0.035 (4)	0.030 (4)	0.000 (4)	0.002 (4)	0.006 (4)
Mn2B	0.033 (2)	0.034 (2)	0.030 (2)	0.005 (2)	0.004 (2)	0.006 (2)
O4B	0.030 (4)	0.032 (4)	0.027 (4)	0.007 (4)	0.003 (4)	0.002 (4)
N2B	0.029 (4)	0.032 (4)	0.025 (4)	0.002 (4)	0.007 (4)	0.005 (4)
O5B	0.036 (5)	0.032 (5)	0.025 (4)	0.005 (4)	0.009 (4)	−0.008 (4)
C8B	0.030 (4)	0.031 (4)	0.029 (4)	0.000 (4)	0.007 (4)	0.006 (4)
C9B	0.032 (4)	0.034 (4)	0.035 (4)	0.001 (4)	0.009 (4)	0.006 (4)
C10B	0.035 (5)	0.039 (5)	0.046 (5)	0.003 (5)	0.011 (5)	−0.001 (4)
C11B	0.037 (5)	0.041 (5)	0.054 (5)	0.005 (5)	0.009 (5)	−0.003 (5)
C12B	0.037 (5)	0.041 (5)	0.052 (5)	0.005 (5)	0.011 (5)	0.002 (5)
C13B	0.034 (5)	0.037 (5)	0.043 (5)	0.003 (5)	0.010 (5)	0.008 (5)
C14B	0.033 (4)	0.034 (4)	0.035 (4)	0.000 (4)	0.010 (4)	0.010 (4)
O6B	0.036 (5)	0.036 (5)	0.033 (5)	0.002 (5)	0.005 (4)	0.009 (4)
O7B	0.055 (6)	0.048 (6)	0.047 (6)	0.000 (6)	0.012 (6)	0.021 (6)
O8B	0.053 (6)	0.042 (6)	0.044 (6)	0.001 (6)	0.012 (6)	0.018 (5)
C15B	0.053 (4)	0.046 (4)	0.044 (4)	−0.001 (4)	0.016 (4)	0.021 (4)
C16B	0.059 (4)	0.049 (4)	0.051 (4)	−0.001 (4)	0.020 (4)	0.022 (4)
C17B	0.063 (5)	0.055 (5)	0.057 (5)	−0.005 (5)	0.029 (5)	0.028 (5)
C18B	0.072 (5)	0.058 (5)	0.065 (5)	−0.007 (5)	0.035 (5)	0.032 (5)
C19B	0.079 (5)	0.057 (5)	0.071 (5)	−0.003 (5)	0.032 (5)	0.034 (5)
C20B	0.079 (4)	0.059 (4)	0.081 (4)	0.004 (4)	0.026 (4)	0.040 (4)
Cl1B	0.0912 (11)	0.0753 (9)	0.1192 (12)	0.0196 (7)	0.0257 (9)	0.0640 (10)
C21B	0.065 (5)	0.050 (5)	0.058 (5)	0.001 (5)	0.021 (5)	0.026 (5)
O9	0.0485 (17)	0.051 (2)	0.044 (2)	−0.0092 (17)	0.0126 (18)	0.0199 (15)
C22	0.051 (2)	0.049 (2)	0.046 (2)	−0.0063 (19)	0.0210 (17)	0.0076 (17)
N3	0.053 (2)	0.050 (2)	0.056 (2)	−0.0056 (17)	0.0258 (17)	0.0119 (16)
C23	0.062 (3)	0.063 (2)	0.057 (3)	−0.004 (2)	0.019 (2)	0.024 (2)
C24	0.071 (3)	0.069 (3)	0.083 (3)	−0.019 (3)	0.033 (3)	0.006 (3)
O9B	0.050 (5)	0.046 (5)	0.048 (5)	−0.002 (5)	0.015 (5)	0.014 (5)
C22B	0.053 (4)	0.051 (4)	0.051 (4)	−0.005 (4)	0.019 (4)	0.014 (4)
N3B	0.057 (4)	0.057 (4)	0.059 (4)	−0.008 (4)	0.024 (4)	0.013 (4)
C23B	0.061 (6)	0.062 (6)	0.067 (6)	−0.010 (6)	0.025 (6)	0.010 (6)
C24B	0.060 (5)	0.058 (5)	0.060 (5)	−0.005 (5)	0.021 (5)	0.015 (5)
O9C	0.051 (5)	0.044 (5)	0.046 (5)	−0.001 (5)	0.015 (5)	0.018 (5)
C22C	0.054 (4)	0.051 (4)	0.053 (4)	−0.005 (4)	0.020 (4)	0.015 (4)
N3C	0.057 (4)	0.055 (4)	0.058 (4)	−0.007 (4)	0.025 (4)	0.015 (4)
C23C	0.063 (6)	0.059 (6)	0.061 (6)	0.000 (6)	0.023 (6)	0.020 (6)
C24C	0.060 (4)	0.062 (4)	0.063 (4)	−0.007 (4)	0.022 (4)	0.013 (4)
O10	0.052 (3)	0.047 (4)	0.045 (4)	−0.013 (3)	0.008 (3)	0.017 (3)
C25	0.067 (4)	0.047 (3)	0.041 (3)	−0.011 (3)	0.025 (3)	0.010 (2)
N4	0.082 (4)	0.049 (2)	0.050 (3)	−0.012 (3)	0.035 (3)	0.012 (2)
C26	0.075 (4)	0.057 (4)	0.058 (5)	−0.011 (3)	0.034 (4)	0.011 (4)
C27	0.111 (5)	0.072 (4)	0.053 (3)	−0.022 (4)	0.036 (4)	0.012 (3)
O10B	0.063 (5)	0.049 (5)	0.042 (5)	−0.012 (5)	0.019 (5)	0.010 (5)
C25B	0.068 (4)	0.049 (4)	0.043 (4)	−0.012 (4)	0.022 (4)	0.010 (4)
N4B	0.082 (4)	0.055 (4)	0.048 (4)	−0.016 (4)	0.032 (4)	0.009 (4)
C26B	0.080 (6)	0.057 (5)	0.049 (5)	−0.015 (5)	0.034 (5)	0.009 (5)
C27B	0.090 (6)	0.058 (5)	0.048 (5)	−0.016 (5)	0.034 (5)	0.010 (5)
O10C	0.050 (3)	0.044 (4)	0.036 (3)	−0.008 (3)	0.015 (3)	0.014 (3)
C25C	0.069 (4)	0.049 (3)	0.044 (4)	−0.015 (3)	0.019 (3)	0.009 (3)
N4C	0.080 (4)	0.053 (3)	0.042 (3)	−0.018 (3)	0.029 (3)	0.007 (3)
C26C	0.077 (4)	0.058 (4)	0.041 (4)	−0.019 (4)	0.036 (4)	0.002 (3)
C27C	0.110 (5)	0.069 (4)	0.054 (4)	−0.025 (4)	0.038 (4)	0.003 (3)
O11	0.061 (3)	0.075 (5)	0.073 (4)	−0.030 (3)	0.024 (3)	0.020 (3)
C28	0.083 (4)	0.086 (4)	0.078 (4)	−0.026 (4)	0.033 (3)	0.024 (3)
N5	0.068 (3)	0.075 (4)	0.058 (3)	−0.018 (3)	0.025 (3)	0.025 (3)
C29	0.087 (5)	0.091 (5)	0.081 (5)	−0.015 (4)	0.034 (4)	0.028 (4)
C30	0.098 (5)	0.114 (6)	0.110 (6)	−0.022 (5)	0.025 (5)	0.035 (5)
O11B	0.059 (4)	0.089 (6)	0.060 (4)	−0.041 (4)	0.023 (3)	0.008 (4)
C28B	0.081 (4)	0.083 (4)	0.063 (4)	−0.031 (4)	0.037 (3)	0.014 (3)
N5B	0.085 (4)	0.088 (5)	0.084 (4)	−0.020 (4)	0.029 (3)	0.035 (4)
C29B	0.100 (5)	0.103 (6)	0.114 (6)	−0.006 (5)	0.047 (5)	0.043 (5)
C30B	0.078 (4)	0.086 (5)	0.104 (5)	0.011 (4)	0.046 (4)	0.042 (4)

### Bis(µ-3-chlorobenzoato)hexakis(dimethylformamide)tetrakis(µ4-N,2-dioxidobenzene-1-carboximidato)tetramanganese(III)disodium(I) (1) Geometric parameters (Å, º)

**Table:**

Na1—O11B	2.272 (12)	C13B—C14B	1.409 (19)
Na1—O7	2.347 (3)	C13B—H13B	0.9500
Na1—O11	2.357 (12)	C14B—O6B	1.351 (18)
Na1—O7B	2.38 (3)	O7B—C15B	1.241 (19)
Na1—O4B	2.430 (19)	O8B—C15B	1.307 (19)
Na1—O1	2.431 (2)	C15B—C16B	1.525 (18)
Na1—O1B^i^	2.46 (2)	C16B—C21B	1.381 (18)
Na1—O4^i^	2.465 (2)	C16B—C17B	1.392 (19)
Na1—O4	2.498 (2)	C17B—C18B	1.395 (19)
Na1—O1B	2.51 (2)	C17B—H17B	0.9500
Na1—O10B^i^	2.57 (3)	C18B—C19B	1.38 (2)
Na1—O1^i^	2.585 (2)	C18B—H18B	0.9500
Mn1—O6^i^	1.852 (2)	C19B—C20B	1.367 (19)
Mn1—O1	1.873 (2)	C19B—H19B	0.9500
Mn1—O2	1.960 (2)	C20B—C21B	1.392 (19)
Mn1—N2^i^	1.974 (3)	C20B—Cl1B	1.760 (18)
Mn1—O8	2.063 (2)	C21B—H21B	0.9500
O1—N1	1.389 (3)	O9—C22	1.211 (6)
N1—C1	1.299 (4)	C22—N3	1.329 (6)
N1—Mn2	1.973 (2)	C22—H22	0.9500
O2—C1	1.306 (3)	N3—C24	1.437 (7)
C1—C2	1.465 (4)	N3—C23	1.453 (8)
C2—C3	1.400 (5)	C23—H23A	0.9800
C2—C7	1.414 (4)	C23—H23B	0.9800
C3—C4	1.370 (5)	C23—H23C	0.9800
C3—H3	0.9500	C24—H24A	0.9800
C4—C5	1.383 (5)	C24—H24B	0.9800
C4—H4	0.9500	C24—H24C	0.9800
C5—C6	1.384 (5)	O9B—C22B	1.20 (2)
C5—H5	0.9500	C22B—N3B	1.34 (2)
C6—C7	1.392 (5)	C22B—H22B	0.9500
C6—H6	0.9500	N3B—C24B	1.43 (2)
C7—O3	1.328 (4)	N3B—C23B	1.48 (2)
O3—Mn2	1.859 (2)	C23B—H23D	0.9800
Mn2—O4	1.879 (2)	C23B—H23E	0.9800
Mn2—O5	1.955 (2)	C23B—H23F	0.9800
Mn2—O9	2.236 (4)	C24B—H24D	0.9800
Mn2—O10	2.261 (15)	C24B—H24E	0.9800
O4—N2	1.394 (3)	C24B—H24F	0.9800
N2—C8	1.305 (4)	O9C—C22C	1.208 (18)
O5—C8	1.311 (3)	C22C—N3C	1.348 (17)
C8—C9	1.467 (4)	C22C—H22C	0.9500
C9—C14	1.401 (4)	N3C—C24C	1.419 (19)
C9—C10	1.406 (4)	N3C—C23C	1.49 (2)
C10—C11	1.378 (5)	C23C—H23G	0.9800
C10—H10	0.9500	C23C—H23H	0.9800
C11—C12	1.392 (5)	C23C—H23I	0.9800
C11—H11	0.9500	C24C—H24G	0.9800
C12—C13	1.385 (5)	C24C—H24H	0.9800
C12—H12	0.9500	C24C—H24I	0.9800
C13—C14	1.405 (4)	O10—C25	1.196 (10)
C13—H13	0.9500	C25—N4	1.301 (9)
C14—O6	1.339 (4)	C25—H25	0.9500
O7—C15	1.232 (4)	N4—C26	1.417 (11)
O8—C15	1.274 (4)	N4—C27	1.472 (9)
C15—C16	1.518 (4)	C26—H26A	0.9800
C16—C21	1.380 (5)	C26—H26B	0.9800
C16—C17	1.394 (6)	C26—H26C	0.9800
C17—C18	1.389 (6)	C27—H27A	0.9800
C17—H17	0.9500	C27—H27B	0.9800
C18—C19	1.376 (7)	C27—H27C	0.9800
C18—H18	0.9500	O10B—C25B	1.197 (18)
C19—C20	1.367 (6)	C25B—N4B	1.305 (17)
C19—H19	0.9500	C25B—H25B	0.9500
C20—C21	1.390 (5)	N4B—C26B	1.426 (18)
C20—Cl1	1.739 (4)	N4B—C27B	1.461 (17)
C21—H21	0.9500	C26B—H26D	0.9800
Mn1B—O6B^i^	1.85 (3)	C26B—H26E	0.9800
Mn1B—O1B	1.852 (15)	C26B—H26F	0.9800
Mn1B—O2B	1.988 (16)	C27B—H27D	0.9800
Mn1B—N2B^i^	2.04 (2)	C27B—H27E	0.9800
Mn1B—O8B^i^	2.13 (3)	C27B—H27F	0.9800
O1B—N1B	1.378 (18)	O10C—C25C	1.214 (12)
N1B—C1B	1.290 (18)	C25C—N4C	1.324 (10)
N1B—Mn2B	1.981 (15)	C25C—H25C	0.9500
O2B—C1B	1.299 (17)	N4C—C26C	1.422 (11)
C1B—C2B	1.472 (17)	N4C—C27C	1.456 (10)
C2B—C3B	1.394 (19)	C26C—H26G	0.9800
C2B—C7B	1.395 (18)	C26C—H26H	0.9800
C3B—C4B	1.35 (2)	C26C—H26I	0.9800
C3B—H3B	0.9500	C27C—H27G	0.9800
C4B—C5B	1.37 (2)	C27C—H27H	0.9800
C4B—H4B	0.9500	C27C—H27I	0.9800
C5B—C6B	1.38 (2)	O11—C28	1.230 (14)
C5B—H5B	0.9500	C28—N5	1.359 (10)
C6B—C7B	1.393 (19)	C28—H28	0.9500
C6B—H6B	0.9500	N5—C30	1.335 (11)
C7B—O3B	1.342 (18)	N5—C29	1.347 (11)
O3B—Mn2B	1.867 (15)	C29—H29A	0.9800
Mn2B—O4B	1.860 (15)	C29—H29B	0.9800
Mn2B—O5B	1.971 (15)	C29—H29C	0.9800
Mn2B—O9B	2.233 (18)	C30—H30A	0.9800
Mn2B—O10B	2.35 (3)	C30—H30B	0.9800
O4B—N2B	1.388 (18)	C30—H30C	0.9800
N2B—C8B	1.291 (18)	O11B—C28B	1.229 (13)
O5B—C8B	1.314 (18)	C28B—N5B	1.427 (12)
C8B—C9B	1.470 (17)	C28B—H28B	0.9500
C9B—C14B	1.388 (18)	N5B—C30B	1.234 (11)
C9B—C10B	1.402 (19)	N5B—C29B	1.362 (12)
C10B—C11B	1.36 (2)	C29B—H29D	0.9800
C10B—H10B	0.9500	C29B—H29E	0.9800
C11B—C12B	1.39 (2)	C29B—H29F	0.9800
C11B—H11B	0.9500	C30B—H30D	0.9800
C12B—C13B	1.38 (2)	C30B—H30E	0.9800
C12B—H12B	0.9500	C30B—H30F	0.9800
			
O7—Na1—O11	98.5 (3)	Na1—O4B—Na1^i^	83.3 (5)
O11B—Na1—O7B	103.5 (8)	C8B—N2B—O4B	113.9 (17)
O11B—Na1—O4B	104.1 (7)	C8B—O5B—Mn2B	106.1 (12)
O7B—Na1—O4B	95.0 (10)	N2B—C8B—O5B	122.5 (17)
O7—Na1—O1	84.47 (9)	N2B—C8B—C9B	116.9 (17)
O11—Na1—O1	143.6 (5)	O5B—C8B—C9B	120.3 (17)
O7—Na1—O1B^i^	82.6 (5)	C14B—C9B—C10B	120.7 (19)
O11—Na1—O1B^i^	169.1 (6)	C14B—C9B—C8B	126 (2)
O1—Na1—O1B^i^	25.5 (4)	C10B—C9B—C8B	113 (2)
O7—Na1—O4^i^	100.06 (9)	C11B—C10B—C9B	120 (2)
O11—Na1—O4^i^	148.2 (4)	C11B—C10B—H10B	120.0
O1—Na1—O4^i^	64.14 (7)	C9B—C10B—H10B	120.0
O7—Na1—O4	134.56 (10)	C10B—C11B—C12B	119 (2)
O11—Na1—O4	90.7 (4)	C10B—C11B—H11B	120.5
O1—Na1—O4	63.73 (7)	C12B—C11B—H11B	120.5
O4^i^—Na1—O4	94.37 (7)	C13B—C12B—C11B	121 (2)
O11B—Na1—O1B	73.7 (6)	C13B—C12B—H12B	119.6
O7B—Na1—O1B	157.2 (10)	C11B—C12B—H12B	119.6
O4B—Na1—O1B	64.6 (6)	C12B—C13B—C14B	120 (2)
O1B^i^—Na1—O1B	94.6 (5)	C12B—C13B—H13B	120.1
O7—Na1—O10B^i^	68.1 (8)	C14B—C13B—H13B	120.1
O11—Na1—O10B^i^	104.5 (9)	O6B—C14B—C9B	125 (2)
O1—Na1—O10B^i^	110.1 (8)	O6B—C14B—C13B	117 (2)
O4^i^—Na1—O10B^i^	60.0 (7)	C9B—C14B—C13B	118.1 (19)
O4—Na1—O10B^i^	151.2 (7)	C15B—O7B—Na1	133 (2)
O7—Na1—O1^i^	159.24 (10)	O7B—C15B—O8B	116 (3)
O11—Na1—O1^i^	93.7 (3)	O7B—C15B—C16B	118 (2)
O1—Na1—O1^i^	95.56 (7)	O8B—C15B—C16B	112 (2)
O4^i^—Na1—O1^i^	61.99 (7)	C21B—C16B—C17B	117.9 (19)
O4—Na1—O1^i^	61.49 (7)	C21B—C16B—C15B	115 (2)
O6^i^—Mn1—O1	166.65 (9)	C17B—C16B—C15B	127 (2)
O6^i^—Mn1—O2	97.56 (9)	C16B—C17B—C18B	122 (2)
O1—Mn1—O2	81.08 (8)	C16B—C17B—H17B	119.2
O6^i^—Mn1—N2^i^	88.87 (10)	C18B—C17B—H17B	119.2
O1—Mn1—N2^i^	87.74 (10)	C19B—C18B—C17B	119 (2)
O2—Mn1—N2^i^	157.65 (14)	C19B—C18B—H18B	120.6
O6^i^—Mn1—O8	93.89 (9)	C17B—C18B—H18B	120.6
O1—Mn1—O8	99.46 (9)	C20B—C19B—C18B	120 (2)
O2—Mn1—O8	95.44 (9)	C20B—C19B—H19B	119.8
N2^i^—Mn1—O8	105.50 (15)	C18B—C19B—H19B	119.8
O6^i^—Mn1—Na1	146.74 (7)	C19B—C20B—C21B	120 (2)
O1—Mn1—Na1	38.59 (7)	C19B—C20B—Cl1B	126 (2)
O2—Mn1—Na1	115.38 (6)	C21B—C20B—Cl1B	114.1 (19)
N2^i^—Mn1—Na1	62.51 (10)	C16B—C21B—C20B	121 (2)
O8—Mn1—Na1	79.18 (7)	C16B—C21B—H21B	119.6
N1—O1—Mn1	114.10 (16)	C20B—C21B—H21B	119.6
N1—O1—Na1	122.13 (17)	C22—O9—Mn2	132.4 (3)
Mn1—O1—Na1	112.69 (9)	O9—C22—N3	125.0 (5)
N1—O1—Na1^i^	104.77 (17)	O9—C22—H22	117.5
Mn1—O1—Na1^i^	114.52 (10)	N3—C22—H22	117.5
Na1—O1—Na1^i^	84.44 (7)	C22—N3—C24	122.8 (6)
C1—N1—O1	113.2 (2)	C22—N3—C23	119.9 (6)
C1—N1—Mn2	130.7 (2)	C24—N3—C23	117.3 (5)
O1—N1—Mn2	115.42 (18)	N3—C23—H23A	109.5
C1—O2—Mn1	111.39 (18)	N3—C23—H23B	109.5
N1—C1—O2	119.4 (3)	H23A—C23—H23B	109.5
N1—C1—C2	120.4 (3)	N3—C23—H23C	109.5
O2—C1—C2	120.2 (3)	H23A—C23—H23C	109.5
C3—C2—C7	119.0 (3)	H23B—C23—H23C	109.5
C3—C2—C1	117.8 (3)	N3—C24—H24A	109.5
C7—C2—C1	123.1 (3)	N3—C24—H24B	109.5
C4—C3—C2	122.1 (3)	H24A—C24—H24B	109.5
C4—C3—H3	118.9	N3—C24—H24C	109.5
C2—C3—H3	118.9	H24A—C24—H24C	109.5
C3—C4—C5	118.8 (3)	H24B—C24—H24C	109.5
C3—C4—H4	120.6	C22B—O9B—Mn2B	126 (2)
C5—C4—H4	120.6	O9B—C22B—N3B	127 (3)
C4—C5—C6	120.5 (3)	O9B—C22B—H22B	116.5
C4—C5—H5	119.7	N3B—C22B—H22B	116.5
C6—C5—H5	119.7	C22B—N3B—C24B	123 (4)
C5—C6—C7	121.5 (3)	C22B—N3B—C23B	118 (3)
C5—C6—H6	119.2	C24B—N3B—C23B	115 (3)
C7—C6—H6	119.2	N3B—C23B—H23D	109.5
O3—C7—C6	117.7 (3)	N3B—C23B—H23E	109.5
O3—C7—C2	124.3 (3)	H23D—C23B—H23E	109.5
C6—C7—C2	118.0 (3)	N3B—C23B—H23F	109.5
C7—O3—Mn2	130.2 (2)	H23D—C23B—H23F	109.5
O3—Mn2—O4	177.31 (10)	H23E—C23B—H23F	109.5
O3—Mn2—O5	99.21 (9)	N3B—C24B—H24D	109.5
O4—Mn2—O5	81.99 (8)	N3B—C24B—H24E	109.5
O3—Mn2—N1	90.03 (10)	H24D—C24B—H24E	109.5
O4—Mn2—N1	88.64 (9)	N3B—C24B—H24F	109.5
O5—Mn2—N1	170.20 (10)	H24D—C24B—H24F	109.5
O3—Mn2—O9	94.22 (15)	H24E—C24B—H24F	109.5
O4—Mn2—O9	88.08 (15)	O9C—C22C—N3C	127 (2)
O5—Mn2—O9	94.23 (13)	O9C—C22C—H22C	116.6
N1—Mn2—O9	88.30 (14)	N3C—C22C—H22C	116.6
O3—Mn2—O10	90.2 (4)	C22C—N3C—C24C	127 (2)
O4—Mn2—O10	87.3 (4)	C22C—N3C—C23C	118 (2)
O5—Mn2—O10	90.4 (4)	C24C—N3C—C23C	114.3 (19)
N1—Mn2—O10	86.3 (4)	N3C—C23C—H23G	109.5
O9—Mn2—O10	173.0 (4)	N3C—C23C—H23H	109.5
O3—Mn2—Na1^i^	135.89 (7)	H23G—C23C—H23H	109.5
O4—Mn2—Na1^i^	41.44 (7)	N3C—C23C—H23I	109.5
O5—Mn2—Na1^i^	105.92 (6)	H23G—C23C—H23I	109.5
N1—Mn2—Na1^i^	64.73 (8)	H23H—C23C—H23I	109.5
O9—Mn2—Na1^i^	118.86 (11)	N3C—C24C—H24G	109.5
O10—Mn2—Na1^i^	54.6 (4)	N3C—C24C—H24H	109.5
N2—O4—Mn2	113.37 (16)	H24G—C24C—H24H	109.5
N2—O4—Na1^i^	109.1 (2)	N3C—C24C—H24I	109.5
Mn2—O4—Na1^i^	108.25 (10)	H24G—C24C—H24I	109.5
N2—O4—Na1	117.9 (2)	H24H—C24C—H24I	109.5
Mn2—O4—Na1	118.03 (9)	C25—O10—Mn2	129.0 (12)
Na1^i^—O4—Na1	85.62 (7)	C25—O10—Na1^i^	118.5 (11)
C8—N2—O4	113.7 (2)	Mn2—O10—Na1^i^	85.8 (4)
C8—N2—Mn1^i^	129.8 (2)	O10—C25—N4	128.2 (11)
O4—N2—Mn1^i^	116.43 (18)	O10—C25—H25	115.9
C8—O5—Mn2	111.17 (17)	N4—C25—H25	115.9
N2—C8—O5	119.5 (2)	C25—N4—C26	124.1 (8)
N2—C8—C9	120.0 (3)	C25—N4—C27	122.8 (8)
O5—C8—C9	120.4 (2)	C26—N4—C27	112.4 (8)
C14—C9—C10	119.4 (3)	N4—C26—H26A	109.5
C14—C9—C8	122.6 (3)	N4—C26—H26B	109.5
C10—C9—C8	118.0 (3)	H26A—C26—H26B	109.5
C11—C10—C9	121.4 (3)	N4—C26—H26C	109.5
C11—C10—H10	119.3	H26A—C26—H26C	109.5
C9—C10—H10	119.3	H26B—C26—H26C	109.5
C10—C11—C12	119.1 (3)	N4—C27—H27A	109.5
C10—C11—H11	120.5	N4—C27—H27B	109.5
C12—C11—H11	120.5	H27A—C27—H27B	109.5
C13—C12—C11	120.7 (3)	N4—C27—H27C	109.5
C13—C12—H12	119.6	H27A—C27—H27C	109.5
C11—C12—H12	119.6	H27B—C27—H27C	109.5
C12—C13—C14	120.6 (3)	C25B—O10B—Mn2B	122 (3)
C12—C13—H13	119.7	O10B—C25B—N4B	128 (3)
C14—C13—H13	119.7	O10B—C25B—H25B	115.8
O6—C14—C9	124.0 (3)	N4B—C25B—H25B	115.8
O6—C14—C13	117.1 (3)	C25B—N4B—C26B	125 (2)
C9—C14—C13	118.9 (3)	C25B—N4B—C27B	122 (2)
C14—O6—Mn1^i^	127.7 (2)	C26B—N4B—C27B	113 (2)
C15—O7—Na1	136.2 (2)	N4B—C26B—H26D	109.5
C15—O8—Mn1	128.5 (2)	N4B—C26B—H26E	109.5
O7—C15—O8	126.6 (3)	H26D—C26B—H26E	109.5
O7—C15—C16	118.6 (3)	N4B—C26B—H26F	109.5
O8—C15—C16	114.8 (3)	H26D—C26B—H26F	109.5
C21—C16—C17	119.2 (3)	H26E—C26B—H26F	109.5
C21—C16—C15	120.3 (3)	N4B—C27B—H27D	109.5
C17—C16—C15	120.4 (4)	N4B—C27B—H27E	109.5
C18—C17—C16	120.0 (4)	H27D—C27B—H27E	109.5
C18—C17—H17	120.0	N4B—C27B—H27F	109.5
C16—C17—H17	120.0	H27D—C27B—H27F	109.5
C19—C18—C17	120.6 (4)	H27E—C27B—H27F	109.5
C19—C18—H18	119.7	O10C—C25C—N4C	123.6 (13)
C17—C18—H18	119.7	O10C—C25C—H25C	118.2
C20—C19—C18	119.1 (4)	N4C—C25C—H25C	118.2
C20—C19—H19	120.5	C25C—N4C—C26C	119.4 (9)
C18—C19—H19	120.5	C25C—N4C—C27C	122.6 (9)
C19—C20—C21	121.5 (4)	C26C—N4C—C27C	118.0 (9)
C19—C20—Cl1	118.8 (3)	N4C—C26C—H26G	109.5
C21—C20—Cl1	119.6 (3)	N4C—C26C—H26H	109.5
C16—C21—C20	119.7 (4)	H26G—C26C—H26H	109.5
C16—C21—H21	120.2	N4C—C26C—H26I	109.5
C20—C21—H21	120.2	H26G—C26C—H26I	109.5
O6B^i^—Mn1B—O1B	157.3 (13)	H26H—C26C—H26I	109.5
O6B^i^—Mn1B—O2B	97.0 (9)	N4C—C27C—H27G	109.5
O1B—Mn1B—O2B	82.3 (7)	N4C—C27C—H27H	109.5
O6B^i^—Mn1B—Na1	123.3 (8)	H27G—C27C—H27H	109.5
O1B—Mn1B—Na1	37.8 (7)	N4C—C27C—H27I	109.5
O2B—Mn1B—Na1	106.4 (6)	H27G—C27C—H27I	109.5
N2B^i^—Mn1B—Na1	62.9 (11)	H27H—C27C—H27I	109.5
O8B^i^—Mn1B—Na1	133.0 (8)	C28—O11—Na1	131.9 (9)
Na1^i^—Mn1B—Na1	54.83 (9)	O11—C28—N5	124.1 (14)
N1B—O1B—Mn1B	114.0 (12)	O11—C28—H28	117.9
N1B—O1B—Na1	116 (3)	N5—C28—H28	117.9
Mn1B—O1B—Na1	115.3 (10)	C30—N5—C29	96.8 (8)
Na1^i^—O1B—Na1	85.4 (5)	C30—N5—C28	141.0 (10)
C1B—N1B—O1B	112.7 (14)	C29—N5—C28	121.7 (9)
C1B—N1B—Mn2B	129.9 (15)	N5—C29—H29A	109.5
O1B—N1B—Mn2B	117.2 (12)	C30—C29—H29A	68.1
C1B—O2B—Mn1B	107.6 (13)	N5—C29—H29B	109.5
N1B—C1B—O2B	122.6 (16)	C30—C29—H29B	124.3
N1B—C1B—C2B	120.6 (17)	H29A—C29—H29B	109.5
O2B—C1B—C2B	116.7 (16)	N5—C29—H29C	109.5
C3B—C2B—C7B	119.3 (18)	C30—C29—H29C	124.1
C3B—C2B—C1B	116 (2)	H29A—C29—H29C	109.5
C7B—C2B—C1B	124 (2)	H29B—C29—H29C	109.5
C4B—C3B—C2B	120 (2)	N5—C30—H30A	109.5
C4B—C3B—H3B	119.9	C29—C30—H30A	67.7
C2B—C3B—H3B	119.9	N5—C30—H30B	109.5
C3B—C4B—C5B	121 (2)	C29—C30—H30B	122.9
C3B—C4B—H4B	119.7	H30A—C30—H30B	109.5
C5B—C4B—H4B	119.7	N5—C30—H30C	109.5
C4B—C5B—C6B	120 (2)	C29—C30—H30C	125.5
C4B—C5B—H5B	119.9	H30A—C30—H30C	109.5
C6B—C5B—H5B	119.9	H30B—C30—H30C	109.5
C5B—C6B—C7B	119 (2)	C28B—O11B—Na1	148.6 (14)
C5B—C6B—H6B	120.4	O11B—C28B—N5B	112.8 (13)
C7B—C6B—H6B	120.4	O11B—C28B—H28B	123.6
O3B—C7B—C6B	119 (2)	N5B—C28B—H28B	123.6
O3B—C7B—C2B	122 (2)	C30B—N5B—C29B	96.1 (9)
C6B—C7B—C2B	119.1 (19)	C30B—N5B—C28B	137.8 (11)
C7B—O3B—Mn2B	130.8 (17)	C29B—N5B—C28B	126.2 (10)
O4B—Mn2B—O3B	175.4 (11)	N5B—C29B—H29D	109.5
O4B—Mn2B—O5B	84.3 (7)	C30B—C29B—H29D	89.2
O3B—Mn2B—O5B	97.7 (8)	N5B—C29B—H29E	109.5
O4B—Mn2B—N1B	88.4 (8)	C30B—C29B—H29E	86.1
O3B—Mn2B—N1B	89.1 (9)	H29D—C29B—H29E	109.5
O5B—Mn2B—N1B	170.3 (15)	N5B—C29B—H29F	109.5
O4B—Mn2B—O9B	92.2 (11)	C30B—C29B—H29F	148.8
O3B—Mn2B—O9B	91.9 (11)	H29D—C29B—H29F	109.5
O5B—Mn2B—O9B	89.5 (12)	H29E—C29B—H29F	109.5
N1B—Mn2B—O9B	97 (2)	N5B—C30B—H30D	109.5
O4B—Mn2B—O10B	80.9 (12)	C29B—C30B—H30D	65.3
O3B—Mn2B—O10B	95.2 (12)	N5B—C30B—H30E	109.5
O5B—Mn2B—O10B	84.4 (13)	C29B—C30B—H30E	119.3
N1B—Mn2B—O10B	88 (2)	H30D—C30B—H30E	109.5
O9B—Mn2B—O10B	171.2 (12)	N5B—C30B—H30F	109.5
N2B—O4B—Mn2B	111.7 (13)	C29B—C30B—H30F	129.9
N2B—O4B—Na1	121.3 (16)	H30D—C30B—H30F	109.5
Mn2B—O4B—Na1	122.1 (9)	H30E—C30B—H30F	109.5
			
O6^i^—Mn1—O1—N1	−93.3 (4)	Mn1B—O1B—N1B—C1B	8 (7)
O2—Mn1—O1—N1	−8.2 (2)	Na1^i^—O1B—N1B—C1B	136 (5)
N2^i^—Mn1—O1—N1	−168.8 (2)	Na1—O1B—N1B—C1B	−130 (4)
O8—Mn1—O1—N1	85.9 (2)	Mn1B—O1B—N1B—Mn2B	−176 (3)
Na1—Mn1—O1—N1	144.9 (2)	Na1^i^—O1B—N1B—Mn2B	−48 (5)
O6^i^—Mn1—O1—Na1	121.7 (4)	Na1—O1B—N1B—Mn2B	47 (5)
O2—Mn1—O1—Na1	−153.16 (11)	O1B—N1B—C1B—O2B	−2 (8)
N2^i^—Mn1—O1—Na1	46.26 (16)	Mn2B—N1B—C1B—O2B	−178 (4)
O8—Mn1—O1—Na1	−59.08 (11)	O1B—N1B—C1B—C2B	−178 (4)
O6^i^—Mn1—O1—Na1^i^	27.4 (4)	Mn2B—N1B—C1B—C2B	6 (9)
O2—Mn1—O1—Na1^i^	112.53 (11)	Mn1B—O2B—C1B—N1B	−4 (6)
N2^i^—Mn1—O1—Na1^i^	−48.05 (16)	Mn1B—O2B—C1B—C2B	172 (3)
O8—Mn1—O1—Na1^i^	−153.39 (10)	N1B—C1B—C2B—C3B	177 (5)
Na1—Mn1—O1—Na1^i^	−94.32 (11)	O2B—C1B—C2B—C3B	1 (6)
Mn1—O1—N1—C1	8.2 (3)	N1B—C1B—C2B—C7B	9 (7)
Na1—O1—N1—C1	149.4 (2)	O2B—C1B—C2B—C7B	−168 (4)
Na1^i^—O1—N1—C1	−117.9 (2)	C7B—C2B—C3B—C4B	−8 (7)
Mn1—O1—N1—Mn2	179.54 (12)	C1B—C2B—C3B—C4B	−177 (4)
Na1—O1—N1—Mn2	−39.2 (3)	C2B—C3B—C4B—C5B	9 (8)
Na1^i^—O1—N1—Mn2	53.5 (2)	C3B—C4B—C5B—C6B	−11 (9)
O1—N1—C1—O2	−2.0 (4)	C4B—C5B—C6B—C7B	12 (9)
Mn2—N1—C1—O2	−171.8 (2)	C5B—C6B—C7B—O3B	173 (5)
O1—N1—C1—C2	178.0 (3)	C5B—C6B—C7B—C2B	−10 (7)
Mn2—N1—C1—C2	8.2 (5)	C3B—C2B—C7B—O3B	−175 (4)
Mn1—O2—C1—N1	−4.7 (3)	C1B—C2B—C7B—O3B	−6 (7)
Mn1—O2—C1—C2	175.3 (2)	C3B—C2B—C7B—C6B	8 (7)
N1—C1—C2—C3	179.8 (3)	C1B—C2B—C7B—C6B	177 (4)
O2—C1—C2—C3	−0.2 (4)	C6B—C7B—O3B—Mn2B	166 (3)
N1—C1—C2—C7	0.3 (5)	C2B—C7B—O3B—Mn2B	−11 (6)
O2—C1—C2—C7	−179.7 (3)	C7B—O3B—Mn2B—O5B	−168 (3)
C7—C2—C3—C4	0.4 (5)	C7B—O3B—Mn2B—N1B	19 (4)
C1—C2—C3—C4	−179.1 (3)	C7B—O3B—Mn2B—O9B	−79 (3)
C2—C3—C4—C5	0.5 (6)	C7B—O3B—Mn2B—O10B	106 (3)
C3—C4—C5—C6	−1.3 (7)	C7B—O3B—Mn2B—Na1^i^	72 (4)
C4—C5—C6—C7	1.1 (7)	O5B—Mn2B—O4B—N2B	−6 (3)
C5—C6—C7—O3	179.7 (4)	N1B—Mn2B—O4B—N2B	168 (3)
C5—C6—C7—C2	−0.2 (6)	O9B—Mn2B—O4B—N2B	−95 (3)
C3—C2—C7—O3	179.6 (3)	O10B—Mn2B—O4B—N2B	79 (3)
C1—C2—C7—O3	−0.9 (5)	Na1^i^—Mn2B—O4B—N2B	114 (3)
C3—C2—C7—C6	−0.6 (5)	O5B—Mn2B—O4B—Na1	149.3 (15)
C1—C2—C7—C6	178.9 (3)	N1B—Mn2B—O4B—Na1	−37 (2)
C6—C7—O3—Mn2	172.7 (3)	O9B—Mn2B—O4B—Na1	60.0 (14)
C2—C7—O3—Mn2	−7.5 (5)	O10B—Mn2B—O4B—Na1	−125.4 (15)
C7—O3—Mn2—O5	−165.5 (3)	Na1^i^—Mn2B—O4B—Na1	−90.4 (10)
C7—O3—Mn2—N1	11.2 (3)	O5B—Mn2B—O4B—Na1^i^	−120.3 (11)
C7—O3—Mn2—O9	99.5 (3)	N1B—Mn2B—O4B—Na1^i^	53 (2)
C7—O3—Mn2—O10	−75.1 (5)	O9B—Mn2B—O4B—Na1^i^	150.4 (10)
C7—O3—Mn2—Na1^i^	−41.0 (3)	O10B—Mn2B—O4B—Na1^i^	−35.1 (11)
O5—Mn2—O4—N2	4.1 (3)	Mn2B—O4B—N2B—C8B	1 (5)
N1—Mn2—O4—N2	−173.0 (3)	Na1—O4B—N2B—C8B	−155 (3)
O9—Mn2—O4—N2	98.6 (3)	Na1^i^—O4B—N2B—C8B	113 (4)
O10—Mn2—O4—N2	−86.7 (4)	Mn2B—O4B—N2B—Mn1B^i^	−171.8 (18)
Na1^i^—Mn2—O4—N2	−121.2 (3)	Na1—O4B—N2B—Mn1B^i^	33 (4)
O5—Mn2—O4—Na1^i^	125.30 (10)	Na1^i^—O4B—N2B—Mn1B^i^	−60 (3)
N1—Mn2—O4—Na1^i^	−51.81 (11)	O4B—N2B—C8B—O5B	10 (6)
O9—Mn2—O4—Na1^i^	−140.15 (12)	Mn1B^i^—N2B—C8B—O5B	180 (3)
O10—Mn2—O4—Na1^i^	34.6 (4)	O4B—N2B—C8B—C9B	−177 (4)
O5—Mn2—O4—Na1	−139.85 (12)	Mn1B^i^—N2B—C8B—C9B	−6 (7)
N1—Mn2—O4—Na1	43.04 (13)	Mn2B—O5B—C8B—N2B	−14 (5)
O9—Mn2—O4—Na1	−45.30 (14)	Mn2B—O5B—C8B—C9B	173 (3)
O10—Mn2—O4—Na1	129.4 (4)	N2B—C8B—C9B—C14B	9 (7)
Na1^i^—Mn2—O4—Na1	94.85 (11)	O5B—C8B—C9B—C14B	−177 (4)
Mn2—O4—N2—C8	−4.4 (4)	N2B—C8B—C9B—C10B	−169 (4)
Na1^i^—O4—N2—C8	−125.2 (3)	O5B—C8B—C9B—C10B	5 (6)
Na1—O4—N2—C8	139.5 (3)	C14B—C9B—C10B—C11B	9 (8)
Mn2—O4—N2—Mn1^i^	172.36 (18)	C8B—C9B—C10B—C11B	−172 (5)
Na1^i^—O4—N2—Mn1^i^	51.6 (3)	C9B—C10B—C11B—C12B	−15 (9)
Na1—O4—N2—Mn1^i^	−43.7 (4)	C10B—C11B—C12B—C13B	13 (10)
O4—N2—C8—O5	1.8 (5)	C11B—C12B—C13B—C14B	−6 (9)
Mn1^i^—N2—C8—O5	−174.4 (3)	C10B—C9B—C14B—O6B	175 (4)
O4—N2—C8—C9	−177.1 (3)	C8B—C9B—C14B—O6B	−3 (8)
Mn1^i^—N2—C8—C9	6.6 (6)	C10B—C9B—C14B—C13B	−2 (8)
Mn2—O5—C8—N2	1.5 (4)	C8B—C9B—C14B—C13B	180 (4)
Mn2—O5—C8—C9	−179.5 (2)	C12B—C13B—C14B—O6B	−176 (5)
N2—C8—C9—C14	9.0 (5)	C12B—C13B—C14B—C9B	1 (8)
O5—C8—C9—C14	−170.0 (3)	C9B—C14B—O6B—Mn1B^i^	−6 (7)
N2—C8—C9—C10	−172.1 (3)	C13B—C14B—O6B—Mn1B^i^	171 (3)
O5—C8—C9—C10	8.9 (4)	Na1—O7B—C15B—O8B	65 (5)
C14—C9—C10—C11	1.0 (5)	Na1—O7B—C15B—C16B	−158 (2)
C8—C9—C10—C11	−177.9 (3)	Mn1B^i^—O8B—C15B—O7B	−55 (4)
C9—C10—C11—C12	−0.1 (5)	Mn1B^i^—O8B—C15B—C16B	165.6 (15)
C10—C11—C12—C13	−0.1 (6)	O7B—C15B—C16B—C21B	−149 (3)
C11—C12—C13—C14	−0.7 (5)	O8B—C15B—C16B—C21B	−9 (3)
C10—C9—C14—O6	−179.5 (3)	O7B—C15B—C16B—C17B	31 (3)
C8—C9—C14—O6	−0.6 (5)	O8B—C15B—C16B—C17B	170 (3)
C10—C9—C14—C13	−1.7 (4)	C21B—C16B—C17B—C18B	0.0 (4)
C8—C9—C14—C13	177.1 (3)	C15B—C16B—C17B—C18B	−179.9 (3)
C12—C13—C14—O6	179.5 (3)	C16B—C17B—C18B—C19B	0.0 (7)
C12—C13—C14—C9	1.6 (5)	C17B—C18B—C19B—C20B	0.0 (9)
C9—C14—O6—Mn1^i^	−24.5 (4)	C18B—C19B—C20B—C21B	0.0 (10)
C13—C14—O6—Mn1^i^	157.7 (2)	C18B—C19B—C20B—Cl1B	−179.8 (6)
Na1—O7—C15—O8	−30.3 (6)	C17B—C16B—C21B—C20B	−0.1 (7)
Na1—O7—C15—C16	147.0 (3)	C15B—C16B—C21B—C20B	179.9 (5)
Mn1—O8—C15—O7	10.4 (5)	C19B—C20B—C21B—C16B	0.1 (9)
Mn1—O8—C15—C16	−167.0 (2)	Cl1B—C20B—C21B—C16B	179.8 (5)
O7—C15—C16—C21	−172.1 (4)	Mn2—O9—C22—N3	176.2 (6)
O8—C15—C16—C21	5.5 (5)	O9—C22—N3—C24	177.2 (7)
O7—C15—C16—C17	4.4 (6)	O9—C22—N3—C23	−3.0 (11)
O8—C15—C16—C17	−178.1 (4)	Mn2B—O9B—C22B—N3B	168 (7)
C21—C16—C17—C18	0.0 (6)	O9B—C22B—N3B—C24B	177 (7)
C15—C16—C17—C18	−176.5 (4)	O9B—C22B—N3B—C23B	20 (13)
C16—C17—C18—C19	1.4 (7)	O9C—C22C—N3C—C24C	−6 (6)
C17—C18—C19—C20	−1.9 (7)	O9C—C22C—N3C—C23C	−178 (4)
C18—C19—C20—C21	1.1 (7)	Mn2—O10—C25—N4	−177.7 (9)
C18—C19—C20—Cl1	177.2 (4)	Na1^i^—O10—C25—N4	−68.3 (17)
C17—C16—C21—C20	−0.8 (6)	O10—C25—N4—C26	3 (2)
C15—C16—C21—C20	175.7 (4)	O10—C25—N4—C27	172.9 (15)
C19—C20—C21—C16	0.2 (7)	Mn2B—O10B—C25B—N4B	−170 (3)
Cl1—C20—C21—C16	−175.8 (3)	Na1^i^—O10B—C25B—N4B	−4 (5)
O6B^i^—Mn1B—O1B—N1B	−97 (4)	O10B—C25B—N4B—C26B	0.0 (4)
O2B—Mn1B—O1B—N1B	−8 (4)	O10B—C25B—N4B—C27B	180.0 (4)
N2B^i^—Mn1B—O1B—N1B	178 (4)	Na1^i^—O10C—C25C—N4C	−40.6 (17)
O8B^i^—Mn1B—O1B—N1B	77 (4)	O10C—C25C—N4C—C26C	−0.2 (4)
Na1^i^—Mn1B—O1B—N1B	126 (4)	O10C—C25C—N4C—C27C	−179.9 (4)
Na1—Mn1B—O1B—N1B	−138 (4)	Na1—O11—C28—N5	−125.3 (14)
O6B^i^—Mn1B—O1B—Na1^i^	136 (2)	O11—C28—N5—C30	175.4 (15)
O2B—Mn1B—O1B—Na1^i^	−134.1 (13)	O11—C28—N5—C29	5.8 (15)
N2B^i^—Mn1B—O1B—Na1^i^	52.0 (16)	C28—N5—C29—C30	173.4 (12)
O8B^i^—Mn1B—O1B—Na1^i^	−49.0 (13)	C28—N5—C30—C29	−171.1 (16)
Na1—Mn1B—O1B—Na1^i^	96.1 (11)	Na1—O11B—C28B—N5B	137.6 (17)
O6B^i^—Mn1B—O1B—Na1	40 (3)	O11B—C28B—N5B—C30B	−173.2 (14)
O2B—Mn1B—O1B—Na1	129.8 (13)	O11B—C28B—N5B—C29B	8.4 (15)
N2B^i^—Mn1B—O1B—Na1	−44.1 (16)	C28B—N5B—C29B—C30B	178.9 (14)
O8B^i^—Mn1B—O1B—Na1	−145.1 (12)	C28B—N5B—C30B—C29B	−178.6 (17)
Na1^i^—Mn1B—O1B—Na1	−96.1 (10)		

Symmetry code: (i) −*x*+1, −*y*+1, −*z*+1.

### Bis(µ-3-chlorobenzoato)hexakis(dimethylformamide)tetrakis(µ4-N,2-dioxidobenzene-1-carboximidato)tetramanganese(III)disodium(I) (1) Hydrogen-bond geometry (Å, º)

**Table:**

*D*—H···*A*	*D*—H	H···*A*	*D*···*A*	*D*—H···*A*
C26—H26*A*···O11^i^	0.98	2.65	3.56 (2)	155
C29—H29*A*···Cl1^ii^	0.98	2.78	3.702 (10)	156
C30—H30*A*···Cl1^ii^	0.98	2.79	3.699 (14)	154
C30—H30*C*···O6	0.98	2.54	3.125 (16)	119

Symmetry codes: (i) −*x*+1, −*y*+1, −*z*+1; (ii) *x*+1, *y*, *z*+1.

### Tetra-µ-aqua-tris(µ-3-chlorobenzoato)(dimethylformamide)tetrakis(µ4-N,2-dioxidobenzene-1-carboximidato)pentamanganese(III)sodium(I) dimethylformamide tetraolvate 0.72-hydrate (2) Crystal data

**Table:**

[Mn_5_Na(C_7_H_4_ClO_2_)_3_(C_7_H_4_NO_3_)_4_(C_3_H_7_NO)(H_2_O)_4_]·4C_3_H_7_NO·0.718H_2_O	*F*(000) = 1856
*M**_r_* = 1815.30	*D*_x_ = 1.569 Mg m^−^^3^
Monoclinic, *P**n*	Mo *K*α radiation, λ = 0.71073 Å
*a* = 14.1955 (9) Å	Cell parameters from 9812 reflections
*b* = 16.3349 (11) Å	θ = 2.8–33.2°
*c* = 16.6144 (10) Å	µ = 1.00 mm^−^^1^
β = 94.235 (2)°	*T* = 150 K
*V* = 3842.1 (4) Å^3^	Plate, brown
*Z* = 2	0.45 × 0.23 × 0.09 mm

### Tetra-µ-aqua-tris(µ-3-chlorobenzoato)(dimethylformamide)tetrakis(µ4-N,2-dioxidobenzene-1-carboximidato)pentamanganese(III)sodium(I) dimethylformamide tetraolvate 0.72-hydrate (2) Data collection

**Table:**

Bruker AXS D8 Quest CMOS diffractometer	27291 independent reflections
Radiation source: fine focus sealed tube X-ray source	24179 reflections with *I* > 2σ(*I*)
Triumph curved graphite crystal monochromator	*R*_int_ = 0.038
Detector resolution: 10.4167 pixels mm^-1^	θ_max_ = 33.3°, θ_min_ = 2.3°
ω and phi scans	*h* = −21→21
Absorption correction: multi-scan (SADABS; Krause *et al.*, 2015)	*k* = −24→25
*T*_min_ = 0.636, *T*_max_ = 0.747	*l* = −25→25
118654 measured reflections	

### Tetra-µ-aqua-tris(µ-3-chlorobenzoato)(dimethylformamide)tetrakis(µ4-N,2-dioxidobenzene-1-carboximidato)pentamanganese(III)sodium(I) dimethylformamide tetraolvate 0.72-hydrate (2) Refinement

**Table:**

Refinement on *F*^2^	Secondary atom site location: difference Fourier map
Least-squares matrix: full	Hydrogen site location: mixed
*R*[*F*^2^ > 2σ(*F*^2^)] = 0.034	H atoms treated by a mixture of independent and constrained refinement
*wR*(*F*^2^) = 0.091	*w* = 1/[σ^2^(*F*_o_^2^) + (0.0522*P*)^2^ + 0.9214*P*] where *P* = (*F*_o_^2^ + 2*F*_c_^2^)/3
*S* = 1.03	(Δ/σ)_max_ = 0.001
27291 reflections	Δρ_max_ = 0.82 e Å^−^^3^
1079 parameters	Δρ_min_ = −0.69 e Å^−^^3^
143 restraints	Absolute structure: Flack *x* determined using 10010 quotients [(*I*^+^)-(*I*^-^)]/[(*I*^+^)+(*I*^-^)] (Parsons *et al.*, 2013)
Primary atom site location: structure-invariant direct methods	Absolute structure parameter: 0.000 (2)

### Tetra-µ-aqua-tris(µ-3-chlorobenzoato)(dimethylformamide)tetrakis(µ4-N,2-dioxidobenzene-1-carboximidato)pentamanganese(III)sodium(I) dimethylformamide tetraolvate 0.72-hydrate (2) Special details

**Table:**

Geometry. All esds (except the esd in the dihedral angle between two l.s. planes) are estimated using the full covariance matrix. The cell esds are taken into account individually in the estimation of esds in distances, angles and torsion angles; correlations between esds in cell parameters are only used when they are defined by crystal symmetry. An approximate (isotropic) treatment of cell esds is used for estimating esds involving l.s. planes.
Refinement. A partially occupied water molecule (O28) induces disorder for a neighboring DMF molecule (of O27). The two disordered moieties were restrained to have similar geometries, and the carbon, oxygen, and nitrogen atoms of the DMF molecule restrained to have similar Uij components of the ADPs (esd = 0.01 Angstrom squared; SIMU restraint in Shexl). Subject to these conditions the occupancy ratio refined to 0.718 (6) to 0.282 (6). Water H atom positions were refined and O-H and H···H distances were restrained to 0.84 (2) and 1.36 (2) Angstrom, respectively. The water H atom positions of partially occupied O28 were further restrained based on hydrogen bonding considerations. All other hydrogen atoms were placed in calculated positions and refined as riding on their carrier atoms with C-H distances of 0.95 Angstrom for sp2 carbon atoms and 0.98 Angstrom for methyl carbon atoms. The Uiso values for hydrogen atoms were set to a multiple of the value of the carrying carbon atom (1.2 times for sp2 hybridized carbon atoms or 1.5 times for methyl carbon atoms).

### Tetra-µ-aqua-tris(µ-3-chlorobenzoato)(dimethylformamide)tetrakis(µ4-N,2-dioxidobenzene-1-carboximidato)pentamanganese(III)sodium(I) dimethylformamide tetraolvate 0.72-hydrate (2) Fractional atomic coordinates and isotropic or equivalent isotropic displacement parameters (Å^2^)

**Table:**

	*x*	*y*	*z*	*U*_iso_*/*U*_eq_	Occ. (<1)
Mn1	0.94848 (3)	0.74796 (2)	0.12192 (2)	0.01712 (7)	
Mn2	0.88182 (3)	0.55381 (2)	0.20529 (2)	0.01784 (7)	
Mn3	0.84120 (3)	0.80306 (2)	0.32631 (2)	0.02092 (8)	
Mn4	0.79257 (3)	0.92045 (2)	0.07682 (2)	0.01758 (7)	
Mn5	0.80131 (3)	0.66570 (2)	−0.04269 (2)	0.01977 (7)	
Cl1	1.43537 (7)	0.72511 (8)	0.22049 (9)	0.0627 (3)	
Cl2	1.36255 (7)	0.88134 (7)	0.04017 (8)	0.0566 (3)	
Cl3	1.37297 (7)	0.65953 (6)	0.00448 (8)	0.0499 (2)	
Na1	0.72158 (8)	0.72202 (7)	0.14880 (7)	0.0237 (2)	
O1	0.87675 (13)	0.66896 (10)	0.22245 (10)	0.0181 (3)	
O2	0.87704 (14)	0.55137 (12)	0.32112 (11)	0.0224 (3)	
O3	0.82492 (18)	0.77918 (14)	0.43375 (12)	0.0327 (5)	
O4	0.85092 (14)	0.82488 (11)	0.21559 (11)	0.0221 (4)	
O5	0.79567 (16)	0.91481 (12)	0.32656 (11)	0.0263 (4)	
O6	0.74502 (16)	1.02397 (12)	0.09454 (11)	0.0259 (4)	
O7	0.82177 (13)	0.80836 (11)	0.05780 (10)	0.0174 (3)	
O8	0.75439 (13)	0.91719 (11)	−0.03804 (11)	0.0197 (3)	
O9	0.73472 (17)	0.67929 (12)	−0.14280 (12)	0.0279 (4)	
O10	0.85109 (13)	0.65224 (11)	0.06581 (10)	0.0188 (3)	
O11	0.80267 (15)	0.54635 (11)	−0.03933 (12)	0.0239 (4)	
O12	0.87122 (16)	0.44136 (11)	0.19019 (12)	0.0256 (4)	
O13	1.06930 (15)	0.68575 (14)	0.17489 (15)	0.0333 (5)	
O14	1.03540 (14)	0.55599 (13)	0.20912 (13)	0.0266 (4)	
O15	1.01555 (14)	0.86423 (12)	0.12682 (12)	0.0250 (4)	
O16	0.93343 (15)	0.96135 (13)	0.05707 (13)	0.0286 (4)	
O17	1.01332 (17)	0.73627 (14)	0.00900 (13)	0.0314 (4)	
O18	0.93645 (17)	0.66595 (14)	−0.09162 (13)	0.0304 (4)	
O19	0.70789 (16)	0.57678 (14)	0.19746 (13)	0.0282 (4)	
H19A	0.686 (3)	0.571 (3)	0.2404 (19)	0.042*	
H19B	0.672 (3)	0.551 (3)	0.165 (2)	0.042*	
O20	0.68174 (15)	0.76328 (14)	0.28274 (13)	0.0281 (4)	
H20A	0.665 (3)	0.725 (2)	0.310 (3)	0.042*	
H20B	0.645 (3)	0.804 (2)	0.289 (3)	0.042*	
O21	0.63857 (15)	0.85442 (15)	0.10221 (13)	0.0299 (4)	
H21A	0.602 (3)	0.850 (3)	0.061 (2)	0.045*	
H21B	0.599 (3)	0.882 (3)	0.127 (3)	0.045*	
O22	0.65301 (15)	0.66842 (13)	0.02323 (14)	0.0281 (4)	
H22A	0.615 (3)	0.699 (3)	−0.004 (3)	0.042*	
H22B	0.626 (3)	0.6235 (19)	0.028 (3)	0.042*	
O23	0.98758 (19)	0.84110 (16)	0.36432 (15)	0.0389 (5)	
O24	0.63713 (17)	0.61014 (15)	0.34356 (13)	0.0329 (4)	
O25	0.56256 (19)	0.89325 (19)	0.24735 (16)	0.0446 (6)	
O26	0.54987 (17)	0.79780 (16)	−0.03879 (16)	0.0369 (5)	
O28	0.5391 (3)	0.6059 (3)	−0.1628 (3)	0.0623 (14)	0.718 (6)
H28A	0.497 (5)	0.6239 (18)	−0.127 (4)	0.093*	0.718 (6)
H28B	0.598 (3)	0.624 (6)	−0.148 (5)	0.093*	0.718 (6)
N1	0.87138 (15)	0.68908 (13)	0.30410 (12)	0.0173 (3)	
N2	0.81668 (16)	0.90282 (13)	0.19300 (12)	0.0195 (4)	
N3	0.79729 (15)	0.78406 (12)	−0.02184 (11)	0.0163 (3)	
N4	0.86362 (15)	0.57017 (12)	0.08839 (12)	0.0174 (3)	
N5	1.1422 (2)	0.86518 (17)	0.34591 (17)	0.0360 (6)	
N6	0.61309 (18)	0.59868 (17)	0.47635 (15)	0.0290 (5)	
N7	0.4892 (3)	1.0103 (3)	0.2817 (2)	0.0583 (11)	
N8	0.4921 (2)	0.86435 (19)	−0.1525 (2)	0.0380 (6)	
C1	0.86952 (16)	0.62440 (15)	0.35126 (14)	0.0187 (4)	
C2	0.86233 (18)	0.63451 (17)	0.43869 (14)	0.0210 (4)	
C3	0.8751 (2)	0.56420 (18)	0.48703 (16)	0.0261 (5)	
H3	0.884687	0.512773	0.462218	0.031*	
C4	0.8740 (2)	0.5686 (2)	0.57046 (17)	0.0321 (6)	
H4	0.882371	0.520732	0.602696	0.038*	
C5	0.8604 (2)	0.6443 (2)	0.60579 (17)	0.0358 (7)	
H5	0.861196	0.648234	0.662886	0.043*	
C6	0.8456 (2)	0.7142 (2)	0.55925 (16)	0.0332 (6)	
H6	0.835577	0.765191	0.584798	0.040*	
C7	0.8454 (2)	0.71057 (18)	0.47441 (15)	0.0248 (5)	
C8	0.78751 (19)	0.94529 (15)	0.25412 (15)	0.0213 (4)	
C9	0.7475 (2)	1.02746 (16)	0.24081 (15)	0.0231 (5)	
C10	0.7290 (3)	1.07315 (19)	0.30896 (18)	0.0354 (7)	
H10	0.740587	1.049507	0.360992	0.042*	
C11	0.6941 (4)	1.1521 (2)	0.3022 (2)	0.0518 (11)	
H11	0.682379	1.182691	0.349058	0.062*	
C12	0.6762 (3)	1.1862 (2)	0.2253 (2)	0.0452 (9)	
H12	0.652336	1.240365	0.219919	0.054*	
C13	0.6930 (2)	1.14180 (17)	0.15718 (17)	0.0305 (6)	
H13	0.679519	1.165580	0.105426	0.037*	
C14	0.7298 (2)	1.06154 (15)	0.16324 (15)	0.0217 (4)	
C15	0.76197 (17)	0.84357 (14)	−0.06743 (13)	0.0163 (4)	
C16	0.73348 (17)	0.82784 (15)	−0.15250 (14)	0.0188 (4)	
C17	0.7173 (2)	0.89589 (17)	−0.20221 (16)	0.0266 (5)	
H17	0.723889	0.949159	−0.179553	0.032*	
C18	0.6918 (3)	0.8877 (2)	−0.28359 (18)	0.0364 (7)	
H18	0.683475	0.934520	−0.317235	0.044*	
C19	0.6785 (3)	0.8092 (2)	−0.31541 (18)	0.0370 (7)	
H19	0.658535	0.802855	−0.370895	0.044*	
C20	0.6938 (2)	0.74110 (18)	−0.26811 (16)	0.0278 (5)	
H20	0.684501	0.688270	−0.291265	0.033*	
C21	0.72295 (18)	0.74846 (15)	−0.18567 (14)	0.0197 (4)	
C22	0.83631 (17)	0.51901 (15)	0.03055 (15)	0.0190 (4)	
C23	0.84626 (17)	0.43050 (14)	0.04471 (15)	0.0197 (4)	
C24	0.8402 (2)	0.37860 (16)	−0.02240 (17)	0.0245 (5)	
H24	0.829900	0.401564	−0.074839	0.029*	
C25	0.8490 (2)	0.29459 (17)	−0.0142 (2)	0.0306 (6)	
H25	0.844732	0.260176	−0.060464	0.037*	
C26	0.8642 (2)	0.26108 (17)	0.0632 (2)	0.0319 (6)	
H26	0.869740	0.203475	0.069847	0.038*	
C27	0.8711 (2)	0.31183 (17)	0.13012 (19)	0.0297 (6)	
H27	0.881245	0.288262	0.182318	0.036*	
C28	0.86365 (18)	0.39730 (15)	0.12278 (16)	0.0208 (4)	
C29	1.09125 (18)	0.61447 (17)	0.19911 (16)	0.0231 (5)	
C30	1.19483 (18)	0.59768 (17)	0.21915 (16)	0.0232 (5)	
C31	1.2604 (2)	0.66010 (19)	0.20837 (19)	0.0291 (5)	
H31	1.240250	0.711423	0.186383	0.035*	
C32	1.3555 (2)	0.6458 (2)	0.2304 (2)	0.0343 (6)	
C33	1.3867 (2)	0.5711 (2)	0.26120 (19)	0.0326 (6)	
H33	1.452044	0.562114	0.275118	0.039*	
C34	1.3216 (2)	0.5096 (2)	0.27153 (18)	0.0296 (6)	
H34	1.342280	0.458220	0.292975	0.036*	
C35	1.2258 (2)	0.52263 (18)	0.25067 (17)	0.0256 (5)	
H35	1.181605	0.480122	0.257983	0.031*	
C36	1.00847 (19)	0.92630 (16)	0.08202 (16)	0.0229 (5)	
C37	1.09942 (19)	0.96143 (16)	0.05575 (15)	0.0228 (5)	
C38	1.1799 (2)	0.91298 (18)	0.06101 (17)	0.0275 (5)	
H38	1.177235	0.858381	0.080410	0.033*	
C39	1.2644 (2)	0.9450 (2)	0.0377 (2)	0.0318 (6)	
C40	1.2704 (2)	1.0247 (2)	0.01019 (18)	0.0318 (6)	
H40	1.328891	1.046305	−0.004390	0.038*	
C41	1.1894 (2)	1.07257 (18)	0.00434 (17)	0.0291 (6)	
H41	1.192473	1.127102	−0.015157	0.035*	
C42	1.1037 (2)	1.04164 (17)	0.02669 (16)	0.0255 (5)	
H42	1.048582	1.074773	0.022236	0.031*	
C43	1.0099 (2)	0.69213 (16)	−0.05311 (17)	0.0267 (5)	
C44	1.1038 (2)	0.66875 (17)	−0.08435 (17)	0.0273 (5)	
C45	1.1853 (2)	0.67586 (18)	−0.0333 (2)	0.0311 (6)	
H45	1.182075	0.696497	0.019891	0.037*	
C46	1.2717 (2)	0.65257 (19)	−0.0605 (2)	0.0353 (7)	
C47	1.2781 (3)	0.6242 (2)	−0.1388 (2)	0.0407 (8)	
H47	1.337575	0.609879	−0.157422	0.049*	
C48	1.1958 (3)	0.6172 (2)	−0.1893 (2)	0.0375 (7)	
H48	1.199314	0.597953	−0.242982	0.045*	
C49	1.1085 (3)	0.63816 (18)	−0.16235 (18)	0.0318 (6)	
H49	1.052487	0.631654	−0.196770	0.038*	
C50	1.0534 (3)	0.8454 (2)	0.3205 (2)	0.0372 (7)	
H50	1.040451	0.833992	0.264771	0.045*	
C51	1.1663 (3)	0.8825 (2)	0.4306 (2)	0.0439 (8)	
H51A	1.197463	0.936011	0.435834	0.066*	
H51B	1.209159	0.840091	0.453451	0.066*	
H51C	1.108709	0.883197	0.459604	0.066*	
C52	1.2180 (3)	0.8640 (3)	0.2919 (3)	0.0493 (9)	
H52A	1.194479	0.841259	0.239647	0.074*	
H52B	1.269913	0.829932	0.315277	0.074*	
H52C	1.240804	0.919863	0.284372	0.074*	
C53	0.6344 (2)	0.56990 (19)	0.40588 (18)	0.0287 (5)	
H53	0.648914	0.513254	0.402979	0.034*	
C54	0.5930 (3)	0.6854 (3)	0.4853 (3)	0.0496 (10)	
H54A	0.652425	0.715561	0.495198	0.074*	
H54B	0.553887	0.693345	0.530827	0.074*	
H54C	0.559159	0.705839	0.435730	0.074*	
C55	0.6146 (3)	0.5472 (3)	0.5480 (2)	0.0473 (9)	
H55A	0.630461	0.490895	0.533684	0.071*	
H55B	0.552273	0.548074	0.569726	0.071*	
H55C	0.662057	0.567908	0.588780	0.071*	
C56	0.5413 (3)	0.9439 (3)	0.2970 (3)	0.0561 (12)	
H56	0.564073	0.934855	0.351475	0.067*	
C57	0.4582 (5)	1.0323 (4)	0.1996 (3)	0.0798 (19)	
H57A	0.485907	0.994340	0.162163	0.120*	
H57B	0.478695	1.088213	0.188577	0.120*	
H57C	0.389196	1.029137	0.192349	0.120*	
C58	0.4721 (4)	1.0682 (5)	0.3470 (4)	0.086 (2)	
H58A	0.492393	1.123106	0.332017	0.129*	
H58B	0.507917	1.050939	0.396783	0.129*	
H58C	0.404530	1.069058	0.355617	0.129*	
C59	0.5163 (2)	0.7986 (2)	−0.1100 (2)	0.0371 (7)	
H59	0.507364	0.747153	−0.136144	0.045*	
C60	0.5023 (3)	0.9463 (2)	−0.1198 (3)	0.0470 (9)	
H60A	0.546487	0.977347	−0.150636	0.071*	
H60B	0.526599	0.943363	−0.063153	0.071*	
H60C	0.440667	0.973627	−0.123632	0.071*	
C61	0.4571 (4)	0.8571 (3)	−0.2372 (3)	0.0595 (12)	
H61A	0.484238	0.900789	−0.268536	0.089*	
H61B	0.388059	0.861744	−0.241617	0.089*	
H61C	0.475446	0.803758	−0.258093	0.089*	
O27	0.6052 (6)	0.5130 (3)	0.0592 (4)	0.0320 (11)	0.718 (6)
C62	0.6006 (3)	0.4733 (3)	−0.0046 (3)	0.0298 (8)	0.718 (6)
H62	0.594657	0.502532	−0.054164	0.036*	0.718 (6)
N9	0.6034 (3)	0.3918 (2)	−0.0072 (2)	0.0237 (7)	0.718 (6)
C63	0.6113 (4)	0.3432 (3)	0.0650 (3)	0.0378 (10)	0.718 (6)
H63A	0.566156	0.297783	0.059700	0.057*	0.718 (6)
H63B	0.597531	0.377271	0.111236	0.057*	0.718 (6)
H63C	0.675588	0.321378	0.073329	0.057*	0.718 (6)
C64	0.6022 (12)	0.3530 (7)	−0.0877 (6)	0.045 (3)	0.718 (6)
H64A	0.594719	0.395288	−0.129559	0.067*	0.718 (6)
H64B	0.549386	0.314372	−0.094251	0.067*	0.718 (6)
H64C	0.661727	0.323691	−0.092731	0.067*	0.718 (6)
O27B	0.616 (2)	0.5054 (13)	0.0773 (14)	0.061 (6)	0.282 (6)
C62B	0.6184 (8)	0.4417 (9)	0.0424 (8)	0.041 (2)	0.282 (6)
H62B	0.635682	0.396191	0.075905	0.049*	0.282 (6)
N9B	0.6016 (8)	0.4235 (9)	−0.0353 (8)	0.047 (3)	0.282 (6)
C63B	0.5828 (10)	0.4949 (11)	−0.0902 (9)	0.057 (4)	0.282 (6)
H63D	0.642836	0.517217	−0.105915	0.085*	0.282 (6)
H63E	0.548678	0.537152	−0.062287	0.085*	0.282 (6)
H63F	0.544488	0.477096	−0.138570	0.085*	0.282 (6)
C64B	0.599 (2)	0.3423 (13)	−0.0723 (17)	0.035 (5)	0.282 (6)
H64D	0.557796	0.343515	−0.122357	0.052*	0.282 (6)
H64E	0.574243	0.302599	−0.034928	0.052*	0.282 (6)
H64F	0.662842	0.326207	−0.084474	0.052*	0.282 (6)

### Tetra-µ-aqua-tris(µ-3-chlorobenzoato)(dimethylformamide)tetrakis(µ4-N,2-dioxidobenzene-1-carboximidato)pentamanganese(III)sodium(I) dimethylformamide tetraolvate 0.72-hydrate (2) Atomic displacement parameters (Å^2^)

**Table:**

	*U*^11^	*U*^22^	*U*^33^	*U*^12^	*U*^13^	*U*^23^
Mn1	0.02092 (15)	0.01406 (15)	0.01640 (14)	0.00043 (12)	0.00155 (11)	0.00134 (12)
Mn2	0.02549 (16)	0.01237 (15)	0.01608 (15)	0.00079 (12)	0.00443 (12)	0.00244 (12)
Mn3	0.03305 (19)	0.01683 (16)	0.01285 (14)	0.00786 (14)	0.00139 (13)	0.00027 (12)
Mn4	0.02744 (17)	0.01202 (15)	0.01300 (14)	0.00354 (13)	−0.00050 (12)	−0.00084 (11)
Mn5	0.03203 (19)	0.01313 (15)	0.01381 (14)	0.00237 (13)	−0.00060 (13)	−0.00134 (12)
Cl1	0.0331 (4)	0.0563 (6)	0.0964 (9)	−0.0184 (4)	−0.0115 (5)	0.0191 (6)
Cl2	0.0388 (4)	0.0540 (6)	0.0789 (8)	0.0107 (4)	0.0175 (5)	0.0049 (5)
Cl3	0.0371 (4)	0.0388 (5)	0.0747 (7)	−0.0005 (3)	0.0096 (4)	−0.0113 (4)
Na1	0.0287 (5)	0.0207 (5)	0.0222 (5)	0.0017 (4)	0.0046 (4)	0.0016 (4)
O1	0.0289 (8)	0.0132 (7)	0.0126 (7)	0.0023 (6)	0.0039 (6)	−0.0002 (5)
O2	0.0317 (9)	0.0178 (8)	0.0180 (8)	0.0020 (7)	0.0046 (7)	0.0030 (6)
O3	0.0571 (14)	0.0270 (10)	0.0143 (8)	0.0167 (10)	0.0049 (8)	0.0022 (7)
O4	0.0367 (10)	0.0145 (8)	0.0152 (7)	0.0095 (7)	0.0035 (7)	0.0020 (6)
O5	0.0431 (11)	0.0203 (9)	0.0152 (8)	0.0099 (8)	0.0007 (7)	−0.0009 (6)
O6	0.0463 (11)	0.0153 (8)	0.0157 (8)	0.0106 (8)	−0.0002 (7)	−0.0002 (6)
O7	0.0257 (8)	0.0144 (7)	0.0118 (6)	0.0013 (6)	−0.0005 (6)	−0.0025 (5)
O8	0.0293 (9)	0.0135 (7)	0.0159 (7)	0.0018 (6)	−0.0009 (6)	−0.0003 (6)
O9	0.0475 (12)	0.0165 (8)	0.0183 (8)	0.0005 (8)	−0.0062 (8)	−0.0019 (7)
O10	0.0296 (8)	0.0117 (7)	0.0149 (7)	0.0012 (6)	0.0012 (6)	0.0013 (6)
O11	0.0376 (10)	0.0144 (8)	0.0193 (8)	0.0025 (7)	−0.0003 (7)	−0.0017 (6)
O12	0.0425 (11)	0.0133 (8)	0.0218 (9)	−0.0002 (7)	0.0077 (8)	0.0034 (6)
O13	0.0239 (9)	0.0294 (11)	0.0461 (13)	0.0035 (8)	−0.0003 (9)	0.0146 (9)
O14	0.0233 (9)	0.0235 (9)	0.0331 (10)	0.0012 (7)	0.0031 (7)	−0.0009 (8)
O15	0.0310 (9)	0.0206 (9)	0.0230 (9)	−0.0066 (7)	−0.0004 (7)	0.0021 (7)
O16	0.0293 (9)	0.0240 (10)	0.0323 (10)	−0.0050 (7)	0.0006 (8)	0.0030 (8)
O17	0.0374 (11)	0.0285 (10)	0.0305 (10)	−0.0010 (8)	0.0170 (9)	−0.0079 (8)
O18	0.0393 (11)	0.0305 (11)	0.0226 (9)	0.0025 (8)	0.0097 (8)	−0.0028 (8)
O19	0.0326 (10)	0.0308 (11)	0.0220 (9)	−0.0035 (8)	0.0083 (8)	0.0001 (8)
O20	0.0267 (9)	0.0295 (10)	0.0286 (10)	0.0059 (8)	0.0052 (7)	0.0044 (8)
O21	0.0283 (10)	0.0340 (11)	0.0275 (10)	0.0060 (8)	0.0022 (8)	−0.0021 (8)
O22	0.0306 (10)	0.0203 (9)	0.0334 (10)	−0.0040 (7)	0.0025 (8)	0.0017 (8)
O23	0.0461 (13)	0.0329 (12)	0.0356 (12)	−0.0029 (10)	−0.0113 (10)	−0.0017 (9)
O24	0.0390 (11)	0.0357 (12)	0.0248 (9)	−0.0002 (9)	0.0075 (8)	0.0008 (8)
O25	0.0397 (13)	0.0560 (17)	0.0387 (13)	0.0115 (11)	0.0076 (10)	0.0007 (12)
O26	0.0323 (11)	0.0356 (12)	0.0420 (13)	−0.0004 (9)	−0.0024 (9)	0.0028 (10)
O28	0.047 (2)	0.061 (3)	0.081 (3)	−0.008 (2)	0.015 (2)	−0.015 (2)
N1	0.0230 (9)	0.0170 (9)	0.0120 (8)	0.0031 (7)	0.0019 (7)	0.0005 (7)
N2	0.0312 (10)	0.0126 (8)	0.0145 (8)	0.0060 (7)	0.0009 (7)	0.0001 (7)
N3	0.0245 (9)	0.0141 (8)	0.0103 (7)	0.0009 (7)	0.0006 (6)	−0.0010 (6)
N4	0.0235 (9)	0.0118 (8)	0.0171 (8)	0.0011 (6)	0.0035 (7)	0.0019 (7)
N5	0.0488 (15)	0.0284 (13)	0.0294 (12)	0.0025 (11)	−0.0073 (11)	−0.0042 (10)
N6	0.0276 (11)	0.0357 (13)	0.0244 (11)	−0.0027 (9)	0.0074 (9)	−0.0019 (9)
N7	0.0481 (18)	0.080 (3)	0.0462 (19)	0.0328 (19)	−0.0014 (15)	−0.0175 (18)
N8	0.0335 (13)	0.0356 (15)	0.0437 (16)	0.0034 (11)	−0.0053 (11)	−0.0023 (12)
C1	0.0184 (9)	0.0215 (11)	0.0165 (9)	0.0029 (8)	0.0022 (7)	0.0029 (8)
C2	0.0212 (10)	0.0259 (12)	0.0160 (9)	0.0046 (9)	0.0030 (8)	0.0040 (8)
C3	0.0286 (12)	0.0286 (13)	0.0214 (11)	0.0037 (10)	0.0044 (9)	0.0083 (10)
C4	0.0385 (15)	0.0379 (16)	0.0204 (11)	0.0077 (12)	0.0059 (11)	0.0113 (11)
C5	0.0440 (16)	0.0474 (19)	0.0166 (11)	0.0171 (14)	0.0059 (11)	0.0078 (11)
C6	0.0454 (16)	0.0395 (16)	0.0147 (10)	0.0142 (13)	0.0025 (10)	0.0024 (10)
C7	0.0301 (12)	0.0301 (13)	0.0142 (10)	0.0083 (10)	0.0021 (9)	0.0042 (9)
C8	0.0321 (12)	0.0159 (10)	0.0157 (9)	0.0051 (9)	−0.0005 (8)	−0.0011 (8)
C9	0.0345 (12)	0.0169 (10)	0.0174 (10)	0.0075 (9)	0.0002 (9)	−0.0033 (8)
C10	0.061 (2)	0.0251 (14)	0.0201 (12)	0.0167 (13)	0.0028 (12)	−0.0035 (10)
C11	0.100 (3)	0.0296 (16)	0.0257 (14)	0.0324 (19)	0.0044 (17)	−0.0060 (12)
C12	0.085 (3)	0.0230 (14)	0.0272 (14)	0.0277 (16)	0.0026 (16)	−0.0030 (11)
C13	0.0494 (17)	0.0179 (12)	0.0242 (12)	0.0126 (11)	0.0020 (11)	−0.0003 (9)
C14	0.0322 (12)	0.0134 (10)	0.0189 (10)	0.0045 (8)	−0.0013 (9)	−0.0019 (8)
C15	0.0216 (10)	0.0140 (9)	0.0135 (9)	−0.0001 (7)	0.0026 (7)	−0.0006 (7)
C16	0.0248 (10)	0.0180 (10)	0.0136 (9)	0.0005 (8)	0.0018 (8)	0.0015 (8)
C17	0.0436 (15)	0.0191 (11)	0.0164 (10)	0.0021 (10)	−0.0033 (10)	0.0022 (8)
C18	0.061 (2)	0.0290 (15)	0.0179 (11)	0.0055 (13)	−0.0065 (12)	0.0041 (10)
C19	0.064 (2)	0.0288 (15)	0.0163 (11)	0.0072 (14)	−0.0078 (12)	−0.0019 (10)
C20	0.0434 (15)	0.0240 (12)	0.0153 (10)	0.0023 (11)	−0.0020 (10)	−0.0038 (9)
C21	0.0256 (11)	0.0198 (11)	0.0136 (9)	0.0003 (8)	0.0016 (8)	−0.0016 (8)
C22	0.0223 (10)	0.0154 (10)	0.0199 (10)	0.0006 (8)	0.0047 (8)	−0.0018 (8)
C23	0.0214 (10)	0.0133 (9)	0.0248 (11)	−0.0006 (8)	0.0043 (8)	−0.0010 (8)
C24	0.0290 (12)	0.0165 (10)	0.0283 (12)	0.0010 (9)	0.0046 (10)	−0.0027 (9)
C25	0.0383 (15)	0.0153 (11)	0.0380 (15)	0.0002 (10)	0.0023 (12)	−0.0064 (10)
C26	0.0429 (16)	0.0138 (11)	0.0396 (16)	0.0000 (10)	0.0070 (13)	−0.0009 (10)
C27	0.0413 (15)	0.0142 (11)	0.0345 (14)	0.0010 (10)	0.0087 (12)	0.0026 (10)
C28	0.0234 (10)	0.0138 (10)	0.0258 (11)	−0.0003 (8)	0.0065 (9)	0.0016 (8)
C29	0.0234 (11)	0.0234 (12)	0.0224 (11)	0.0018 (9)	0.0007 (9)	0.0004 (9)
C30	0.0216 (10)	0.0260 (12)	0.0220 (11)	0.0029 (9)	0.0010 (8)	0.0006 (9)
C31	0.0282 (12)	0.0247 (13)	0.0339 (14)	−0.0010 (10)	−0.0012 (10)	0.0050 (11)
C32	0.0263 (13)	0.0376 (16)	0.0382 (16)	−0.0050 (11)	−0.0038 (11)	0.0041 (13)
C33	0.0244 (12)	0.0424 (17)	0.0304 (14)	0.0055 (11)	−0.0023 (10)	−0.0014 (12)
C34	0.0322 (13)	0.0312 (14)	0.0254 (12)	0.0096 (11)	0.0025 (10)	0.0016 (10)
C35	0.0289 (12)	0.0237 (12)	0.0246 (11)	0.0038 (9)	0.0051 (9)	0.0026 (9)
C36	0.0296 (12)	0.0185 (11)	0.0204 (10)	−0.0057 (9)	0.0003 (9)	−0.0017 (8)
C37	0.0306 (12)	0.0196 (11)	0.0180 (10)	−0.0062 (9)	0.0008 (9)	−0.0018 (8)
C38	0.0340 (13)	0.0227 (12)	0.0261 (12)	−0.0026 (10)	0.0044 (10)	0.0004 (10)
C39	0.0318 (14)	0.0326 (15)	0.0322 (14)	−0.0012 (11)	0.0103 (11)	−0.0008 (11)
C40	0.0350 (14)	0.0359 (15)	0.0255 (12)	−0.0101 (12)	0.0092 (11)	−0.0029 (11)
C41	0.0420 (15)	0.0256 (13)	0.0206 (11)	−0.0092 (11)	0.0079 (10)	−0.0003 (9)
C42	0.0360 (13)	0.0203 (11)	0.0204 (11)	−0.0047 (10)	0.0032 (10)	0.0003 (9)
C43	0.0395 (14)	0.0190 (11)	0.0238 (11)	0.0041 (10)	0.0165 (10)	0.0035 (9)
C44	0.0375 (14)	0.0185 (11)	0.0280 (12)	0.0046 (10)	0.0171 (11)	0.0038 (9)
C45	0.0392 (15)	0.0206 (12)	0.0354 (14)	0.0032 (10)	0.0163 (12)	0.0011 (10)
C46	0.0390 (15)	0.0218 (13)	0.0471 (18)	0.0033 (11)	0.0157 (14)	0.0017 (12)
C47	0.0508 (19)	0.0258 (14)	0.0492 (19)	0.0122 (13)	0.0282 (16)	0.0025 (13)
C48	0.057 (2)	0.0253 (14)	0.0334 (15)	0.0121 (13)	0.0249 (14)	0.0018 (11)
C49	0.0488 (17)	0.0207 (12)	0.0277 (13)	0.0092 (11)	0.0158 (12)	0.0046 (10)
C50	0.055 (2)	0.0286 (15)	0.0258 (13)	0.0032 (13)	−0.0120 (13)	−0.0041 (11)
C51	0.062 (2)	0.0400 (19)	0.0284 (15)	0.0055 (16)	−0.0091 (15)	−0.0044 (13)
C52	0.059 (2)	0.042 (2)	0.048 (2)	−0.0021 (17)	0.0097 (18)	−0.0092 (16)
C53	0.0343 (14)	0.0271 (13)	0.0252 (12)	−0.0022 (10)	0.0069 (10)	−0.0024 (10)
C54	0.048 (2)	0.046 (2)	0.057 (2)	0.0014 (16)	0.0213 (18)	−0.0219 (18)
C55	0.048 (2)	0.066 (3)	0.0284 (15)	−0.0069 (18)	0.0088 (14)	0.0098 (16)
C56	0.051 (2)	0.083 (3)	0.0343 (18)	0.029 (2)	0.0017 (16)	−0.0045 (19)
C57	0.098 (4)	0.087 (4)	0.051 (3)	0.052 (4)	−0.015 (3)	−0.008 (3)
C58	0.073 (3)	0.118 (5)	0.066 (3)	0.053 (4)	−0.001 (3)	−0.040 (3)
C59	0.0301 (14)	0.0285 (15)	0.052 (2)	−0.0021 (11)	−0.0044 (13)	−0.0048 (13)
C60	0.057 (2)	0.0282 (16)	0.055 (2)	0.0054 (15)	0.0016 (18)	−0.0017 (15)
C61	0.054 (2)	0.072 (3)	0.050 (2)	0.017 (2)	−0.0142 (19)	−0.010 (2)
O27	0.036 (3)	0.0222 (17)	0.038 (3)	−0.0084 (14)	0.003 (2)	−0.0039 (16)
C62	0.0279 (17)	0.0263 (19)	0.035 (2)	−0.0040 (14)	−0.0005 (15)	0.0004 (16)
N9	0.0237 (14)	0.0196 (15)	0.0271 (16)	−0.0022 (12)	−0.0025 (12)	−0.0004 (12)
C63	0.037 (2)	0.031 (2)	0.045 (3)	0.0007 (17)	0.0017 (18)	0.0116 (19)
C64	0.041 (5)	0.055 (5)	0.037 (4)	0.002 (4)	−0.002 (3)	−0.006 (3)
O27B	0.049 (8)	0.073 (10)	0.061 (10)	−0.017 (8)	−0.004 (8)	−0.041 (8)
C62B	0.029 (4)	0.043 (5)	0.049 (5)	−0.006 (4)	−0.006 (4)	−0.004 (5)
N9B	0.029 (4)	0.050 (6)	0.063 (6)	−0.004 (4)	−0.002 (4)	−0.020 (5)
C63B	0.034 (6)	0.090 (11)	0.045 (7)	−0.007 (6)	−0.002 (5)	−0.022 (7)
C64B	0.030 (7)	0.024 (6)	0.051 (11)	−0.010 (5)	0.015 (8)	−0.023 (8)

### Tetra-µ-aqua-tris(µ-3-chlorobenzoato)(dimethylformamide)tetrakis(µ4-N,2-dioxidobenzene-1-carboximidato)pentamanganese(III)sodium(I) dimethylformamide tetraolvate 0.72-hydrate (2) Geometric parameters (Å, º)

**Table:**

Mn1—O15	2.123 (2)	C11—C12	1.400 (5)
Mn1—O13	2.127 (2)	C11—H11	0.9500
Mn1—O17	2.158 (2)	C12—C13	1.379 (4)
Mn1—O10	2.2439 (19)	C12—H12	0.9500
Mn1—O7	2.2496 (18)	C13—C14	1.412 (4)
Mn1—O1	2.3965 (17)	C13—H13	0.9500
Mn1—O4	2.4973 (18)	C15—C16	1.464 (3)
Mn1—Na1	3.3115 (12)	C16—C17	1.394 (3)
Mn2—O12	1.8585 (19)	C16—C21	1.412 (3)
Mn2—O1	1.9047 (17)	C17—C18	1.380 (4)
Mn2—O2	1.9310 (19)	C17—H17	0.9500
Mn2—N4	1.959 (2)	C18—C19	1.394 (5)
Mn2—O14	2.177 (2)	C18—H18	0.9500
Mn2—O19	2.491 (2)	C19—C20	1.370 (4)
Mn2—Na1	3.6455 (12)	C19—H19	0.9500
Mn3—O3	1.858 (2)	C20—C21	1.407 (3)
Mn3—O4	1.8888 (18)	C20—H20	0.9500
Mn3—O5	1.9366 (19)	C22—C23	1.470 (3)
Mn3—N1	1.952 (2)	C23—C24	1.398 (4)
Mn3—O23	2.216 (3)	C23—C28	1.411 (4)
Mn3—O20	2.414 (2)	C24—C25	1.384 (4)
Mn3—Na1	3.5489 (12)	C24—H24	0.9500
Mn4—O6	1.8523 (19)	C25—C26	1.400 (5)
Mn4—O7	1.9088 (17)	C25—H25	0.9500
Mn4—O8	1.9457 (18)	C26—C27	1.384 (4)
Mn4—N2	1.957 (2)	C26—H26	0.9500
Mn4—O16	2.156 (2)	C27—C28	1.405 (4)
Mn4—O21	2.501 (2)	C27—H27	0.9500
Mn4—Na1	3.6236 (12)	C29—C30	1.509 (4)
Mn5—O9	1.865 (2)	C30—C35	1.392 (4)
Mn5—O10	1.8997 (18)	C30—C31	1.401 (4)
Mn5—O11	1.9504 (18)	C31—C32	1.393 (4)
Mn5—N3	1.966 (2)	C31—H31	0.9500
Mn5—O18	2.138 (2)	C32—C33	1.383 (5)
Mn5—O22	2.444 (2)	C33—C34	1.385 (5)
Mn5—Na1	3.5750 (11)	C33—H33	0.9500
Cl1—C32	1.737 (3)	C34—C35	1.394 (4)
Cl2—C39	1.737 (3)	C34—H34	0.9500
Cl3—C46	1.737 (4)	C35—H35	0.9500
Na1—O22	2.401 (3)	C36—C37	1.507 (4)
Na1—O20	2.432 (2)	C37—C38	1.387 (4)
Na1—O19	2.519 (3)	C37—C42	1.399 (4)
Na1—O21	2.556 (3)	C38—C39	1.390 (4)
Na1—O7	2.572 (2)	C38—H38	0.9500
Na1—O1	2.589 (2)	C39—C40	1.385 (5)
Na1—O10	2.637 (2)	C40—C41	1.387 (5)
Na1—O4	2.669 (2)	C40—H40	0.9500
O1—N1	1.403 (2)	C41—C42	1.393 (4)
O2—C1	1.301 (3)	C41—H41	0.9500
O3—C7	1.330 (3)	C42—H42	0.9500
O4—N2	1.404 (3)	C43—C44	1.514 (4)
O5—C8	1.300 (3)	C44—C45	1.388 (5)
O6—C14	1.328 (3)	C44—C49	1.395 (4)
O7—N3	1.400 (2)	C45—C46	1.391 (4)
O8—C15	1.305 (3)	C45—H45	0.9500
O9—C21	1.339 (3)	C46—C47	1.389 (5)
O10—N4	1.400 (3)	C47—C48	1.393 (6)
O11—C22	1.301 (3)	C47—H47	0.9500
O12—C28	1.329 (3)	C48—C49	1.392 (4)
O13—C29	1.263 (3)	C48—H48	0.9500
O14—C29	1.260 (3)	C49—H49	0.9500
O15—C36	1.257 (3)	C50—H50	0.9500
O16—C36	1.253 (4)	C51—H51A	0.9800
O17—C43	1.257 (3)	C51—H51B	0.9800
O18—C43	1.257 (4)	C51—H51C	0.9800
O19—H19A	0.80 (2)	C52—H52A	0.9800
O19—H19B	0.83 (2)	C52—H52B	0.9800
O20—H20A	0.83 (2)	C52—H52C	0.9800
O20—H20B	0.86 (2)	C53—H53	0.9500
O21—H21A	0.83 (2)	C54—H54A	0.9800
O21—H21B	0.86 (2)	C54—H54B	0.9800
O22—H22A	0.84 (2)	C54—H54C	0.9800
O22—H22B	0.84 (2)	C55—H55A	0.9800
O23—C50	1.228 (5)	C55—H55B	0.9800
O24—C53	1.229 (4)	C55—H55C	0.9800
O25—C56	1.221 (5)	C56—H56	0.9500
O26—C59	1.242 (4)	C57—H57A	0.9800
O28—H28A	0.92 (2)	C57—H57B	0.9800
O28—H28B	0.90 (2)	C57—H57C	0.9800
N1—C1	1.317 (3)	C58—H58A	0.9800
N2—C8	1.321 (3)	C58—H58B	0.9800
N3—C15	1.309 (3)	C58—H58C	0.9800
N4—C22	1.310 (3)	C59—H59	0.9500
N5—C50	1.338 (5)	C60—H60A	0.9800
N5—C51	1.451 (4)	C60—H60B	0.9800
N5—C52	1.451 (5)	C60—H60C	0.9800
N6—C53	1.317 (4)	C61—H61A	0.9800
N6—C54	1.455 (5)	C61—H61B	0.9800
N6—C55	1.457 (5)	C61—H61C	0.9800
N7—C56	1.327 (6)	O27—C62	1.240 (7)
N7—C57	1.448 (6)	C62—N9	1.334 (5)
N7—C58	1.472 (6)	C62—H62	0.9500
N8—C59	1.317 (5)	N9—C63	1.435 (6)
N8—C60	1.447 (5)	N9—C64	1.479 (10)
N8—C61	1.462 (5)	C63—H63A	0.9800
C1—C2	1.473 (3)	C63—H63B	0.9800
C2—C7	1.405 (4)	C63—H63C	0.9800
C2—C3	1.405 (4)	C64—H64A	0.9800
C3—C4	1.389 (4)	C64—H64B	0.9800
C3—H3	0.9500	C64—H64C	0.9800
C4—C5	1.388 (5)	O27B—C62B	1.193 (19)
C4—H4	0.9500	C62B—N9B	1.329 (16)
C5—C6	1.387 (4)	C62B—H62B	0.9500
C5—H5	0.9500	N9B—C64B	1.462 (17)
C6—C7	1.411 (4)	N9B—C63B	1.492 (19)
C6—H6	0.9500	C63B—H63D	0.9800
C8—C9	1.468 (4)	C63B—H63E	0.9800
C9—C10	1.397 (4)	C63B—H63F	0.9800
C9—C14	1.409 (4)	C64B—H64D	0.9800
C10—C11	1.383 (4)	C64B—H64E	0.9800
C10—H10	0.9500	C64B—H64F	0.9800
			
O15—Mn1—O13	93.78 (9)	C50—N5—C52	121.7 (3)
O15—Mn1—O17	83.90 (8)	C51—N5—C52	118.0 (3)
O13—Mn1—O17	86.26 (10)	C53—N6—C54	119.8 (3)
O15—Mn1—O10	154.16 (7)	C53—N6—C55	122.0 (3)
O13—Mn1—O10	106.98 (8)	C54—N6—C55	118.1 (3)
O17—Mn1—O10	82.32 (8)	C56—N7—C57	120.6 (4)
O15—Mn1—O7	88.08 (7)	C56—N7—C58	120.3 (4)
O13—Mn1—O7	175.86 (9)	C57—N7—C58	118.7 (4)
O17—Mn1—O7	90.26 (8)	C59—N8—C60	122.6 (3)
O10—Mn1—O7	70.26 (6)	C59—N8—C61	120.4 (3)
O15—Mn1—O1	131.86 (7)	C60—N8—C61	117.0 (4)
O13—Mn1—O1	79.95 (8)	O2—C1—N1	120.1 (2)
O17—Mn1—O1	142.05 (7)	O2—C1—C2	119.7 (2)
O10—Mn1—O1	68.52 (6)	N1—C1—C2	120.2 (2)
O7—Mn1—O1	101.55 (6)	C7—C2—C3	120.0 (2)
O15—Mn1—O4	78.10 (7)	C7—C2—C1	122.8 (2)
O13—Mn1—O4	116.73 (8)	C3—C2—C1	117.2 (2)
O17—Mn1—O4	151.39 (8)	C4—C3—C2	121.2 (3)
O10—Mn1—O4	104.86 (7)	C4—C3—H3	119.4
O7—Mn1—O4	67.28 (6)	C2—C3—H3	119.4
O1—Mn1—O4	63.44 (6)	C5—C4—C3	118.7 (3)
O15—Mn1—Na1	123.17 (6)	C5—C4—H4	120.7
O13—Mn1—Na1	130.21 (6)	C3—C4—H4	120.7
O17—Mn1—Na1	125.97 (7)	C6—C5—C4	121.2 (3)
O10—Mn1—Na1	52.50 (5)	C6—C5—H5	119.4
O7—Mn1—Na1	50.83 (5)	C4—C5—H5	119.4
O1—Mn1—Na1	50.92 (5)	C5—C6—C7	120.8 (3)
O4—Mn1—Na1	52.46 (5)	C5—C6—H6	119.6
O12—Mn2—O1	173.08 (9)	C7—C6—H6	119.6
O12—Mn2—O2	96.07 (8)	O3—C7—C2	124.6 (2)
O1—Mn2—O2	82.34 (8)	O3—C7—C6	117.1 (3)
O12—Mn2—N4	89.86 (8)	C2—C7—C6	118.1 (3)
O1—Mn2—N4	90.57 (8)	O5—C8—N2	119.9 (2)
O2—Mn2—N4	168.31 (8)	O5—C8—C9	119.8 (2)
O12—Mn2—O14	95.23 (9)	N2—C8—C9	120.3 (2)
O1—Mn2—O14	91.62 (8)	C10—C9—C14	119.9 (2)
O2—Mn2—O14	94.59 (8)	C10—C9—C8	117.4 (2)
N4—Mn2—O14	94.89 (8)	C14—C9—C8	122.7 (2)
O12—Mn2—O19	94.11 (9)	C11—C10—C9	121.3 (3)
O1—Mn2—O19	79.10 (8)	C11—C10—H10	119.3
O2—Mn2—O19	86.99 (8)	C9—C10—H10	119.3
N4—Mn2—O19	82.53 (8)	C10—C11—C12	119.0 (3)
O14—Mn2—O19	170.31 (8)	C10—C11—H11	120.5
O12—Mn2—Na1	131.87 (7)	C12—C11—H11	120.5
O1—Mn2—Na1	42.64 (6)	C13—C12—C11	120.6 (3)
O2—Mn2—Na1	101.83 (6)	C13—C12—H12	119.7
N4—Mn2—Na1	66.90 (6)	C11—C12—H12	119.7
O14—Mn2—Na1	126.85 (6)	C12—C13—C14	121.0 (3)
O19—Mn2—Na1	43.60 (5)	C12—C13—H13	119.5
O3—Mn3—O4	176.78 (11)	C14—C13—H13	119.5
O3—Mn3—O5	97.50 (9)	O6—C14—C9	125.0 (2)
O4—Mn3—O5	82.68 (8)	O6—C14—C13	116.8 (2)
O3—Mn3—N1	91.49 (9)	C9—C14—C13	118.2 (2)
O4—Mn3—N1	87.88 (8)	O8—C15—N3	120.4 (2)
O5—Mn3—N1	167.83 (8)	O8—C15—C16	119.9 (2)
O3—Mn3—O23	88.03 (11)	N3—C15—C16	119.7 (2)
O4—Mn3—O23	95.18 (10)	C17—C16—C21	119.5 (2)
O5—Mn3—O23	92.36 (10)	C17—C16—C15	117.0 (2)
N1—Mn3—O23	96.15 (9)	C21—C16—C15	123.5 (2)
O3—Mn3—O20	92.93 (10)	C18—C17—C16	121.5 (3)
O4—Mn3—O20	83.86 (8)	C18—C17—H17	119.2
O5—Mn3—O20	87.05 (9)	C16—C17—H17	119.2
N1—Mn3—O20	84.30 (8)	C17—C18—C19	118.6 (3)
O23—Mn3—O20	178.93 (9)	C17—C18—H18	120.7
O3—Mn3—Na1	129.36 (9)	C19—C18—H18	120.7
O4—Mn3—Na1	47.62 (7)	C20—C19—C18	121.2 (3)
O5—Mn3—Na1	102.36 (6)	C20—C19—H19	119.4
N1—Mn3—Na1	65.50 (6)	C18—C19—H19	119.4
O23—Mn3—Na1	136.27 (7)	C19—C20—C21	120.8 (3)
O20—Mn3—Na1	43.10 (5)	C19—C20—H20	119.6
O6—Mn4—O7	171.16 (9)	C21—C20—H20	119.6
O6—Mn4—O8	95.88 (8)	O9—C21—C20	117.5 (2)
O7—Mn4—O8	81.92 (7)	O9—C21—C16	124.2 (2)
O6—Mn4—N2	90.97 (8)	C20—C21—C16	118.3 (2)
O7—Mn4—N2	89.88 (8)	O11—C22—N4	120.3 (2)
O8—Mn4—N2	168.33 (9)	O11—C22—C23	120.3 (2)
O6—Mn4—O16	95.41 (9)	N4—C22—C23	119.3 (2)
O7—Mn4—O16	93.26 (8)	C24—C23—C28	119.8 (2)
O8—Mn4—O16	93.06 (8)	C24—C23—C22	118.0 (2)
N2—Mn4—O16	95.69 (9)	C28—C23—C22	122.2 (2)
O6—Mn4—O21	91.92 (9)	C25—C24—C23	121.6 (3)
O7—Mn4—O21	79.48 (8)	C25—C24—H24	119.2
O8—Mn4—O21	88.28 (8)	C23—C24—H24	119.2
N2—Mn4—O21	82.04 (9)	C24—C25—C26	119.0 (3)
O16—Mn4—O21	172.37 (8)	C24—C25—H25	120.5
O6—Mn4—Na1	130.59 (7)	C26—C25—H25	120.5
O7—Mn4—Na1	42.75 (6)	C27—C26—C25	120.1 (3)
O8—Mn4—Na1	103.60 (6)	C27—C26—H26	120.0
N2—Mn4—Na1	64.87 (7)	C25—C26—H26	120.0
O16—Mn4—Na1	127.67 (6)	C26—C27—C28	121.7 (3)
O21—Mn4—Na1	44.83 (6)	C26—C27—H27	119.2
O9—Mn5—O10	171.38 (10)	C28—C27—H27	119.2
O9—Mn5—O11	98.50 (9)	O12—C28—C27	117.7 (2)
O10—Mn5—O11	81.66 (8)	O12—C28—C23	124.4 (2)
O9—Mn5—N3	91.18 (8)	C27—C28—C23	118.0 (2)
O10—Mn5—N3	87.72 (8)	O14—C29—O13	126.7 (3)
O11—Mn5—N3	168.08 (8)	O14—C29—C30	116.5 (2)
O9—Mn5—O18	94.05 (10)	O13—C29—C30	116.8 (2)
O10—Mn5—O18	94.57 (9)	C35—C30—C31	119.6 (3)
O11—Mn5—O18	90.32 (9)	C35—C30—C29	121.3 (2)
N3—Mn5—O18	95.95 (9)	C31—C30—C29	119.0 (2)
O9—Mn5—O22	90.05 (9)	C32—C31—C30	119.0 (3)
O10—Mn5—O22	81.33 (8)	C32—C31—H31	120.5
O11—Mn5—O22	90.71 (8)	C30—C31—H31	120.5
N3—Mn5—O22	82.29 (8)	C33—C32—C31	121.5 (3)
O18—Mn5—O22	175.58 (8)	C33—C32—Cl1	120.0 (2)
O9—Mn5—Na1	126.17 (7)	C31—C32—Cl1	118.5 (3)
O10—Mn5—Na1	46.01 (6)	C32—C33—C34	119.1 (3)
O11—Mn5—Na1	103.58 (6)	C32—C33—H33	120.4
N3—Mn5—Na1	64.84 (6)	C34—C33—H33	120.4
O18—Mn5—Na1	133.61 (6)	C33—C34—C35	120.4 (3)
O22—Mn5—Na1	41.99 (6)	C33—C34—H34	119.8
O22—Na1—O20	142.14 (9)	C35—C34—H34	119.8
O22—Na1—O19	84.23 (8)	C30—C35—C34	120.2 (3)
O20—Na1—O19	86.48 (8)	C30—C35—H35	119.9
O22—Na1—O21	84.10 (8)	C34—C35—H35	119.9
O20—Na1—O21	84.80 (8)	O16—C36—O15	126.4 (3)
O19—Na1—O21	148.18 (9)	O16—C36—C37	117.1 (2)
O22—Na1—O7	84.00 (7)	O15—C36—C37	116.5 (2)
O20—Na1—O7	124.20 (8)	C38—C37—C42	120.0 (3)
O19—Na1—O7	139.85 (8)	C38—C37—C36	118.9 (2)
O21—Na1—O7	67.70 (7)	C42—C37—C36	121.1 (3)
O22—Na1—O1	124.38 (8)	C37—C38—C39	119.4 (3)
O20—Na1—O1	84.69 (7)	C37—C38—H38	120.3
O19—Na1—O1	67.42 (7)	C39—C38—H38	120.3
O21—Na1—O1	141.60 (8)	C40—C39—C38	121.4 (3)
O7—Na1—O1	88.47 (6)	C40—C39—Cl2	120.2 (2)
O22—Na1—O10	69.00 (7)	C38—C39—Cl2	118.4 (2)
O20—Na1—O10	144.74 (8)	C39—C40—C41	118.8 (3)
O19—Na1—O10	80.45 (7)	C39—C40—H40	120.6
O21—Na1—O10	122.08 (7)	C41—C40—H40	120.6
O7—Na1—O10	59.51 (6)	C40—C41—C42	120.8 (3)
O1—Na1—O10	60.05 (6)	C40—C41—H41	119.6
O22—Na1—O4	144.38 (8)	C42—C41—H41	119.6
O20—Na1—O4	69.17 (7)	C41—C42—C37	119.5 (3)
O19—Na1—O4	121.77 (8)	C41—C42—H42	120.2
O21—Na1—O4	83.15 (7)	C37—C42—H42	120.2
O7—Na1—O4	60.39 (6)	O17—C43—O18	126.4 (3)
O1—Na1—O4	58.61 (6)	O17—C43—C44	116.4 (3)
O10—Na1—O4	90.27 (6)	O18—C43—C44	117.2 (2)
O22—Na1—Mn1	105.39 (6)	C45—C44—C49	120.2 (3)
O20—Na1—Mn1	112.47 (7)	C45—C44—C43	119.0 (3)
O19—Na1—Mn1	105.23 (6)	C49—C44—C43	120.8 (3)
O21—Na1—Mn1	106.39 (6)	C44—C45—C46	119.6 (3)
O7—Na1—Mn1	42.69 (4)	C44—C45—H45	120.2
O1—Na1—Mn1	45.93 (4)	C46—C45—H45	120.2
O10—Na1—Mn1	42.46 (4)	C47—C46—C45	121.0 (4)
O4—Na1—Mn1	47.89 (4)	C47—C46—Cl3	119.7 (3)
O22—Na1—Mn3	175.12 (7)	C45—C46—Cl3	119.3 (3)
O20—Na1—Mn3	42.72 (6)	C46—C47—C48	118.8 (3)
O19—Na1—Mn3	97.36 (6)	C46—C47—H47	120.6
O21—Na1—Mn3	96.76 (6)	C48—C47—H47	120.6
O7—Na1—Mn3	91.86 (5)	C49—C48—C47	120.9 (3)
O1—Na1—Mn3	52.69 (4)	C49—C48—H48	119.6
O10—Na1—Mn3	106.64 (5)	C47—C48—H48	119.6
O4—Na1—Mn3	31.51 (4)	C48—C49—C44	119.5 (3)
Mn1—Na1—Mn3	69.75 (2)	C48—C49—H49	120.3
O22—Na1—Mn5	42.92 (6)	C44—C49—H49	120.3
O20—Na1—Mn5	174.94 (7)	O23—C50—N5	124.6 (3)
O19—Na1—Mn5	94.60 (6)	O23—C50—H50	117.7
O21—Na1—Mn5	96.74 (6)	N5—C50—H50	117.7
O7—Na1—Mn5	52.62 (4)	N5—C51—H51A	109.5
O1—Na1—Mn5	91.16 (5)	N5—C51—H51B	109.5
O10—Na1—Mn5	31.21 (4)	H51A—C51—H51B	109.5
O4—Na1—Mn5	106.18 (5)	N5—C51—H51C	109.5
Mn1—Na1—Mn5	62.48 (2)	H51A—C51—H51C	109.5
Mn3—Na1—Mn5	132.22 (3)	H51B—C51—H51C	109.5
O22—Na1—Mn4	98.29 (6)	N5—C52—H52A	109.5
O20—Na1—Mn4	98.23 (6)	N5—C52—H52B	109.5
O19—Na1—Mn4	168.10 (7)	H52A—C52—H52B	109.5
O21—Na1—Mn4	43.63 (5)	N5—C52—H52C	109.5
O7—Na1—Mn4	30.25 (4)	H52A—C52—H52C	109.5
O1—Na1—Mn4	101.99 (5)	H52B—C52—H52C	109.5
O10—Na1—Mn4	89.57 (5)	O24—C53—N6	125.5 (3)
O4—Na1—Mn4	51.29 (4)	O24—C53—H53	117.2
Mn1—Na1—Mn4	62.87 (2)	N6—C53—H53	117.2
Mn3—Na1—Mn4	79.22 (3)	N6—C54—H54A	109.5
Mn5—Na1—Mn4	79.77 (2)	N6—C54—H54B	109.5
N1—O1—Mn2	112.41 (13)	H54A—C54—H54B	109.5
N1—O1—Mn1	127.14 (13)	N6—C54—H54C	109.5
Mn2—O1—Mn1	113.88 (7)	H54A—C54—H54C	109.5
N1—O1—Na1	105.87 (13)	H54B—C54—H54C	109.5
Mn2—O1—Na1	107.46 (8)	N6—C55—H55A	109.5
Mn1—O1—Na1	83.15 (6)	N6—C55—H55B	109.5
C1—O2—Mn2	112.00 (15)	H55A—C55—H55B	109.5
C7—O3—Mn3	128.82 (18)	N6—C55—H55C	109.5
N2—O4—Mn3	112.50 (13)	H55A—C55—H55C	109.5
N2—O4—Mn1	119.12 (13)	H55B—C55—H55C	109.5
Mn3—O4—Mn1	126.57 (8)	O25—C56—N7	125.9 (4)
N2—O4—Na1	104.23 (14)	O25—C56—H56	117.0
Mn3—O4—Na1	100.87 (8)	N7—C56—H56	117.0
Mn1—O4—Na1	79.65 (6)	N7—C57—H57A	109.5
C8—O5—Mn3	111.48 (16)	N7—C57—H57B	109.5
C14—O6—Mn4	130.01 (16)	H57A—C57—H57B	109.5
N3—O7—Mn4	112.74 (13)	N7—C57—H57C	109.5
N3—O7—Mn1	117.20 (13)	H57A—C57—H57C	109.5
Mn4—O7—Mn1	121.20 (8)	H57B—C57—H57C	109.5
N3—O7—Na1	106.89 (13)	N7—C58—H58A	109.5
Mn4—O7—Na1	107.00 (8)	N7—C58—H58B	109.5
Mn1—O7—Na1	86.48 (6)	H58A—C58—H58B	109.5
C15—O8—Mn4	111.56 (15)	N7—C58—H58C	109.5
C21—O9—Mn5	127.58 (17)	H58A—C58—H58C	109.5
N4—O10—Mn5	113.34 (13)	H58B—C58—H58C	109.5
N4—O10—Mn1	119.59 (14)	O26—C59—N8	125.8 (3)
Mn5—O10—Mn1	119.22 (8)	O26—C59—H59	117.1
N4—O10—Na1	110.70 (13)	N8—C59—H59	117.1
Mn5—O10—Na1	102.78 (8)	N8—C60—H60A	109.5
Mn1—O10—Na1	85.04 (6)	N8—C60—H60B	109.5
C22—O11—Mn5	111.76 (16)	H60A—C60—H60B	109.5
C28—O12—Mn2	130.52 (16)	N8—C60—H60C	109.5
C29—O13—Mn1	138.1 (2)	H60A—C60—H60C	109.5
C29—O14—Mn2	130.32 (18)	H60B—C60—H60C	109.5
C36—O15—Mn1	133.04 (17)	N8—C61—H61A	109.5
C36—O16—Mn4	125.69 (18)	N8—C61—H61B	109.5
C43—O17—Mn1	140.5 (2)	H61A—C61—H61B	109.5
C43—O18—Mn5	122.73 (17)	N8—C61—H61C	109.5
Mn2—O19—Na1	93.38 (8)	H61A—C61—H61C	109.5
Mn2—O19—H19A	112 (3)	H61B—C61—H61C	109.5
Na1—O19—H19A	116 (3)	O27—C62—N9	123.4 (5)
Mn2—O19—H19B	122 (3)	O27—C62—H62	118.3
Na1—O19—H19B	109 (3)	N9—C62—H62	118.3
H19A—O19—H19B	105 (5)	C62—N9—C63	121.7 (4)
Mn3—O20—Na1	94.17 (8)	C62—N9—C64	117.3 (6)
Mn3—O20—H20A	110 (3)	C63—N9—C64	120.9 (6)
Na1—O20—H20A	113 (3)	N9—C63—H63A	109.5
Mn3—O20—H20B	109 (3)	N9—C63—H63B	109.5
Na1—O20—H20B	120 (3)	H63A—C63—H63B	109.5
H20A—O20—H20B	109 (5)	N9—C63—H63C	109.5
Mn4—O21—Na1	91.54 (7)	H63A—C63—H63C	109.5
Mn4—O21—H21A	113 (3)	H63B—C63—H63C	109.5
Na1—O21—H21A	115 (3)	N9—C64—H64A	109.5
Mn4—O21—H21B	118 (3)	N9—C64—H64B	109.5
Na1—O21—H21B	127 (3)	H64A—C64—H64B	109.5
H21A—O21—H21B	93 (4)	N9—C64—H64C	109.5
Na1—O22—Mn5	95.09 (8)	H64A—C64—H64C	109.5
Na1—O22—H22A	118 (3)	H64B—C64—H64C	109.5
Mn5—O22—H22A	108 (3)	O27B—C62B—N9B	131.0 (18)
Na1—O22—H22B	113 (3)	O27B—C62B—H62B	114.5
Mn5—O22—H22B	117 (3)	N9B—C62B—H62B	114.5
H22A—O22—H22B	107 (4)	C62B—N9B—C64B	127.5 (18)
C50—O23—Mn3	125.9 (2)	C62B—N9B—C63B	115.5 (12)
H28A—O28—H28B	111 (8)	C64B—N9B—C63B	117.0 (16)
C1—N1—O1	113.09 (19)	N9B—C63B—H63D	109.5
C1—N1—Mn3	129.69 (16)	N9B—C63B—H63E	109.5
O1—N1—Mn3	115.75 (14)	H63D—C63B—H63E	109.5
C8—N2—O4	113.12 (19)	N9B—C63B—H63F	109.5
C8—N2—Mn4	129.71 (17)	H63D—C63B—H63F	109.5
O4—N2—Mn4	115.41 (14)	H63E—C63B—H63F	109.5
C15—N3—O7	113.19 (18)	N9B—C64B—H64D	109.5
C15—N3—Mn5	130.03 (16)	N9B—C64B—H64E	109.5
O7—N3—Mn5	115.91 (14)	H64D—C64B—H64E	109.5
C22—N4—O10	112.91 (19)	N9B—C64B—H64F	109.5
C22—N4—Mn2	130.57 (17)	H64D—C64B—H64F	109.5
O10—N4—Mn2	113.66 (14)	H64E—C64B—H64F	109.5
C50—N5—C51	120.1 (3)		
			
O5—Mn3—O3—C7	−178.3 (3)	C12—C13—C14—O6	−179.0 (3)
N1—Mn3—O3—C7	9.9 (3)	C12—C13—C14—C9	1.0 (5)
O23—Mn3—O3—C7	−86.2 (3)	Mn4—O8—C15—N3	−3.9 (3)
O20—Mn3—O3—C7	94.3 (3)	Mn4—O8—C15—C16	177.92 (17)
Na1—Mn3—O3—C7	68.9 (3)	O7—N3—C15—O8	1.4 (3)
O5—Mn3—O4—N2	−3.66 (17)	Mn5—N3—C15—O8	170.19 (17)
N1—Mn3—O4—N2	168.64 (17)	O7—N3—C15—C16	179.62 (19)
O23—Mn3—O4—N2	−95.38 (18)	Mn5—N3—C15—C16	−11.6 (3)
O20—Mn3—O4—N2	84.15 (17)	O8—C15—C16—C17	12.6 (3)
Na1—Mn3—O4—N2	110.51 (18)	N3—C15—C16—C17	−165.6 (2)
O5—Mn3—O4—Mn1	160.73 (13)	O8—C15—C16—C21	−168.0 (2)
N1—Mn3—O4—Mn1	−26.98 (12)	N3—C15—C16—C21	13.8 (4)
O23—Mn3—O4—Mn1	69.01 (13)	C21—C16—C17—C18	−0.5 (4)
O20—Mn3—O4—Mn1	−111.47 (12)	C15—C16—C17—C18	178.8 (3)
Na1—Mn3—O4—Mn1	−85.10 (11)	C16—C17—C18—C19	2.6 (5)
O5—Mn3—O4—Na1	−114.17 (9)	C17—C18—C19—C20	−2.5 (6)
N1—Mn3—O4—Na1	58.13 (8)	C18—C19—C20—C21	0.3 (5)
O23—Mn3—O4—Na1	154.11 (9)	Mn5—O9—C21—C20	162.5 (2)
O20—Mn3—O4—Na1	−26.36 (7)	Mn5—O9—C21—C16	−20.8 (4)
O8—Mn4—O6—C14	165.9 (2)	C19—C20—C21—O9	178.7 (3)
N2—Mn4—O6—C14	−4.7 (3)	C19—C20—C21—C16	1.8 (4)
O16—Mn4—O6—C14	−100.5 (3)	C17—C16—C21—O9	−178.4 (3)
O21—Mn4—O6—C14	77.4 (3)	C15—C16—C21—O9	2.3 (4)
Na1—Mn4—O6—C14	52.3 (3)	C17—C16—C21—C20	−1.7 (4)
O11—Mn5—O9—C21	−168.8 (2)	C15—C16—C21—C20	179.0 (2)
N3—Mn5—O9—C21	18.2 (2)	Mn5—O11—C22—N4	0.5 (3)
O18—Mn5—O9—C21	−77.8 (2)	Mn5—O11—C22—C23	−177.99 (18)
O22—Mn5—O9—C21	100.5 (2)	O10—N4—C22—O11	0.7 (3)
Na1—Mn5—O9—C21	77.5 (2)	Mn2—N4—C22—O11	159.98 (19)
O11—Mn5—O10—N4	1.38 (15)	O10—N4—C22—C23	179.1 (2)
N3—Mn5—O10—N4	175.94 (16)	Mn2—N4—C22—C23	−21.6 (3)
O18—Mn5—O10—N4	−88.27 (16)	O11—C22—C23—C24	14.8 (4)
O22—Mn5—O10—N4	93.39 (15)	N4—C22—C23—C24	−163.7 (2)
Na1—Mn5—O10—N4	119.51 (17)	O11—C22—C23—C28	−166.7 (2)
O11—Mn5—O10—Mn1	150.42 (11)	N4—C22—C23—C28	14.8 (4)
N3—Mn5—O10—Mn1	−35.02 (10)	C28—C23—C24—C25	1.4 (4)
O18—Mn5—O10—Mn1	60.77 (11)	C22—C23—C24—C25	179.9 (3)
O22—Mn5—O10—Mn1	−117.57 (10)	C23—C24—C25—C26	0.0 (4)
Na1—Mn5—O10—Mn1	−91.45 (10)	C24—C25—C26—C27	−0.6 (5)
O11—Mn5—O10—Na1	−118.13 (9)	C25—C26—C27—C28	−0.2 (5)
N3—Mn5—O10—Na1	56.43 (8)	Mn2—O12—C28—C27	170.5 (2)
O18—Mn5—O10—Na1	152.22 (8)	Mn2—O12—C28—C23	−11.2 (4)
O22—Mn5—O10—Na1	−26.12 (7)	C26—C27—C28—O12	−180.0 (3)
O2—Mn2—O12—C28	175.4 (2)	C26—C27—C28—C23	1.6 (4)
N4—Mn2—O12—C28	5.4 (2)	C24—C23—C28—O12	179.5 (2)
O14—Mn2—O12—C28	−89.4 (2)	C22—C23—C28—O12	1.1 (4)
O19—Mn2—O12—C28	88.0 (2)	C24—C23—C28—C27	−2.1 (4)
Na1—Mn2—O12—C28	63.5 (3)	C22—C23—C28—C27	179.4 (2)
Mn2—O1—N1—C1	−1.0 (2)	Mn2—O14—C29—O13	−8.8 (4)
Mn1—O1—N1—C1	−150.50 (16)	Mn2—O14—C29—C30	170.39 (17)
Na1—O1—N1—C1	116.06 (17)	Mn1—O13—C29—O14	−11.7 (5)
Mn2—O1—N1—Mn3	−168.51 (10)	Mn1—O13—C29—C30	169.1 (2)
Mn1—O1—N1—Mn3	42.0 (2)	O14—C29—C30—C35	−3.0 (4)
Na1—O1—N1—Mn3	−51.46 (16)	O13—C29—C30—C35	176.3 (3)
Mn3—O4—N2—C8	1.8 (3)	O14—C29—C30—C31	179.3 (3)
Mn1—O4—N2—C8	−163.82 (18)	O13—C29—C30—C31	−1.4 (4)
Na1—O4—N2—C8	110.2 (2)	C35—C30—C31—C32	−0.7 (4)
Mn3—O4—N2—Mn4	−164.55 (11)	C29—C30—C31—C32	177.1 (3)
Mn1—O4—N2—Mn4	29.8 (2)	C30—C31—C32—C33	1.1 (5)
Na1—O4—N2—Mn4	−56.15 (16)	C30—C31—C32—Cl1	−177.4 (2)
Mn4—O7—N3—C15	1.8 (2)	C31—C32—C33—C34	−1.0 (5)
Mn1—O7—N3—C15	−146.06 (16)	Cl1—C32—C33—C34	177.5 (3)
Na1—O7—N3—C15	119.13 (17)	C32—C33—C34—C35	0.4 (5)
Mn4—O7—N3—Mn5	−168.60 (9)	C31—C30—C35—C34	0.1 (4)
Mn1—O7—N3—Mn5	43.51 (18)	C29—C30—C35—C34	−177.6 (3)
Na1—O7—N3—Mn5	−51.30 (16)	C33—C34—C35—C30	0.0 (4)
Mn5—O10—N4—C22	−1.5 (2)	Mn4—O16—C36—O15	−4.6 (4)
Mn1—O10—N4—C22	−150.44 (16)	Mn4—O16—C36—C37	175.17 (17)
Na1—O10—N4—C22	113.35 (18)	Mn1—O15—C36—O16	50.2 (4)
Mn5—O10—N4—Mn2	−164.47 (9)	Mn1—O15—C36—C37	−129.6 (2)
Mn1—O10—N4—Mn2	46.61 (19)	O16—C36—C37—C38	−161.2 (3)
Na1—O10—N4—Mn2	−49.60 (16)	O15—C36—C37—C38	18.6 (4)
Mn2—O2—C1—N1	−3.8 (3)	O16—C36—C37—C42	19.1 (4)
Mn2—O2—C1—C2	178.39 (17)	O15—C36—C37—C42	−161.2 (2)
O1—N1—C1—O2	3.2 (3)	C42—C37—C38—C39	0.4 (4)
Mn3—N1—C1—O2	168.57 (18)	C36—C37—C38—C39	−179.4 (3)
O1—N1—C1—C2	−179.0 (2)	C37—C38—C39—C40	0.9 (5)
Mn3—N1—C1—C2	−13.6 (3)	C37—C38—C39—Cl2	−176.9 (2)
O2—C1—C2—C7	−174.0 (2)	C38—C39—C40—C41	−1.5 (5)
N1—C1—C2—C7	8.2 (4)	Cl2—C39—C40—C41	176.2 (2)
O2—C1—C2—C3	7.2 (3)	C39—C40—C41—C42	0.9 (4)
N1—C1—C2—C3	−170.6 (2)	C40—C41—C42—C37	0.3 (4)
C7—C2—C3—C4	−1.8 (4)	C38—C37—C42—C41	−0.9 (4)
C1—C2—C3—C4	176.9 (3)	C36—C37—C42—C41	178.8 (2)
C2—C3—C4—C5	−0.3 (5)	Mn1—O17—C43—O18	42.8 (5)
C3—C4—C5—C6	1.7 (5)	Mn1—O17—C43—C44	−137.4 (3)
C4—C5—C6—C7	−0.9 (5)	Mn5—O18—C43—O17	−16.1 (4)
Mn3—O3—C7—C2	−16.7 (5)	Mn5—O18—C43—C44	164.12 (18)
Mn3—O3—C7—C6	166.4 (2)	O17—C43—C44—C45	18.1 (4)
C3—C2—C7—O3	−174.3 (3)	O18—C43—C44—C45	−162.1 (3)
C1—C2—C7—O3	7.0 (4)	O17—C43—C44—C49	−163.8 (3)
C3—C2—C7—C6	2.6 (4)	O18—C43—C44—C49	16.0 (4)
C1—C2—C7—C6	−176.1 (3)	C49—C44—C45—C46	0.2 (4)
C5—C6—C7—O3	175.9 (3)	C43—C44—C45—C46	178.3 (3)
C5—C6—C7—C2	−1.2 (5)	C44—C45—C46—C47	1.7 (5)
Mn3—O5—C8—N2	−5.5 (3)	C44—C45—C46—Cl3	−178.6 (2)
Mn3—O5—C8—C9	175.6 (2)	C45—C46—C47—C48	−1.8 (5)
O4—N2—C8—O5	2.5 (4)	Cl3—C46—C47—C48	178.5 (3)
Mn4—N2—C8—O5	166.5 (2)	C46—C47—C48—C49	0.0 (5)
O4—N2—C8—C9	−178.6 (2)	C47—C48—C49—C44	1.9 (5)
Mn4—N2—C8—C9	−14.7 (4)	C45—C44—C49—C48	−2.0 (4)
O5—C8—C9—C10	6.6 (4)	C43—C44—C49—C48	180.0 (3)
N2—C8—C9—C10	−172.2 (3)	Mn3—O23—C50—N5	−176.9 (2)
O5—C8—C9—C14	−174.9 (3)	C51—N5—C50—O23	0.6 (5)
N2—C8—C9—C14	6.3 (4)	C52—N5—C50—O23	175.9 (4)
C14—C9—C10—C11	−0.6 (6)	C54—N6—C53—O24	−1.9 (5)
C8—C9—C10—C11	178.0 (4)	C55—N6—C53—O24	−177.9 (3)
C9—C10—C11—C12	0.7 (7)	C57—N7—C56—O25	−5.2 (9)
C10—C11—C12—C13	0.1 (8)	C58—N7—C56—O25	−177.5 (6)
C11—C12—C13—C14	−1.0 (7)	C60—N8—C59—O26	0.1 (6)
Mn4—O6—C14—C9	−0.4 (4)	C61—N8—C59—O26	177.3 (4)
Mn4—O6—C14—C13	179.6 (2)	O27—C62—N9—C63	0.6 (8)
C10—C9—C14—O6	179.8 (3)	O27—C62—N9—C64	−176.8 (9)
C8—C9—C14—O6	1.3 (5)	O27B—C62B—N9B—C64B	−174 (3)
C10—C9—C14—C13	−0.3 (4)	O27B—C62B—N9B—C63B	5 (3)
C8—C9—C14—C13	−178.7 (3)		

### Tetra-µ-aqua-tris(µ-3-chlorobenzoato)(dimethylformamide)tetrakis(µ4-N,2-dioxidobenzene-1-carboximidato)pentamanganese(III)sodium(I) dimethylformamide tetraolvate 0.72-hydrate (2) Hydrogen-bond geometry (Å, º)

**Table:**

*D*—H···*A*	*D*—H	H···*A*	*D*···*A*	*D*—H···*A*
O19—H19*A*···O24	0.80 (2)	2.00 (3)	2.749 (3)	155 (5)
O19—H19*B*···O27	0.83 (2)	2.03 (3)	2.826 (7)	161 (5)
O20—H20*A*···O24	0.83 (2)	2.00 (3)	2.788 (3)	160 (5)
O20—H20*B*···O25	0.86 (2)	1.96 (3)	2.751 (4)	153 (4)
O21—H21*A*···O26	0.83 (2)	1.96 (3)	2.737 (3)	155 (5)
O21—H21*B*···O25	0.86 (2)	2.10 (4)	2.787 (3)	136 (4)
O22—H22*A*···O26	0.84 (2)	1.93 (3)	2.729 (3)	158 (5)
O22—H22*B*···O27	0.84 (2)	1.90 (3)	2.705 (6)	160 (5)
O28—H28*A*···Cl3^i^	0.92 (2)	2.96 (2)	3.876 (5)	171 (6)
O28—H28*B*···O9	0.90 (2)	2.14 (2)	3.019 (5)	165 (8)

Symmetry code: (i) *x*−1, *y*, *z*.
